# Reciprocal regulation by the CepIR and CciIR quorum sensing systems in *Burkholderia cenocepacia*

**DOI:** 10.1186/1471-2164-10-441

**Published:** 2009-09-17

**Authors:** Eoin P O'Grady, Duber F Viteri, Rebecca J Malott, Pamela A Sokol

**Affiliations:** 1 Department of Microbiology and Infectious Diseases, University of Calgary, Calgary, Alberta, Canada; 2 Current address: Department of Molecular Genetics, University of Toronto, Toronto, Ontario, Canada

## Abstract

**Background:**

*Burkholderia cenocepacia *belongs to a group of closely related organisms called the *B. cepacia *complex (Bcc) which are important opportunistic human pathogens. *B. cenocepacia *utilizes a mechanism of cell-cell communication called quorum sensing to control gene expression including genes involved in virulence. The *B. cenocepacia *quorum sensing network includes the CepIR and CciIR regulatory systems.

**Results:**

Global gene expression profiles during growth in stationary phase were generated using microarrays of *B. cenocepacia cepR*, *cciR *and *cepRcciIR *mutants. This is the first time CciR was shown to be a global regulator of quorum sensing gene expression. CepR was primarily responsible for positive regulation of gene expression while CciR generally exerted negative gene regulation. Many of the genes that were regulated by both quorum sensing systems were reciprocally regulated by CepR and CciR. Microarray analysis of the *cepRcciIR *mutant suggested that CepR is positioned upstream of CciR in the quorum sensing hierarchy in *B. cenocepacia*. A comparison of CepIR-regulated genes identified in previous studies and in the current study showed a substantial amount of overlap validating the microarray approach. Several novel quorum sensing-controlled genes were confirmed using qRT-PCR or promoter::*lux *fusions. CepR and CciR inversely regulated flagellar-associated genes, the nematocidal protein AidA and a large gene cluster on Chromosome 3. CepR and CciR also regulated genes required for iron transport, synthesis of extracellular enzymes and surface appendages, resistance to oxidative stress, and phage-related genes.

**Conclusion:**

For the first time, the influence of CciIR on global gene regulation in *B. cenocepacia *has been elucidated. Novel genes under the control of the CepIR and CciIR quorum sensing systems in *B. cenocepacia *have been identified. The two quorum sensing systems exert reciprocal regulation of many genes likely enabling fine-tuned control of quorum sensing gene expression in *B. cenocepacia *strains carrying the cenocepacia island.

## Background

*Burkholderia cenocepacia *is a member of a group of closely related organisms called the *B. cepacia *complex (Bcc), which are important opportunistic pathogens in individuals with cystic fibrosis (CF) or chronic granulomatous disease [1-5]. *B. cenocepacia *and *B. multivorans *are the most common members of the Bcc isolated from lungs of CF patients [6,7]. Infections with *B. cenocepacia *can lead to what is termed "cepacia syndrome", a rapid decline in lung function associated with necrotizing pneumonia, bacteremia and sepsis which can result in death [8]. *B. cenocepacia *is intrinsically resistant to antibiotic therapy and often impossible to eradicate from lungs of infected CF patients [9].

Quorum sensing (QS) is an intricate cell-cell signaling system used by a diverse range of microbial species to communicate with neighbouring cells to regulate gene expression. In Gram-negative bacteria, homologs of the LuxI protein family synthesize signaling molecules termed *N*-acyl-homoserine lactones (AHLs) that are bound by homologs of the LuxR protein family which act as transcriptional regulators (for reviews see [10] and [11]). *B. cenocepacia *has two pairs of QS systems, the CepIR system which is present in all species of the Bcc [12-15] and the CciIR system, which is only present in *B. cenocepacia *containing the *c*eno*c*epacia *i*sland (cci) found in highly transmissible ET12 strains [13]. CepI is primarily responsible for the synthesis of C8-HSL [14] and minor amounts C6-HSL [15]. CciI primarily synthesizes C6-HSL with lesser amounts of C8-HSL produced [16]. At the genomic level, *cepI *and *cepR *are divergently transcribed from each other while *cciI *and *cciR *form a transcriptional unit [16]. The QS systems are arranged in a hierarchical fashion as CepR is required for the transcription of the *cciIR *operon [16]. However, CciR negatively regulates the expression of *cepI *thus allowing negative regulatory feedback on the CepIR system [16]. Additionally, CepR activity can be inhibited by excess amounts of C6-HSL [17]. *B. cenocepacia *also contains a third LuxR homolog, CepR2, that lacks an associated AHL synthase gene [18]. CepR2 negatively regulates its own expression and is negatively regulated by CciR. We have recently identified several CepR2-regulated genes, including virulence factors, and demonstrated that CepR2 can influence gene expression in the absence of AHLs [18].

The CepIR system in *B. cenocepacia *and *B. cepacia *positively influences virulence in murine, nematode, wax moth, alfalfa and onion infection models [19-22]. *B. cenocepacia *CepIR negatively regulates genes involved in the biosynthesis of the siderophore ornibactin [14], but positively regulates expression of the *zmpA *and *zmpB *extracellular zinc metalloprotease genes [14,19,20]. CepIR also positively influences swarming motility [21], biofilm formation and maturation [21,23]. Several studies have shown that CepR positively regulates the expression of AidA, a protein involved in nematode virulence [17,24-26].

Several global approaches have been used to identify the CepIR regulon. A *B. cepacia *ATCC 25416 random promoter library screened in *E. coli *identified 20 ORFs that were positively regulated by CepR in the presence of C8-HSL including a malate synthase gene and oxidative stress induced genes [24]. A random promoter library approach was employed for *B. cenocepacia *K56-2, using a K56-2 *cepI *mutant as a host, that identified 58 or 31 genes with increased or decreased expression, respectively, in the presence of C8-HSL [27], including genes involved in type II and type III secretion systems, catalase activity, cold shock proteins and genes with regulatory functions [27]. Transposon mutagenesis strategies in a *B. cenocepacia cepI *mutant identified seven [17] and six genes [28], differentially regulated by CepR, including the *huv *(*phu*) heme uptake system, a TonB-dependent siderophore receptor, and *aidA *[17,28]. Bioinformatic analysis of known CepR-regulated gene promoters was used to predict a consensus *cep *box motif sequence. An *in silico *screen of the *B. cenocepacia *genome identified promoters containing the *cep *box motif that are potentially regulated by CepIR including genes involved in type V secretion and lipopolysaccharide biosynthesis. CepR was shown to regulate promoters containing a *cep *box upstream of genes now known to encode proteins of the *B. cenocepacia *type VI secretion system (T6SS) [28,29] as well as several other genes including transcriptional regulators [28]. Additionally, a proteomics approach in *B. cenocepacia *H111, which lacks *cciIR*, identified differential expression of 11 proteins, including AidA, FimA, and SodB, when C8-HSL was added to cultures of a H111*cepI *mutant [26]. These combined approaches have facilitated the delineation of genes regulated by the CepIR system; however, there has been relatively little overlap in the genes identified using different approaches which suggests that our knowledge of the CepIR QS regulon is not complete.

Considerably less is known about the genes regulated by the CciIR QS system. A *B. cenocepacia cciI *mutant exhibited reduced virulence in a murine chronic respiratory infection model [13]. Reduced expression of *zmpA *was observed in *B. cenocepacia cciI *and *cciR *mutants [16]. Expression of *zmpB *was shown to be increased and decreased, respectively, in *B. cenocepacia cciI *and *cciR *mutants although a *cciIR *mutant had similar levels to the parent strain [19]. Swarming motility was decreased in a *cciI *mutant but unchanged in a *cciR *mutant [16]. Both the CepIR and CciIR systems regulate *orbI *which is involved in ornibactin synthesis [27].

Studies of expression of individual genes in *cepIR *or *cciIR *mutants have suggested that some genes are co-regulated by these two QS systems, but that other genes are independently regulated. A genome-wide investigation of the contribution of the CciIR system to gene regulation in *B. cenocepacia *has not yet been undertaken. Furthermore, the identification of CepIR-regulated genes is incomplete since differential expression of genes that would account for some observed phenotypes of *cepI *or *cepR *mutants have not yet been reported. In this study, global gene expression profiling was performed using *B. cenocepacia *microarrays in order to more fully comprehend the extent of gene regulation exerted by both CepIR and CciIR. These data provide evidence of the individual, overlapping and opposing roles played by each of the QS systems in the regulation of global gene expression in *B. cenocepacia*.

## Results

### Identification of genes differentially expressed in *cepR*, *cciR *and *cepRcciIR *mutants

**Figure 1:**
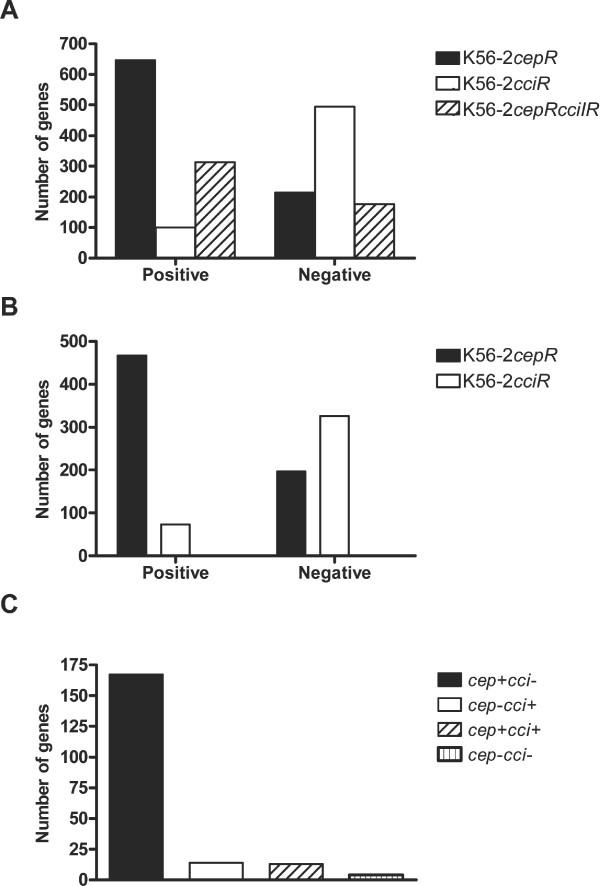
**Genes regulated in QS mutants of *B. cenocepacia *according to microarray analysis**. (A) Total number of genes showing positive or negative regulation in *cepR*, *cciR *or *cepRcciIR *mutants compared to K56-2. (B) Number of genes showing independent regulation in the *cepR *or *cciR *mutants compared to K56-2. (C) Number of genes exhibiting co-regulation were grouped as follows: positive regulation by CepR and negative regulation by CciR (*cep*+*cci*-), negative regulation by CepR and positive regulation by CciR (*cep*-*cci*+), positive regulation by both CepR and CciR (*cep*+*cci*+) and negative regulation by both CepR and CciR (*cep*-*cci*-).

Transcriptional profiling using a custom *B. cenocepacia *oligonucleotide microarray was used to identify genes differentially expressed in stationary phase cultures of strain K56-2 *cepR*, *cciR *and *cepRcciIR *mutants compared to wildtype K56-2. Both CepR and CciR were determined to positively and negatively regulate gene expression, and both QS systems influenced gene expression on all three chromosomes as well as the plasmid (Fig. 1A). Using a 2-fold difference in expression as a cut-off, 646 open reading frames (ORFs) were identified that were positively regulated by CepR and 214 ORFs were identified that were negatively regulated by CepR. CciR positively influenced the expression of 100 ORFs and negatively regulated the expression of 495 ORFs. In the *cepRcciIR *mutant, 313 ORFs exhibited decreased expression and 176 ORFs showed increased expression compared to wildtype (See Additional File 1: Genes with increased or decreased expression in *cepR*, *cciR *or *cepRcciIR *mutants compared to K56-2). Both QS systems regulated genes involved in a range of biological processes including virulence, surface structures, transport or secretion, metabolism and regulation.

Subsets of unique ORFs independently regulated by CepR or CciR were identified in the microarray data (Fig. 1B). CepR was primarily a positive regulator while CciR generally was a negative regulator of gene expression. Although the majority of differentially expressed genes appeared to be independently regulated by either CepR or CciR, 196 ORFs were identified that were regulated by both CepR and CciR (Fig. 1C). Of these 196 ORFs, 167 were positively regulated by CepR and negatively regulated by CciR. Other patterns of co-regulation were observed including negative regulation by CepR and positive regulation by CciR, positive regulation by both QS systems or negative regulation by both QS systems. More than one quarter of the unique ORFs that were reciprocally regulated by CepR and CciR showed the same trend in regulation in the *cepR *and *cepRcciIR *mutants. Other ORFs showed the same regulation pattern in the *cepR *and *cepRcciIR *mutants but no change in the *cciR *mutant. Together, these data indicate that positive regulation is more frequently performed by CepR compared to CciR, that the majority (92%) of co-regulated genes were inversely regulated by CepR and CciR and that CepR regulation can be dominant over CciR regulation.

### CepR gene regulation

To validate the transcriptome data we first compared genes differentially regulated between K56-2 and the *cepR *mutant to data from previous studies on CepRI regulation in this strain. Previously we used a random promoter library to identify promoter::*lux *fusions differentially expressed between K56-2 and a *cepI *mutant [27]. Comparison of transcriptome data of K56-2 and the *cepR *mutant to the promoter fusion data for K56-2 and the *cepI *mutant revealed that there was an overlap of at least 10 genes or operons identified using the two approaches (Table 1). Regulation of these genes by CepR was usually in the same direction and fold change was generally similar between the two studies. In some cases, genes were identified as differentially regulated in the transcriptome analysis that are predicted to be in the same operon as genes previously identified using the promoter fusion approach also suggesting a correlation between the approaches (data not shown). Two genes were identified for which opposite regulation by the CepIR system was observed between the promoter::*lux *fusions and the transcriptome analysis. For example, opposite results between the promoter fusion and microarray data were observed for BCAL1814 expression in the *cepR *mutant (Table 1). Subsequent investigation using qRT-PCR confirmed the microarray data showing reduced BCAL1814 expression in both the *cepR *and *cciR *mutants compared to K56-2 indicating co-regulation (Table 2).

**Table 1:** Comparison of genes (or operons) showing differential expression in microarray analysis and previously determined to be CepIR regulated using transcriptional fusions.

**Gene**	**Function**^a^	**Change (fold) for K56-dI2 (*cepI*) vs K56-2**	**Change (fold) for K56-R2 (*cepR*) vs K56-2**	**Change (fold) for K56-2*cciR *vs K56-2**	**Change (fold) for K56-*2cepRcciIR *vs K56-2**
		
		**Promoter fusion^b^**	**Microarray^c^**	**Microarray^c^**	**Microarray^c^**
BCAL0111	putative TPR domain	-3.7	-2.2	NC	-2.2
BCAL0380	ABC transporter ATP-binding subunit	-2.7	-2.3	NC	NC
BCAL0812	sigma 54 modulation protein	-2.0	-2.8	NC	NC
BCAL1814^d^	MerR family regulatory protein	2.2	-2.9	-2.7	NC
BCAL1990	glucose-6-phosphate isomerase Pgi	-2.2	-2.6	NC	NC
BCAL2244	urocanate hydratase HutU	-2.2	-2.1	NC	NC
BCAL2931^d^	radical SAM superfamily protein	3.2	2.0	NC	NC
BCAL3006^d^	cold shock-like protein CspA	-1.9	-5.6	2.8	-4.9
BCAS0221	ABC transporter ATP-binding protein AfcB (pseudogene)	-1.7	-3.6	2.9	NC
BCAS0409	zinc metalloprotease ZmpA	-2.6	-5.3	4.0	-4.5

^a^Function derived from *B. cenocepacia *J2315 [34].

^c^Change in 16-h cultures of *cepI *mutant compared to 16-h cultures of K56-2 as determined by promoter fusion Subsin et al. 2007 [27].

^c^Change in 16-h cultures of *cepR*, *cciR *or *cepRcciIR *mutants compared to 16-h cultures of K56-2 as determined by microarray analysis with at least two biological replicates (NC, no change).

^d^Presence of a *cep *box motif was identified by Subsin et al. 2007 [27] for this gene.

**Table 2:** Microarray and qRT-PCR analysis of selected genes showing differential expression in *cepR*, *cciR *or *cepRcciIR *mutants compared to K56-2.

**Gene**	**Function**^a^	**Change (fold) for K56-R2 (*cepR*) vs K56-2**		**Change (fold) for K56-2*cciR *vs K56-2**		**Change (fold) for K56-2*cepRcciIR *vs K56-2**	
		
		**microarray^b^**	**qRT-PCR^c^**	**microarray^b^**	**qRT-PCR^c^**	**microarray^b^**	**qRT-PCR^c^**
BCAS0409	zinc metalloprotease ZmpA	-5.3	-5.3	4.0	2.5	-4.5	-3.3
BCAL1814	MerR family regulatory protein	-2.9	-1.4	-2.7	-1.4	NC	2.7
BCAM0189	AraC family regulatory protein	-2.1	-1.3	NC	-2.6	NC	-3.4
BCAM0191	putative non-ribosomal peptide synthetase	-2.3	-7.4	NC	-2.1	NC	-6.8
BCAM0199	outer membrane efflux protein	2.1	1.6	NC	-1.5	NC	1.8
BCAS0293	nematocidal protein AidA	-88.7	-6647	NC	1.7	-214.9	-4632
BCAL0114	flagellin (type II) protein FliC	-2.4	-164.1	2.2	7.7	NC	-127.9
BCAS0225	Shiny variant regulator ShvR	-3.6	-2.6	NC	1.5	-2.3	-2.2
BCAS0220	putative permease	-3.0	-2.5	2.8	1.7	NC	-1.2
BCAS0204	ABC transporter ATP-binding protein	-7.4	-1.7	3.7	4.0	NC	-1.6
BCAM1420	RND family efflux system transporter protein	-4.8	-1.3	10.3	3.0	-7.4	-1.8
BCAM1418	two-component regulatory system, response	NC	-10.7	11.3	3.0	NC	-2.1
BCAM2626	heme receptor protein HuvA	3.4	2.2	-2.2	-2.0	2.7	2.6

^a^Function derived from *B. cenocepacia *J2315 [34].

^b^Change in 16-h cultures of *cepR*, *cciR *or *cepRcciIR *mutants compared to 16-h cultures of K56-2 as determined by microarray analysis with at least two biological replicates (NC, no change).

^c^Change in 16-h cultures of *cepR*, *cciR *or *cepRcciIR *mutants compared to 16-h cultures of K56-2 as determined by qRT-PCR.

In another previous study, we used a bioinformatics approach to search the *B. cenocepacia *J2315 genome with a *cep *box consensus sequence to identify potential CepIR-regulated genes [28]. Twenty-nine ORFs identified to be CepR-regulated by microarray analysis were previously shown to have a *cep *box motif in their predicted promoter region or were located in a putative operon downstream from an ORF with a *cep *box motif (Table 3). These genes included *cepI*, *zmpA*, *aidA*, and genes of the T6SS. Taken together, the data suggest the three different experimental approaches were complementary and that the data obtained by transcriptome analysis are valid since many of the genes identified as CepR-regulated using microarray analysis have been confirmed by other approaches.

**Table 3:** Comparison of genes (or operons) showing differential expression in microarray analysis and identified to have a *cep *box motif.

**Gene**	**Function**^a^	***cep *box motif name or genomic context of downstream gene**^b^	**Change (fold) for K56-R2 (*cepR*) vs K56-2**^c^	**Change (fold) for K56-2*cciR *vs K56-2**^c^	**Change (fold) for K56-*2cepRcciIR *vs K56-2**^c^
BCAL0051	periplasmic solute-binding protein	MST2001	-2.1	2.5	NC
BCAL0052	putative oxidoreductase	downstream from MST2001	-2.2	2.7	NC
BCAL0232	elongation factor Tu	MST2002	-2.1	NC	NC
BCAL0340	putative lipoprotein of T6SS	MST005 & MST2004	-2.8	NC	NC
BCAL0341	putative type VI secretion system protein TssB	downstream from MST005 & MST2004	-2.5	NC	-2.1
BCAL0342	putative type VI secretion system protein TssC	downstream from MST005 & MST2004	-3.1	NC	-2.3
BCAL0343	putative type VI secretion system protein TssD	downstream from MST005 & MST2004	-3.2	2.1	NC
BCAL0344	putative type VI secretion system protein TssE	downstream from MST005 & MST2004	-2.7	NC	NC
BCAL0345	putative type VI secretion system protein TssF	downstream from MST005 & MST2004	-5.1	2.8	-2.1
BCAL0346	putative type VI secretion system protein TssG	downstream from MST005 & MST2004	-2.9	2.1	-2.1
BCAL0347	putative type VI secretion system protein TssH (ClpB)	downstream from MST005 & MST2004	NC	2.1	NC
BCAL0348	putative type VI secretion system protein TssA	downstream from MST005 & MST2004	-2.7	2.1	-2.2
BCAL0831	putative storage protein	MST2009	-5.2	NC	-4.0
BCAL0832	poly-beta-hydroxy-butyrate storage protein PhaA	downstream from MST2009	-2.4	NC	NC
BCAL0833	putative acetoacetyl-CoA reductase PhbB	downstream from MST2009	-2.6	NC	-3.6
BCAL0834	hypothetical protein	downstream from MST2009	-2.4	NC	NC
BCAL0999	sigma-E factor negative regulatory protein 2 RseA2	MST2013	NC	NC	-3.4
BCAL1124	conserved hypothetical protein	MST2014	2.0	NC	NC
BCAL1354	conserved hypothetical protein	MST2020	NC	NC	-2.2
BCAL1562	hypothetical phage protein	MST2024	NC	2.7	NC
BCAL2871	sigma-E factor negative regulatory protein 1 RseA1	MST2036	NC	NC	NC
BCAL2870	sigma-E factor regulatory protein RseB precursor 1 MucB1	downstream from MST2036	NC	NC	-3.5
BCAL3190	IclR family regulatory protein	MST2039	NC	2.3	2.6
BCAL3205^c^	hypothetical protein	MST2040	-2.0	NC	NC
BCAL3419	3-dehydroquinate dehydratase AroQ1	MST2043	-2.0	NC	NC
BCAM1015	putative porin	MST2050	-2.3	NC	NC
BCAM1405	levansucrase	MST2055	-2.9	NC	NC
BCAM1502	hypothetical protein	MST2056	-2.8	NC	NC
BCAM1870	N-acylhomoserine lactone synthase CepI	MST2059	-67.0	NC	-6.9
BCAM1871	hypothetical protein	downstream from MST2059	-37.6	NC	-20.2
BCAM2502	3-dehydroquinate dehydratase AroQ (similar to BCAL3419)	MST2064	-2.1	NC	NC
BCAM2626	putative heme receptor protein HuvA	MST2066	3.4	-2.2	2.7
BCAM2627	putative hemin ABC transport system protein HmuS	downstream from MST2066	3.1	NC	2.9
BCAS0293	nematocidal protein AidA	MST2069	-88.7	NC	-214.9
BCAS0292	hypothetical protein	downstream from MST2069	-137.3	NC	-278.2
BCAS0409	zinc metalloprotease ZmpA	MST2070	-5.3	4.0	-4.5

^a^Function derived from *B. cenocepacia *J2315 [34].

^b^*cep *box motif name from Chambers et al. 2006 [28] or genomic context of downstream.

^c^Change in 16-h cultures of *cepR*, *cciR *or *cepRcciIR *mutants compared to 16-h cultures of K56-2 as determined by microarray analysis with at least two biological replicates (NC, no change).

### Regulation of genes adjacent to *cepI *and *cepR*

The genomic location of *cepI *and the downstream ORF (BCAM1871) suggests they are part of the same operon. Expression of *cepI *and BCAM1871 was reduced in the *cepR *and *cepRcciIR *mutants compared to K56-2 (Tables 3 and 4). BCAM1869 is located between *cepR *and *cepI *and is divergently transcribed from *cepR*. Expression of BCAM1869 was reduced in the *cepR *mutant compared to K56-2 (Table 4). While the function of these proteins is not yet known, genomic locations are conserved for orthologs of BCAM1869 and BCAM1871 in many *B. cenocepacia *and other *Burkholderia *strains [30].

**Table 4:** Microarray analysis of selected genes showing differential expression in *cepR*, *cciR *or *cepRcciIR *mutants compared to K56-2.

**Gene**	**Function**^a^	**Change (fold) for K56-R2 (*cepR*) vs K56-2**	**Change (fold) for K56-2*cciR *vs K56-2**	**Change (fold) for K56-2*cepRcciIR *vs K56-2**
		
		**microarray^b^**	**microarray^b^**	**microarray^b^**
BCAL1369	Sigma factor 70 EcfC (FecI)	4.6	NC	3.0
BCAL1370	Iron uptake regulatory protein FecR	2.7	NC	2.1
BCAL1520	Putative lipoprotein	-2.1	NC	NC
BCAL1528	Flp type pilus assembly protein	-2.1	NC	NC
BCAL1530	Flp type pilus assembly protein	-3.2	NC	-2.0
BCAL1531	Flp type pilus assembly protein	-2.2	NC	NC
BCAL1532	Flp type pilus assembly protein	NC	NC	-2.2
BCAL1533	Putative lipoprotein	-2.1	2.3	NC
BCAL1534	Putative exported protein	-3.3	2.6	NC
BCAL1368	Probable porin	-2.4	2.5	-2.1
BCAL1677	Putative type-1 fimbrial protein	-2.3	3.1	NC
BCAL1688	sigma factor 70 EcfI (OrbS)	NC	-2.5	NC
BCAL1689	MbtH-like protein OrbH	NC	-2.2	NC
BCAL1690	putative dioxygenase OrbG	NC	NC	2.5
BCAL1692	iron transport-related membrane protein OrbD	NC	NC	3.0
BCAL1693	iron transport-related membrane protein OrbF	NC	NC	2.1
BCAL1694	iron transport-related exported protein OrbB	NC	NC	2.4
BCAL1696	ornibactin biosynthesis non-ribosomal peptide synthase OrbI	NC	-2.4	NC
BCAL1697	ornibactin biosynthesis non-ribosomal peptide synthase OrbJ	NC	NC	2.4
BCAL1698	ornibactin biosynthesis protein OrbK	NC	-2.8	NC
BCAL1699	L-ornithine 5-monooxygenase PvdA	NC	-2.8	NC
BCAL1700	ornibactin receptor precursor OrbA	NC	-2.2	2.2
BCAL1701	ornibactin synthetase OrbF	NC	NC	2.2
BCAL1702	ornibactin biosynthesis protein OrbL	NC	NC	2.5
BCAL1722	Putative exported chitinase	-2.8	NC	NC
BCAL2757	Superoxide dismutase SodB	-2.3	NC	NC
BCAL3297	Ferretin DPS-family DNA-binding protein	-2.8	NC	-3.8
BCAL3298	Conserved hypothetical protein	-2.1	NC	-2.8
BCAL3299	Peroxidase/catablase KatB	-2.3	NC	-4.0
BCAM0184	Lectin	NC	2.0	-2.1
BCAM0186	Lectin BclA	-7.5	3.1	-3.2
BCAM0233	ArsR family regulatory protein	NC	-2.0	NC
BCAM0238	Putative ion transporter	NC	2.0	NC
BCAM0239a	*N*-acylhomoserine lactone synthase CciI	NC	52.1	NC
BCAM0240	*N*-acylhomoserine lactone dependent regulatory protein CciR	NC	4.0	-23.5
BCAM0949	Exported lipase LipA	-3.1	NC	NC
BCAM0950	Lipase chaperone LipB	-2.5	NC	NC
BCAM1869	Conserved hypothetical protein	-5.9	NC	NC
BCAM1871	Conserved hypothetical protein	-37.6	NC	-20.2
BCAM2307	Zinc metalloprotease ZmpB	-2.3	2.1	-2.4
pBCA055	Putative membrane protein	-12.0	2.4	-9.1

^a^Function derived from *B. cenocepacia *J2315 [34].

^b^Change in 16-h cultures of *cepR*, *cciR *or *cepRcciIR *mutants compared to 16-h cultures of K56-2 as determined by microarray analysis with at least two biological replicates (NC, no change).

### CciR exerts global gene regulation

The percentage of genes showing differential expression in the *cciR *mutant was 8.3% of the genome compared to 12.0% for the *cepR *mutant (See Additional File 1). The proportion of CciR-regulated genes was similar on each chromosome (7.9 to 8.6%) while 3 genes (3.1%) were regulated on the plasmid indicating CciR regulation is global in *B. cenocepacia*.

A large number of genes independently regulated by CciR were identified (See Additional File 1). CciR negatively regulated expression of 25 unique ORFs encoding ribosomal proteins only two of which were positively regulated by CepR. The majority of these ribosomal proteins form a large cluster from BCAL0233-0261. Expression of ORFs forming part of the putative AmrA-AmrB-BCAL1676 efflux pump was increased in the *cciR *mutant compared to K56-2. Expression of some of the genes encoding for components of this pump was also increased during growth of *B. cenocepacia *in CF sputum [31].

CciR reciprocally regulated many genes previously reported to be CepR-regulated [27,28] (Tables 1 &3). Examples include increased expression of the cold shock-like protein CspA and the majority of genes in the BCAL0340-0348 T6SS operon in the *cciR *mutant compared to the *cepR *mutant. Additionally, CciR negatively regulated expression of pBCA055 (*bqiC*) which encodes a hypothetical protein with GGDEF and EAL domains (Table 4). Weingart et al. [17] reported CepR positively regulated expression of pBCA055 which was confirmed in the *cepR *and *cepRcciIR *mutants (Table 4).

### Regulation of genes on the cenocepacia island

The *cciIR *genes are located on a genomic island only found in strains of the ET12 lineage [13]. Mutations in two additional genes located on this island, *amiI *(BCAM0265) and *opcI *(BCAM0267), were found to reduce virulence in respiratory infections [13]. It was of interest to determine if CciR regulated these genes or others present on the genomic island. CciR did not influence expression of *amiI *or *opcI*; however it did influence expression of several other genes on the island (See Additional File 1). CciR positively regulated BCAM0233, the first gene in an arsenic resistance operon, and negatively regulated a putative ion transporter (BCAM0238) (Table 4).

Microarray analysis showed CciR negatively regulated its own expression, and that of *cciI*, as has previously been reported using *cciR*::*lux *promoter fusions [16]. Although the *cciI *and *cciR *genes were shown to be co-transcribed [16], *cciI *expression was markedly more increased than the expression of *cciR *in the *cciR *mutant (Table 4). Microarray analysis showed *cciR *expression was decreased in the *cepRcciIR *mutant compared to K56-2 (Table 4). Expression of neither *cciI *nor *cciR *was changed in the *cepR *mutant grown in LB although we have previously demonstrated that CepR was required for *cciIR *expression in PTSB medium using *cciR*::*lux *promoter fusions [16].

### Regulation of genes encoding extracellular enzymes by CepR and CciR

We have previously shown decreased expression of *zmpA *in the *cepR *mutant compared to K56-2 [20,27]. Microarray and qRT-PCR analysis confirmed that *zmpA *expression was reduced in the *cepR *mutant (Tables 1 &2) and demonstrated that *zmpA *expression was reduced in the *cepRcciIR *mutant (Tables 1 &2) confirming phenotypic data for these mutants [16]. Decreased expression of *zmpA *in the *cciR *mutant compared to K56-2 was previously demonstrated in PTSB medium using promoter::*lacZ *fusions [16]. In the current study, *zmpA *expression was increased in the *cciR *mutant compared to K56-2 in cultures grown in LB medium for 16 h, by both microarray and qRT-PCR analysis (Tables 1 &2).

Expression of *zmpB *was previously shown to be decreased in *cepR*, *cciR *and *cepRcciIR *mutants but increased in a *cciI *mutant compared to K56-2 using promoter::*lux *fusions [19]. Positive regulation of *zmpB*, was confirmed in the *cepR *and *cepRcciIR *mutants by microarray analysis (Table 4); however, *zmpB *expression was increased in the *cciR *mutant compared to K56-2. Although LB medium was used in both cases, data from the promoter::*lux *fusions was generated at 20 h growth while data from the microarray analysis was performed on cultures grown for 16 h. Several attempts were made to quantitate *zmpB *expression levels by qRT-PCR but a high degree of variability was observed for this weakly expressed transcript. In fact, *zmpB *was the most weakly expressed transcript in the majority of the microarray samples. Together, these data indicate *zmpA *and *zmpB *expression is positively influenced by CepR under all conditions examined and that growth medium and phase of growth influence regulation by CciR.

Genes encoding the exported lipase LipA (BCAM0949) and the lipase chaperone LipB (BCAM0950, previously called *limA*) are required for lipase production [32]. Expression of both *lipA *and *lipB *was decreased in the *cepR *mutant compared to K56-2, but was unchanged in the *cciR *or *cepRcciIR *mutants (Table 4). Lipase activity was previously shown to be reduced in a *cepR *mutant compared to K56-2 [14].

BCAL1722 encoding a putative exported chitinase showed decreased expression in the *cepR *mutant compared to K56-2 but no change in expression in the *cciR *or *cepRcciIR *mutants (Table 4). Chitinase activity has been shown to be CepIR-regulated in *B. cenocepacia *H111, with lower activity reported in *B. cenocepacia *H111 *cepI *and *cepR *mutants [21].

### CepR and CciR regulate genes adjacent to *cepR2*

We have recently shown that the *B. cenocepacia *orphan LuxR homolog CepR2 is involved in negative regulation of genes adjacent to itself [18]. Additionally, *cepR2 *expression is increased in the *cciR *mutant [18]. Several CepR2-regulated genes and operons also showed differential expression in the current study (See Additional File 1). These genes included BCAM0189 (AraC family regulatory protein), BCAM0191 (putative non-ribosomal peptide synthetase) and BCAM0199 (outer membrane efflux protein). Expression of BCAM0189 and BCAM0191 were decreased but expression of BCAM0199 was increased in the *cepR *mutant compared to K56-2 (Table 2). This trend in regulation was confirmed for all three genes using qRT-PCR (Table 2). BCAM0189, BCAM0191 and BCAM0199 expression levels were similar between the *cciR *and *cepRcciIR *mutants compared to K56-2 by microarray; however, qRT-PCR analysis indicated that expression of these genes was decreased in the *cciR *mutant, but the expression pattern was similar between the *cepRcciIR *and *cepR *mutants (Table 2). CepR2 negatively regulates expression of the lectin-encoding gene, *bclA*, [33] which lies adjacent to two other co-transcribed lectin-encoding genes (BCAM0185-0184) [18]. Expression of *bclA *was decreased in the *cepR *mutant (Table 4). Expression of both *bclA *and BCAM0184 was increased in the *cciR *mutant and decreased in the *cepRcciIR *mutant compared to K56-2 (Table 4).

### CciR negatively regulates *aidA *expression

**Figure 2:**
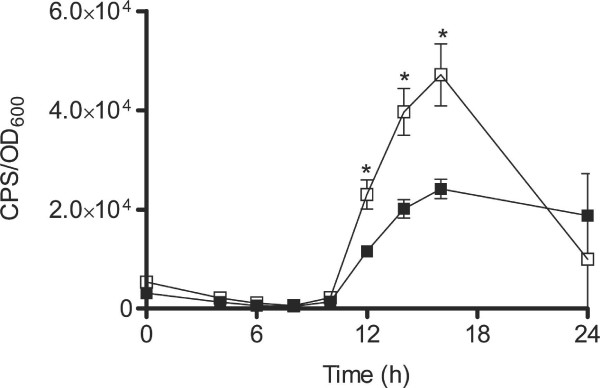
**Expression of *aidA *in the *cciIR *mutant compared to K56-2**. Expression was monitored throughout growth in PTSB plus 100 μg/ml of Tp. The expression of *aidA::lux *(pAidA) is significantly greater in K56-2*cciIR *than K56-2 from 12 to 16 h along the time course (*, *P *< 0.05, unpaired *t*-test, Welch corrected). All values are the means ± SD of triplicate cultures and are representative of three individual trials: **Black Square**:, K56-2; **White Square**: K56-2*cciIR*.

Several studies illustrated that CepR positively regulates the expression of the nematocidal protein, AidA [17,24-26]. Expression levels of *aidA *and BCAS0292 were reduced in the *cepR *and *cepRcciIR *mutants (Table 2). Measurement of *aidA *expression using qRT-PCR confirmed reduced expression in the *cepR *and *cepRcciIR *mutants and indicated increased expression in the *cciR *mutant compared to K56-2 (Table 2). Negative regulation of *aidA *expression by CciR was also demonstrated using a promoter::*lux *fusion which showed significantly increased *aidA *expression in the *cciIR *mutant between 12 and 16 h of growth (*P *< 0.05, unpaired *t*-test, Welch corrected) (Fig. 2).

### QS regulation of flagellar and motility genes

**Figure 3:**
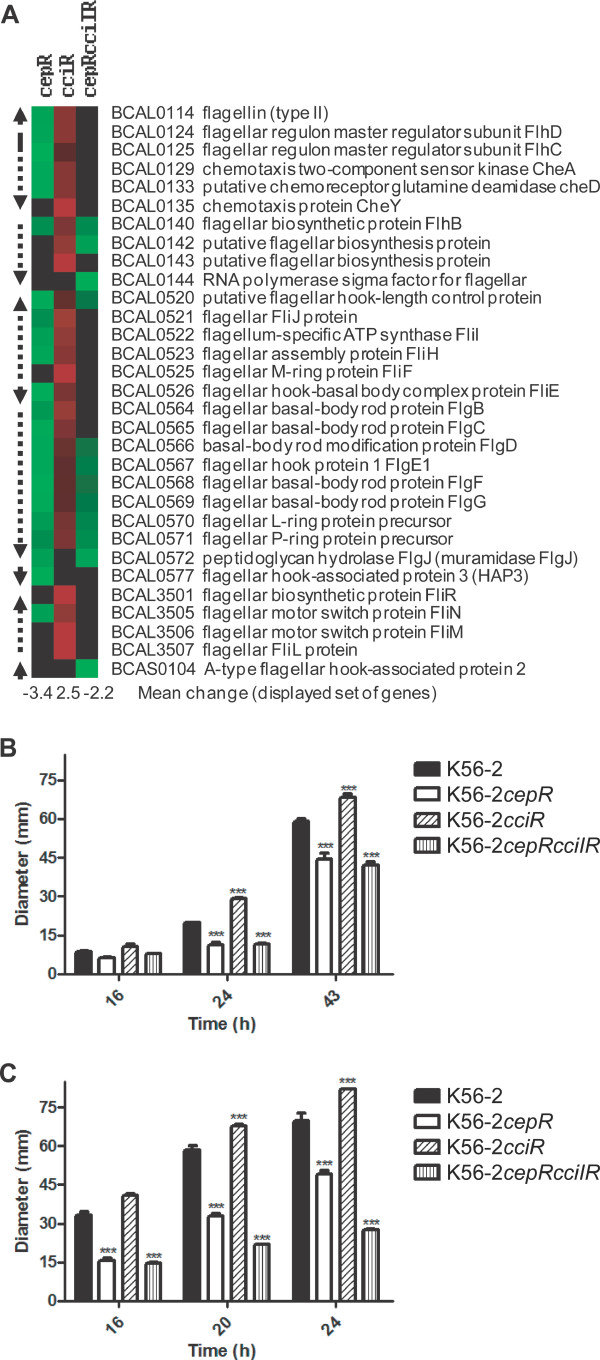
**Regulation of flagellar-associated genes and swimming motility**. (A) Cluster analysis of flagellar-associated genes with decreased (green), increased (red) or no change (black) in expression in each mutant compared to K56-2 according to microarray analysis. Arrows indicate putative (dashed lines) or experimentally-determined (solid lines) transcriptional units. Gene name and function are derived from *B. cenocepacia *[34]. Mean change (fold) in expression is indicated for the displayed group of genes for each mutant. Swimming motility was assessed by measuring zones of growth of cultures at (B) 22°C or (C) 28°C. Significantly different swimming motilities were observed for the QS mutants compared to K56-2 at 22°C and 28°C (***, *P *< 0.001, two-way ANOVA) (Fig. 3). All values are the means ± SEM of triplicate cultures and are representative of two individual trials.

Previously it was demonstrated that the *cepR *mutant exhibits reduced swarming motility while the *cciR *mutant has similar swarming motility compared to K56-2 [16]. Expression levels of 31 genes in nine operons involved in flagellar motility were analyzed. The overall trend showed decreased expression of these genes in the *cepR *and *cepRcciIR *mutants but increased expression in *cciR *mutant compared to K56-2 (Fig. 3A). Investigation of *fliC *(BCAL0114) expression using qRT-PCR confirmed this trend in regulation (Table 2). Separately, activity of a *fliC *promoter::*lux *fusion confirmed *fliC *expression was positively regulated by CepR and negatively regulated by CciR (data not shown). No obvious difference in flagellin protein expression was detected by Western blot in the QS mutants compared to wildtype (data not shown). Mutations in *cepI *or *cepR *were previously reported not to alter swimming motility of *B. cenocepacia *H111 growing at 37°C [21]. Strain K56-2*cepR *and *cepRcciIR *mutants exhibited significantly reduced swimming motility at 22°C and 28°C compared to K56-2 (*P *< 0.001, two-way ANOVA) (Fig. 3BC), whereas swimming motility was significantly increased in the *cciR *mutant at 22°C and 28°C compared to K56-2 (*P *< 0.001, two-way ANOVA) (Fig. 3BC).

### Flp type pilus and fimbrial proteins

Bacterial pili and fimbriae are frequently involved in binding eukaryotic cells and have been shown to play a role in infection in many pathogenic bacteria. A flp type pilus cluster containing two putative operons from BCAL1524-1520 and BCAL1525-1537 has been identified [34]. Several genes in these operons showed decreased expression in the *cepR *and *cepRcciIR *mutants while two genes showed increased expression in the *cciR *mutant (Table 4) suggesting reciprocal regulation of this cluster. It is likely that the BCAL1525 operon is similar to that of clone P15 which was positively regulated by CepR in *B. cepacia *[24]. BCAL1677, which was positively regulated by CepR and negatively regulated by CciR (Table 4), encodes a putative type-1 fimbrial protein and was shown to be CepIR-regulated in *B. cenocepacia *H111 [26].

### QS controls a regulator involved in virulence (ShvR) and proteins of unknown function

**Figure 4:**
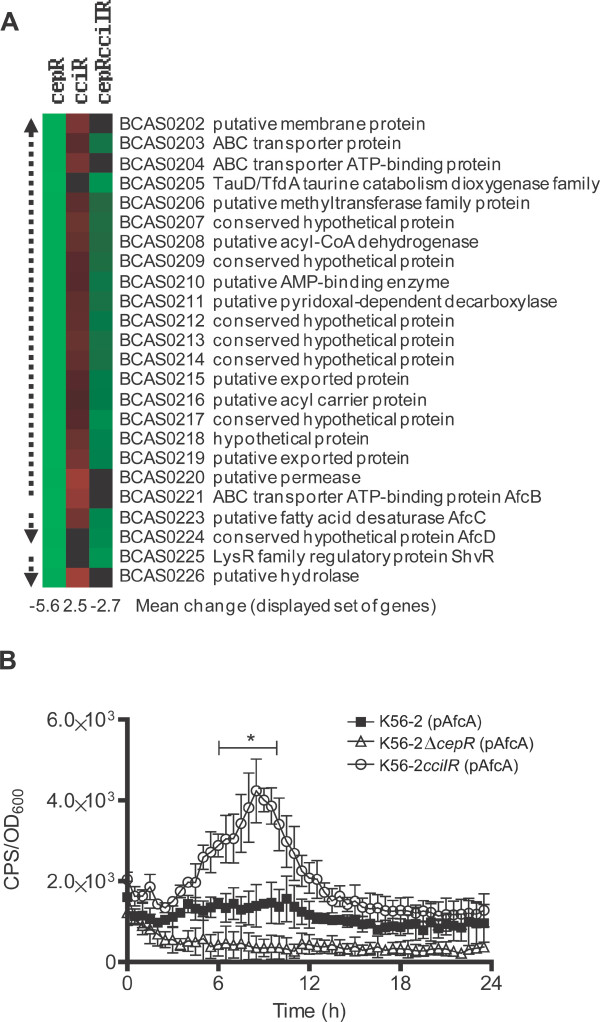
**Regulation of a 27 kb gene cluster on Chromosome 3**. (A) Cluster analysis of genes in the *afcA *and *shvR *genomic regions with decreased (green), increased (red) or no change (black) in expression in each mutant compared to K56-2 according to microarray analysis. Arrows indicated putative (dashed lines) or experimentally-determined (solid lines) transcriptional units. Gene name and function are derived from *B. cenocepacia *[34]. Mean change (fold) in expression is indicated for the displayed group of genes for each mutant. (B) Expression of *afcA *was monitored throughout growth in K56-2Δ*cepR *and K56-2*cciIR *mutants compared to K56-2 in PTSB plus 100 μg/ml of Tp. The expression of *afcA::lux *(pAfcA) is significantly greater in K56-2*cciIR *than K56-2 from 6 to 10 h along the time course (*P *< 0.05, unpaired *t*-test, Welch corrected). All values are the means ± SD of triplicate cultures and are representative of two individual trials.

Many ORFs located in a genomic region of approximately 27 kb on Chromosome 3 were differentially expressed in the QS mutants compared to K56-2 (Fig. 4A). Part of this QS-regulated gene cluster included BCAS0225, which regulates the rough-shiny morphotype and contributes to virulence in K56-2 [35]. Expression of BCAS0225 (which we now refer to as *shvR *for *sh*iny *v*ariant *r*egulator) was decreased in the *cepR *and *cepRcciIR *mutants compared to K56-2 (Table 2). Lower expression of *shvR *was confirmed using qRT-PCR in the *cepR *and *cepRcciIR *mutants while increased expression was observed in the *cciR *mutant (Table 2).

In *B. cepacia *BC11, AfcA and AfcCD are responsible for production of an antifungal compound [36]. Orthologs of these genes are present in *B. cenocepacia *as part of two putative transcriptional units; *afcA *(BCAS0222) to BCAS0202 and *afcCD *[30]. In the *afcA *genomic region, expression of the majority of ORFs was decreased in the *cepR *and *cepRcciIR *mutants but increased in the *cciR *mutant compared to K56-2 (Fig. 4A). Furthermore, qRT-PCR analysis of BCAS0204 and BCAS0220 confirmed this trend in the *cepR*, *cciR *and *cepRcciIR *mutants (Table 2). Although a difference in *afcA *expression was not detectable in these mutants by microarray analysis, an *afcA*::*lux *promoter fusion had lower expression in the *cepR *mutant and significantly higher expression in the *cciIR *mutant compared to K56-2 between 6 and 10 h of growth (*P *< 0.05, unpaired *t*-test, Welch corrected) (Fig. 4B). Previously, CepR was also shown to positively regulate an *afcB*::*lux *reporter fusion [27] (Table 1).

### Transcriptional control of a resistance-nodulation division family efflux pump

*B. cenocepacia *is known to exhibit high levels of intrinsic resistance to antimicrobials [9]. At least 14 potential resistance-nodulation division (RND) family efflux pumps [37] have been identified in *B. cenocepacia *[37,38]. We recently demonstrated CepR2 positively regulates expression of BCAM1420, part of a putative RND efflux pump [18]. Expression of BCAM1420 and several adjacent regulatory genes was lower in the *cepR *and *cepRcciIR *mutants but increased in the *cciR *mutant compared to K56-2 (Table 2) (See Additional File 1). The expression patterns of BCAM1420 and BCAM1418 (two-component regulatory system, response regulator) were confirmed by qRT-PCR (Table 2). Antibiotic susceptibility is not significantly different in peg-formed biofilms of *cepR *or *cciR *mutants compared to K56-2 [23]. No differences in resistance to a selection of heavy metals were observed between the QS mutants and K56-2 (data not shown).

### Reciprocal QS regulation of iron transport genes

We have shown CepR negatively regulates ornibactin synthesis in *B. cenocepacia *strains K56-2 and H111 [15,18]. No change in expression was detected for any gene in the *orbI *(BCAL1696) or *ecfI *(*orbS*, BCAL1688) operons in the *cepR *mutant (Table 4); however, expression of several genes in these operons was decreased in the *cciR *mutant and increased in the *cepRcciIR *mutant compared to K56-2 (Table 4). This indicates that CciR positively regulates ornibactin synthesis and transport genes, and suggests CepIR is dominant over CciIR, since these genes are negatively regulated in the *cepRcciIR *mutant as has previously been reported in the *cepR *mutant.

*B. cenocepacia *contains a FecIR-like system potentially involved in iron uptake [39]. Increased expression of *fecI *(*ecfC*, BCAL1369) and *fecR *(BCAL1370) was detected in *cepR *and *cepRcciIR *mutants compared to K56-2 indicating that CepR also negatively regulates ferric citrate transport (Table 4). Divergently transcribed from *efcC *is a probable porin gene that showed lower expression in *cepR *and *cepRcciIR *mutants compared to K56-2 (Table 4). This appears to be similar to clone P57 identified by Aguilar et al. 2003 [24] that was positively regulated by CepR in *B. cepacia*. Expression of a putative oxidoreductase (BCAL0269) and a ferric reductase-like transmembrane component (BCAL0270) was decreased in the *cepRcciIR *mutant compared to K56-2 (Table 4). It was recently reported that expression of BCAL0270, was increased during growth of *B. cenocepacia *in CF sputum [31].

We previously demonstrated that genes involved in heme transport are positively regulated by the CepIR QS system and that *huvA *(BCAM2626) contained a *cep *box in its promoter region [28] (Table 3). In this study *huvA *expression was increased in the *cepR *and *cepRcciIR *mutants and decreased in the *cciR *mutant compared to wildtype. This trend in regulation for *huvA*, which contradicts the data obtained previously with *huvA *transcriptional fusions was independently confirmed using qRT-PCR (Table 2).

### CepR regulation of oxidative stress genes

*B. cenocepacia *contains a major catalase/peroxidase protein, KatB (BCAL3299), important for resistance to hydrogen peroxide [40]. Expression of a putative three-gene operon consisting of *katB *and two downstream ORFs was decreased in the *cepR *and *cepRcciIR *mutants compared to K56-2 (Table 4). Expression of superoxide dismutase *sodB *(BCAL2757), previously shown to be CepR-regulated in *B. cenocepacia *H111 [26], was also reduced in the *cepR *mutant compared to K56-2 (Table 4).

### QS regulation of phage-related genes

Recently, an epidemic strain of *Pseudomonas aeruginosa *with mutations in prophage and genomic island sequences was shown to have reduced ability to compete with the parent strain in a rat lung infection model [41]. *B. cenocepacia *J2315 possesses 5 prophages contained on 14 genomic islands (termed BcenGI) [34]. Twenty-four differentially regulated phage-related genes were identified in the *cepR *mutant, twenty genes were differentially expressed in the *cepRcciIR *mutant, whereas only 5 phage-related genes showed changes in expression in the *cciR *mutant (See Additional File 1).

## Discussion

In this study we characterized the contributions of CepR and CciR to global gene regulation in *B. cenocepacia*. Elucidation of the CciR regulon indicates that it is a global regulator of QS gene expression in *B. cenocepacia*. Many CepR-regulated genes identified in prior studies were also identified as CepR-regulated using microarrays. This approach facilitated the identification of novel genes regulated by the CepIR and CciIR QS systems. Genes independently regulated by CepR or CciR, as well as co-regulated genes, were identified. Importantly, the majority of co-regulated genes were reciprocally regulated by CepR and CciR. This pattern of regulation was independently confirmed for a number of these genes using qRT-PCR or promoter::*lux *fusions.

CepR-regulation of AidA has been consistently identified in previous studies [17,24-26] suggesting tight regulation by CepR. The putative two-gene operon comprising *aidA *and the downstream ORF (BCAS0292) contained the most highly regulated genes in the *cepR *and *cepRcciIR *mutants compared to K56-2. Promoter::*lux *fusions showed that *aidA *expression was negatively regulated by the CciIR system. Other genes, including *cepI*, *cciI*, pBCA055 and BCAM1871 also showed high levels of QS regulation (12- to 67-fold) using microarrays. The majority of genes showed low levels of QS regulation consistent with previous studies in *B. cenocepacia *K56-2 [27,28]. A 2-fold change in expression was selected as a cut-off for microarray analysis which was consistent with the degree of differential expression of a number of previously-identified CepR-regulated genes in strain K56-2 [17,27,28]. These changes in gene expression have also been correlated with changes in some phenotypes such as protease activity and siderophore production [15,19,20]. Furthermore, some CepR-regulated genes identified in *B. cenocepacia *H111 or *B. cepacia *ATCC 25416 were shown to be CepR regulated in strain K56-2, suggesting that QS-mediated regulation of many genes is conserved in the Bcc [24,26].

The interrelationship between the CepIR and CciIR systems is complex. Although we previously reported that CciR negatively regulated *cepI *in PTSB medium [16], no change in *cepI *expression was detectable in the transcriptome analysis of the *cciR *mutant grown in LB medium; however, consistent with previous data, CciR negatively regulated expression of the *cciIR *operon. We previously demonstrated that CepR was required for expression of a *cciIR *promoter::*lux *fusion in PTSB medium [16], but similar results were not obtained in this study with cultures grown in LB medium. These data suggest that the regulatory relationship between the two QS systems varies depending on growth conditions and nutrient availability.

Presence of cci has been associated with several transmissible *B. cenocepacia *strains [13]. The cci can be found in strains belonging to the ET12 lineage [13], but is absent from genomes of other *B. cenocepacia *strains, including representatives of the transmissible PHDC lineage (AU1054, HI2424) and strain H111 [26,34]. Our study now shows that presence of CciIR in certain *B. cenocepacia *strains has major implications for QS-regulated genes across the genome; most notably genes reciprocally regulated by CepIR and CciIR. Three master regulators of CepR have been identified in *B. cenocepacia *H111 [42]. It is unknown if other regulatory factors (other than CciR, SuhB, YciL and YciR) exist which can limit the influence of CepR and thus provide balance to QS-mediated gene expression. This may have important consequences during infection to fine tune expression of virulence factors in response to particular environmental cues.

It has previously been shown that LasR, RhlR and QscR, components of the *Pseudomonas aeruginosa *QS network, have overlapping but distinct regulons [43]. The presence of *las*-*rhl *box-like sequences in the promoter regions of many QS-regulated genes has been demonstrated [44]. A subsequent study showed a consensus motif could be more easily identified for genes regulated by RhlR rather than LasR and that a single conserved motif could not be identified for QS-regulated promoters [45]. Recently, it was shown that *rhlR *is expressed in the absence of LasR in *P. aeruginosa *[46] and that RhlR exerts regulation over genes that were thought to be specifically LasR-regulated including *lasI*.

In our study we confirmed CepR regulation for a number of genes which contain a *cep *box motif in their promoters [28]. We do not know if CepR and CciR bind the same promoter motif. There were no genes with a *cep *box in their promoters that showed only CciR regulation. Negative regulation by CciR occurs in genes with or without a *cep *box in their promoters suggesting this motif is not required for regulation by CciR and opens the possibility that CciR recognizes a distinct motif. Gene regulation in the *cepRcciIR *mutant more closely resembled gene regulation in the *cepR *mutant than the *cciR *mutant. This suggests CepR is the more dominant regulator and very likely acts upstream from CciR. Direct binding of CepR to two promoters has been demonstrated [17]. CepR and CciR promoter binding studies might provide insight into the reciprocal regulation of co-regulated genes. It is possible that CciR could inactivate CepR by forming heterodimers as has been suggested as a mechanism for negative regulation by QscR [47]. The majority of C8-HSL or C6-HSL is produced by CepI and CciI, respectively [14-16]. Therefore, QS-regulation by CepR and CciR may also be strictly dependent on the relative abundance of the signaling molecules.

Flagella are recognized as important virulence factors for human and plant pathogens [48]. A clear change in expression was observed at the transcriptional level for many flagellar-associated genes in the QS mutants compared to wildtype. Differences in swimming motility were found when cultures were grown at 22°C and 28°C confirming the reciprocal regulation of flagellar-associated genes and swimming motility by the CepIR and CciIR systems. A change in flagellin protein expression was not apparent in the QS mutants grown at 37°C. Positive control of flagella formation by QS is observed in *B. glumae*. However, lack of flagella formation in a *B. glumae tofI *mutant can be overcome by incubation at 28°C indicating other factors are involved [49]. Reduced biosurfactant production, as opposed to improper flagella formation, is responsible for reduced swarming in an AHL-deficient mutant of *Serratia liquefaciens *[50]. Reduced swarming observed in the *B. cenocepacia *H111*cepR *mutant can be restored by addition of exogenous surfactants. An endogenously-produced biosurfactant has not yet been described in *B. cenocepacia*. From an evolutionary perspective, flagella share common features with the type 3 secretion system (T3SS) [51] which we have also shown to be positively regulated by CepIR [27]. The expression of flagellar-associated and T3SS genes was increased in *B. cenocepacia *grown in CF sputum indicating conditions promoting their expression may be present during infection [31].

The majority of genes in the *afcA *genomic region have not been studied in detail in *Burkholderia *species, although some of these genes in *B. cepacia *BC11 are responsible for production of an antifungal with inhibitory activity against *Rhizoctonia solani *[36]. Antifungal production was highest in stationary phase and under low aeration growth conditions [36]. Expression of three putative operons containing *afcA*-BCAS0202, *afcCD *and *shvR*-BCAS0226 is induced in a *B. cenocepacia *agricultural field isolate (strain HI2424) growing under soil-like conditions compared to CF-like conditions but was unchanged in a *B. cenocepacia *CF isolate (strain AU1054) in these conditions [52]. We have now clearly shown that both CepR and CciR regulate many genes in the *afcA *genomic region.

Our recent characterization of the *B. cenocepacia *orphan LuxR homolog CepR2 [18] facilitated the incorporation of its regulon into the current study. A number of CepR2-regulated genes were also regulated by CepR and CciR, including genes in the genomic region adjacent to *cepR2*. A larger number of co-regulated genes may have been identified if the experiments were conducted at the same time in growth as opposed when CepR2 expression is maximal (mid-log phase) and when CepR and CciR expression is maximal (late stationary phase).

Intrinsic antibiotic resistance due to efflux pumps is a major problem associated with the treatment of *B. cenocepacia *infections. Presence of multiple efflux pumps with redundant functions is suggested by the lack of difference in *cepR *or *cciR *mutants compared to wildtype in resistance to antibiotics and heavy metals [23] (this study). However, QS regulation of efflux pumps may have other consequences. Efflux pumps are also involved in the efflux of QS molecules in *P. aeruginosa *[53] and *B. pseudomallei *[54]. QS regulation of efflux pumps may be more important in certain environments than in others. Growth of *B. cenocepacia *in CF sputum was shown to increase expression of a component of the BCAM0199-0201 putative multi-drug efflux pump [31] which we showed was negatively regulated by CepR and positively regulated by CciR and CepR2 [18] (this study).

Iron is an essential cofactor in many metabolic pathways however iron availability is usually limited in the host [55]. *B. cenocepacia *produce the siderophores ornibactin and pyochelin to sequester iron. CepR2 and indirectly CepR positively regulate pyochelin biosynthesis in *B. cenocepacia *H111 [18]. Pyochelin biosynthesis does not occur in *B. cenocepacia *K56-2 because a point mutation exists in the pyochelin synthetase gene *pchF *[34]. In this study we demonstrate that the CepR and CciR systems inversely regulate genes involved in ornibactin biosythesis and uptake, as well as regulate genes involved in other iron transport systems including heme and the FecIR uptake systems. QS regulation of multiple iron transport mechanisms may facilitate growth in specific environmental niches where resources are limited. Some ornibactin genes were poorly expressed, most likely due to the fact cultures for microarray analysis were grown in LB medium compared to cultures with promoter::*lux *fusions which were grown in low-iron TSB-DC medium [56]. The difference in media may explain the difference in regulation for some of these iron acquisition genes between the current and previous studies [28], since we also demonstrated that the expression of *cepI *and *cciIR *varied depending on the media. Different media are also known to influence *lasIR*, *rhlIR *and QS-regulated genes in *P. aeruginosa *[57].

Evidence of a link between oxidative stress and QS was shown in the regulation of *katA*, *sodA *and *sodB *by the *las *and *rhl *systems in *P. aeruginosa *[58]. In *B. pseudomallei*, the response to oxidative stress occurs through QS regulation of *dpsA *expression [59]. In this study we demonstrated that CepR regulates *katB *(BCAL3299) and a downstream gene BCAL3297 encoding a putative ferritin DPS-family DNA binding protein in *B. cenocepacia*. Genomic analysis suggests that BCAL3297 is a homolog of the ORF designated *dpsA *positioned downstream from *katG *(clone P80), which is CepR-regulated in *B. cepacia *[24].

QS-regulated phage-related genes included a large proportion of genes contained on prophage BcenGI12. We have not investigated the consequences of QS regulation on phage activity in *B. cenocepacia*; however, mutation of phage components in *P. aeruginosa *has important consequences for bacterial competition in vivo [41]. It is enticing to consider that unidentified virulence factors may be carried on genomic islands/prophages and that expression of these is under QS control. It is also possible that cell density-dependent regulation of phage elements facilitates genomic rearrangement within a species or horizontal gene transfer between mixed bacterial populations.

## Conclusion

**Figure 5:**
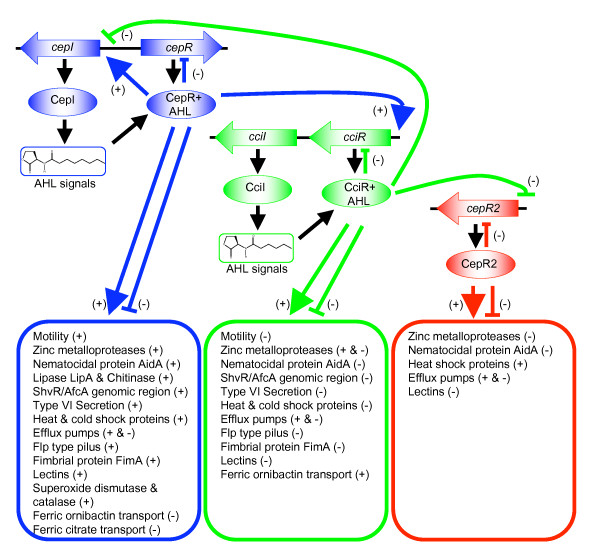
**Hierarchical organization of the CepIR, CciIR and CepR2 quorum sensing systems and traits under their control in *B. cenocepacia *K56-2**. Summary diagram of the regulatory interrelationship between CepIR and CciIR, and CciIR and CepR2. AHLs are required to activate CepR and CciR but not CepR2. All three regulators negatively control their own expression. The three QS systems positively and negatively influence gene expression. +, positive regulation; -, negative regulation.

The CepIR and CciIR QS systems regulate expression of multiple genes at the transcriptional level in *B. cenocepacia*, including potential virulence genes. QS-regulated genes involved in motility, biofilm formation/adhesion, extracellular enzymes, secretion systems, iron transport, stress response and antibiotic resistance are summarized in Fig. 5. The CepIR system is primarily responsible for positive regulation while the opposite is true for the CciIR system. The majority of the co-regulated QS-controlled genes are subject to reciprocal regulation by CepR and CciR. Until now the scale of this inverse regulation was not fully appreciated. The antagonistic influence of CepR and CciR ensures that QS-regulated gene expression in *B. cenocepacia *is tightly regulated. Novel gene clusters, not previously shown to be QS-regulated, were identified, facilitating the future examination of these genes in relation to pathogenesis. This work provides significant advances for understanding QS-mediated regulation of virulence genes in *B. cenocepacia*. A detailed picture of the QS network is required to facilitate the development of therapies aimed at interfering with cell-cell communication systems to control bacterial infection.

## Methods

### Strains and growth conditions

The bacterial strains used in this study are listed in Table 5. Cultures were routinely grown at 37°C, in Miller's Luria broth (LB) (Invitrogen, Burlington, ON) with shaking or on 1.5% Lennox LB agar plates. For promoter::*lux *assays, strains were grown in 0.25% trypticase soy broth (Difco, Franklin Lakes, NJ) with 5% Bacto-Peptone (Difco) (PTSB). Swimming motility assays were performed as previously described [35] except that overnight cultures were normalized to an OD_600 _of 0.4 prior to inoculation and growth was assessed at 22°C or 28°C. When appropriate, the following concentrations of antibiotics were used: 100 μg/ml of trimethoprim (Tp) and 200 μg/ml of tetracycline (Tc). Antibiotics were purchased from Sigma-Aldrich Canada Ltd. (Oakville, ON).

**Table 5:** Bacterial strains and plasmids used in this study.

**Strain or plasmid**	**Description**	**Reference**
Strains		

*B. cenocepacia*		
K56-2	CF isolate, BCESM +,	[70]
K56-R2	*cepR*::Tn5-OT182 derivative of K56-2, Tc^R^	[14]
K56-2Δ*cepR*	Δ*cepR *derivative of K56-2	This study
K56-2*cciR*	*cciR*::Tp derivative of K56-2, Tp^R^	[16]
K56-2*cciIR*	Δ*cciIR *derivative of K56-2	[16]
K56-2*cepRcciIR*	*cepR*::Tp, Δ*cciIR *derivative of K56-2, Tp^R^	[16]
*E. coli*		
DH5α	F-*mcr*A Δ(*mrr-hsd*RMS-*mcr*BC) φ80*lac*ZΔM15 Δ*lac*X74 *rec*A1 *end*A1 *ara*D139Δ(*ara, leu*)7697 *gal*U *gal*K λ-*rps*L *nup*G	Invitrogen
TOP10	F^-^*mcr*A Δ(*mrr*-*hsd*RMS-*mcr*BC) φ80*lac*ZΔM15 Δ*lac*X74 *rec*A1 *ara*D139Δ(*ara*-*leu*)7697 *gal*U *gal*K *rps*L (Str^R^) *end*A1 *nup*G λ-	Invitrogen
SY327	*araD*, Δ*(lac pro) argE(Am) recA56 rif*^R^*nalA*λ*pir*	[71]
Plasmids		

pGPI-SceI	*ori*_R6K_, Tp^R^, *mob*^+^, carries I-SceI cut site	[65]
pDAI-SceI	pDA17 carrying the I-SceI gene	[65]
TPCR2.1::H12	pCR2.1TOPO containing amplified homologous regions flanking *cepR*	This study
pGPI-SceI::H12	Mutageneis plasmid pGPI-SceI with homologous regions flanking *cepR*	This study
pAidA301	*aidA::lux *transcriptional fusion constructed in pMS402 Km^R ^Tp^R^	[28]
pAfcA	*afcA::lux *transcriptional fusion constructed in pMS402 Km^R ^Tp^R^	This study

### DNA manipulations

DNA manipulations were performed using standard techniques as described by Sambrook et al. [60]. Genomic DNA was isolated as described by Ausubel et al. [61] or Walsh et al. [62]. Oligonucleotide primers (See Additional File 2: Oligonucleotide primers used in this study) were designed with Primer3 [63] and were synthesized by the University of Calgary Core DNA and Protein Services (Calgary, Alberta, Canada). Plasmids were introduced into *B. cenocepacia *by electroporation [64].

### Construction of the K56-2Δ*cepR *mutant

An unmarked *cepR *mutant was constructed in K56-2 following the procedure outlined by Flannagan et al. [65]. Briefly, two regions of homology flanking *cepR *were amplified using primers F1-M1868-UP-EcoRI, R1-M1868-UP-ClaI, F2-M1868-DW-ClaI and R2-M1868-DW-XbaI. The amplified products were digested with ClaI to remove an internal portion of *cepR *and ligated into pCR2.1TOPO, giving rise to plasmid TPCR2.1::H12. Plasmid TPCR2.1::H12 was digested with XbaI and EcoRI and the fragment was inserted into pGPI-SceI, giving rise to mutagenesis plasmid pGPI-SceI::H12. pGPI-SceI::H12 was introduced into *B. cenocepacia *by conjugation. A single crossover event in K56-2 was confirmed by PCR in Tp resistant clones. To these clones, pDAI-SceI was introduced by conjugation to obtain the double crossover event. The pDAI-SceI plasmid was resolved by curing the exconjugants in LB broth. PCR confirmed that the deletion had occurred.

### RNA manipulations

*B. cenocepacia *was subcultured to obtain an initial optical density at 600 nm (OD_600_) of 0.02 and grown for the time indicated below for each experiment without selection. Total RNA was isolated using a RiboPure bacterial RNA isolation kit (Ambion, Streetsville, Ontario, Canada). DNase treatment was performed, and samples were confirmed by PCR using *Taq *polymerase (Invitrogen) to be free of DNA prior to cDNA synthesis.

### Microarray sample preparation

Three independent RNA samples from *B. cenocepacia *strains grown for 16 h were used in microarray experiments. Gene expression profiles were generated using custom *B. cenocepacia *J2315 microarrays (Agilent, Santa Clara, CA) [34,66]. A reference pool of K56-2 cDNA was fluorescently labelled with Cy3 while the test cDNA samples (K56-2, K56-R2, K56-2*cciR *and K56-2*cepRcciIR*) were fluorescently labelled with Cy5. cDNA generation and labelling was performed using the CyScribe Post-Labelling kit (GE Healthcare, Wales) according to the manufacturer's protocol. Spike-in controls (Aglient) were included into the labelling procedure for quality control purposes. cDNA purification was performed by ethanol precipitation and the labelled cDNA was purified using a CyScribe GFX purification kit and eluted with water. Hybridization and washing of arrays was performed according to the two-colour microarray based gene expression analysis protocol (Agilent) with minor modifications where the 25× fragmentation buffer was omitted and the cDNA mix and 10× blocking agent were heat-denatured for 3 min at 98°C and cooled to room temperature before adding the hybridization buffer. Washing of microarrays was performed including acetonitrile as well as stabilization and drying solution (Agilent). A G2565 BA microarray scanner and the scan control software (Agilent) were used. Scanning resolution was set to 5 μm and the scan region was adjusted to 61 × 21.6 mm. The extended dynamic range function was switched on with 100% and 10% photomultiplier gain settings. Images were analysed with the feature extraction software (Agilent). Labelling, hybrizidation and scanning were performed by the Mahenthiralingam Laboratory, Cardiff University, Wales.

### Microarray data analysis

Microarray data analysis was performed using GeneSpring GX 7.3.1 software (Agilent). Initial data were preprocessed by employing the enhanced Agilent FE import method, and then per-spot and per-chip normalizations were performed for all arrays. Some variation was noted across the 16 arrays of the signal intensities of spike-in control genes (added prior to cDNA synthesis) and prelabeled control genes (added prior to hybridization) (data not shown). After filtering on flags (present/marginal versus absent), genes were selected on the basis of changes, for which a 2-fold cutoff was used for comparison. Subsequent to microarray analysis, certain genes were noted because they appeared to be in transcriptional units associated with differentially regulated genes.

### Microarray accession number

The entire microarray data set has been deposited in the ArrayExpress database  under accession number E-MEXP-2303.

### Cluster analysis and operon prediction

Microarray data was analyzed with Cluster [67] and visualized using Treeview to allow a visual comparison of expression levels for each gene in an operon. Operon prediction was performed by analysis of the *B. cenocepacia *J2315 genome at [30]. Adjacent genes on the same coding strand with less than 300 bp intergenic space between them were arranged in putative operons.

### Quantitative RT-PCR

The sigma factor gene *sigE *[39] (previously termed *sigA *[18]) (BCAM0918) was used as a reference standard as described previously [18]. Expression of *sigE *was not significantly altered according to microarray analysis (data not shown). RT-PCR was performed using an iScript Select cDNA synthesis kit (Bio-Rad). For quantitative RT-PCR (qRT-PCR), quantification and melting curve analyses were performed with an iCycler and iQ SYBR green Supermix (Bio-Rad) according to manufacturer's instructions. qRT-PCRs were performed in triplicate, and the data shown below represent data from at least two independent experiments. Relative expression values for each gene were calculated using the ΔΔ*C*_*t *_equation [68].

### Transcriptional fusions to *luxCDABE *(*lux*)

The 371 bp *afcA *promoter region was amplified using primers AfcAPromfor1 and AfcAPromrev1 and cloned into the XhoI-BamHI site upstream of *lux *in pMS402 [69]. The *aidA *transcriptional fusion was previously described [28]. Luminescence assays were carried out as previously described [18,28]. The level of promoter activity is expressed below as the ratio of luminescence to turbidity (CPS/OD_600_).

## Authors' contributions

EPO prepared samples for microarray experiments and performed qRT-PCR experiments. EPO and PAS analyzed and interpreted microarray data. DFV constructed the unmarked mutant. EPO, DFV and RJM performed promoter::*lux *reporter experiments. EPO and PAS conceived and designed the experiments. EPO and PAS wrote the manuscript. All authors read and approved the final manuscript.

## Supplementary Material

Additional file 1 **Genes with increased or decreased expression in *cepR*, *cciR *or *cepRcciIR *mutants compared to K56-2**. Microarray analysis of selected genes showing differential expression in *cepR*, *cciR *or *cepRcciIR *mutants compared to K56-2. Click here for file

Additional file 2 **Oligonucleotide primers used in this study**. Complete list of oligonucleotide primers used in this study. Click here for file
