# Supplementary material for: Reciprocal regulation by the CepIR and CciIR quorum sensing systems in Burkholderia cenocepacia
Source: BMC Genomics. 2009 Sep 17;10:441. doi: 10.1186/1471-2164-10-441 (PMC2753556; doi:10.1186/1471-2164-10-441)
Supplement: Additional file 1 — Genes with increased or decreased expression in cepR, cciR or cepRcciIR mutants compared to K56-2. Microarray analysis of selected genes showing differential expression in cepR, cciR or cepRcciIR mutants compared to K56-2. [file 1471-2164-10-441-S1.DOC]

Additional File 1. Genes showing differential expression in *cepR*, *cciR* or *cepRcciIR* mutants compared to K56-2.

| Gene | Fuction^a^ | Change (fold) for  K56-R2 (*cepR*)  vs K56-2^b^ | Change (fold) for  K56-2*cciR*  vs K56-2^b^ | Change (fold) for  K56-*2cepRcciIR*  vs K56-2^b^ |
| --- | --- | --- | --- | --- |
| BCAL0001 | sodium/hydrogen exchanger family protein |  |  | -3.1 |
| BCAL0002 | carboxylate-amine ligase YbdK |  |  | -2.8 |
| BCAL0003 | MarR family regulatory protein |  | 2.0 |  |
| BCAL0004 | cysteine peptidase, family C26 | -2.3 | 2.7 |  |
| BCAL0022 | putative branched-chain amino acid ABC | -2.2 |  |  |
| BCAL0025 | methyltransferase GidB | 2.4 |  |  |
| BCAL0033 | ATP synthase delta chain |  | 2.1 |  |
| BCAL0034 | ATP synthase alpha chain | -2.2 | 2.4 |  |
| BCAL0036 | ATP synthase beta chain | -3.2 | 2.5 |  |
| BCAL0037 | ATP synthase epsilon chain | -2.7 | 3.3 |  |
| BCAL0039 | periplasmic cyclohexadienyl dehydratase | -2.3 |  |  |
| BCAL0043 | putative extracellular ligand-binding protein |  | 2.0 |  |
| BCAL0044 | putative transposase | 2.1 |  |  |
| BCAL0047 | putative acyl-CoA dehydrogenase |  | -2.3 |  |
| BCAL0049 | putative aminotransferase | -2.1 | 4.3 |  |
| BCAL0051 | periplasmic solute-binding protein | -2.1 | 2.5 |  |
| BCAL0052 | putative oxidoreductase | -2.2 | 2.7 |  |
| BCAL0056 | AraC family regulatory protein | -3.6 |  |  |
| BCAL0064 | acetaldehyde dehydrogenase |  |  | 2.2 |
| BCAL0070 | plasmid stabilisation system protein | 2.4 |  |  |
| BCAL0077 | putative oxidoreductase | -2.1 |  |  |
| BCAL0081 | prophage integrase |  |  | -3.6 |
| BCAL0084 | putative phage transcriptional activator | -2.1 |  |  |
| BCAL0086 | hypothetical phage protein | 2.1 |  |  |
| BCAL0090 | putative phage tail assembly protein | -3.1 |  | -2.8 |
| BCAL0093 | putative phage tail protein |  |  | -2.5 |
| BCAL0094 | phage major tail tube protein |  | -2.3 | -4.9 |
| BCAL0101 | phage baseplate assembly protein | 2.1 |  |  |
| BCAL0110 | putative aminotransferase |  |  | -2.1 |
| BCAL0111 | putative TPR repeat protein | -2.2 |  | -2.2 |
| BCAL0112 | conserved hypothetical protein |  |  | -2.2 |
| BCAL0114 | flagellin (type II) | -2.4 | 2.2 |  |
| BCAL0123 | putative glycosyltransferase |  | 2.3 | 2.9 |
| BCAL0124 | flagellar regulon master regulator subunit FlhD | -2.3 | 2.1 |  |
| BCAL0125 | flagellar regulon master regulator subunit FlhC | -5.4 | 2.1 |  |
| BCAL0129 | chemotaxis two-component sensor kinase CheA | -2.9 | 2.4 |  |
| BCAL0133 | putative chemoreceptor glutamine deamidase cheD | -2.9 | 2.4 |  |
| BCAL0135 | chemotaxis protein CheY |  | 2.1 |  |
| BCAL0140 | flagellar biosynthetic protein FlhB | -3.3 | 3.3 | -3.2 |
| BCAL0142 | putative flagellar biosynthesis protein |  | 2.5 | -2.4 |
| BCAL0143 | putative flagellar biosynthesis protein |  | 2.7 |  |
| BCAL0144 | RNA polymerase sigma factor for flagellar |  |  | -2.5 |
| BCAL0145 | adenosylhomocysteinase | -2.8 |  |  |
| BCAL0146 | putative membrane protein | -2.4 |  |  |
| BCAL0147 | 5,10-methylenetetrahydrofolate reductase |  | 2.1 |  |
| BCAL0151 | extracellular ligand binding protein | -2.3 |  |  |
| BCAL0157 | conserved hypothetical protein | -2.0 |  |  |
| BCAL0160 | putative methylase |  | 2.4 |  |
| BCAL0177 | putative plasmid conjugal transfer protein | 2.0 |  |  |
| BCAL0185 | LysR family regulatory protein | 2.1 |  |  |
| BCAL0200 | putative lipoprotein | -2.2 |  |  |
| BCAL0203 | phosphatidylethanolamine-binding protein | 2.0 |  | 2.8 |
| BCAL0206 | putative pyruvate ferredoxin/flavodoxin |  |  |  |
| BCAL0206A | putative outer membrane protein |  | 2.5 | 2.6 |
| BCAL0210 | TetR family regulatory protein | -2.6 |  |  |
| BCAL0219 | elongation factor Tu | -2.2 |  |  |
| BCAL0225 | 50S ribosomal protein L7/L12 |  | 2.0 |  |
| BCAL0226 | DNA-directed RNA polymerase beta chain | -2.3 |  |  |
| BCAL0227 | DNA-directed RNA polymerase beta' chain | -2.5 |  |  |
| BCAL0228 | ATP-dependent DNA helicase RecQ |  | 2.2 |  |
| BCAL0231 | elongation factor G | -2.0 |  |  |
| BCAL0232 | elongation factor Tu (EF-Tu) | -2.1 |  |  |
| BCAL0233 | 30s ribosomal protein S10 |  | 2.2 |  |
| BCAL0235 | 50S ribosomal protein L4 |  | 2.1 |  |
| BCAL0236 | 50S ribosomal protein L23 |  | 2.2 |  |
| BCAL0237 | 50S ribosomal protein L2 |  | 2.3 |  |
| BCAL0238 | 30S ribosomal protein S19 |  | 2.6 |  |
| BCAL0239 | 50S ribosomal protein L22 |  | 2.8 |  |
| BCAL0240 | 30S ribosomal protein S3 |  | 2.7 |  |
| BCAL0241 | 50S ribosomal protein L16 |  | 2.9 |  |
| BCAL0242 | 50S ribosomal protein L29 |  | 2.8 |  |
| BCAL0243 | 30S ribosomal protein S17 |  | 3.5 |  |
| BCAL0245 | 50S ribosomal protein L24 |  | 2.4 | -2.2 |
| BCAL0246 | 50S ribosomal protein L5 |  | 2.6 |  |
| BCAL0247 | 30S ribosomal protein S14 | -2.1 | 2.2 |  |
| BCAL0248 | 30S ribosomal protein S8 |  | 2.6 |  |
| BCAL0249 | 50S ribosomal protein L6 |  | 2.9 |  |
| BCAL0250 | 50S ribosomal protein L18 |  | 3.1 |  |
| BCAL0251 | 30S ribosomal protein S5 |  | 2.9 |  |
| BCAL0252 | 50S ribosomal protein L30 |  | 3.4 |  |
| BCAL0253 | 50S ribosomal protein L15 |  | 3.6 |  |
| BCAL0254 | preprotein translocase SecY subunit |  | 2.0 |  |
| BCAL0255 | translation initiation factor IF-1 |  | 3.0 |  |
| BCAL0258 | 30S ribosomal protein S11 |  | 2.4 |  |
| BCAL0259 | 30S ribosomal protein S4 |  | 2.4 |  |
| BCAL0260 | DNA-directed RNA polymerase alpha chain |  | 3.0 |  |
| BCAL0261 | 50S ribosomal protein L17 |  | 3.1 |  |
| BCAL0269 | putative oxidoreductase |  |  | -4.3 |
| BCAL0270 | ferric reductase-like transmembrane component |  |  | -4.3 |
| BCAL0283 | putative ABC transporter permease | -3.2 | 2.1 |  |
| BCAL0286 | glycerophosphoryl diester phosphodiesterase | -2.2 |  |  |
| BCAL0287 | putative outer membrane protein | -2.1 |  |  |
| BCAL0289 | glutamate synthase large subunit | -2.1 |  |  |
| BCAL0290 | glutamate synthase small subunit | -2.8 |  |  |
| BCAL0291 | sodium:amino acid symporter family protein | -2.3 |  |  |
| BCAL0301 | ABC transporter ATP-binding protein |  | 2.1 |  |
| BCAL0302 | putative membrane protein | -2.0 | 2.5 |  |
| BCAL0304 | VacJ-like lipoprotein |  | 2.1 |  |
| BCAL0307 | ABC transporter ATP-binding protein | -2.5 |  |  |
| BCAL0309 | BolA-like protein |  | 2.1 |  |
| BCAL0310 | UDP-N-acetylglucosamine | -2.1 | 2.1 |  |
| BCAL0312 | histidinol dehydrogenase | -2.7 |  |  |
| BCAL0337 | putative membrane protein | -4.3 | 2.2 | -3.1 |
| BCAL0339 | putative lipoprotein | -3.3 |  | -3.1 |
| BCAL0340 | putative lipoprotein | -2.8 |  |  |
| BCAL0341 | conserved hypothetical protein | -2.5 |  | -2.1 |
| BCAL0342 | conserved hypothetical protein | -3.1 |  | -2.3 |
| BCAL0343 | conserved hypothetical protein | -3.2 | 2.1 |  |
| BCAL0344 | conserved hypothetical protein | -2.7 |  |  |
| BCAL0345 | conserved hypothetical protein | -5.1 | 2.8 | -2.1 |
| BCAL0346 | conserved hypothetical protein | -2.9 | 2.1 | -2.1 |
| BCAL0347 | protease associated ATPase ClpB |  | 2.1 |  |
| BCAL0348 | conserved hypothetical protein | -2.7 | 2.1 | -2.2 |
| BCAL0349 | putative outer membrane protein | -2.5 | 2.4 |  |
| BCAL0350 | conserved hypothetical protein | -3.0 |  |  |
| BCAL0356 | putative quinone oxidoreductase |  | 2.2 |  |
| BCAL0366 | nitroreductase family protein | -2.6 |  |  |
| BCAL0380 | ABC transporter ATP-binding subunit | -2.3 |  |  |
| BCAL0381 | putative lipoprotein | -3.1 | 3.1 | -2.1 |
| BCAL0385 | putative amino acid permease |  | 3.7 |  |
| BCAL0388 | putative monooxygenase | -2.3 |  |  |
| BCAL0394 | uracil-DNA glycosylase |  |  | -2.7 |
| BCAL0395 | putative adenylate cyclase | 2.4 |  |  |
| BCAL0400 | putative phosphoglycolate phosphatase |  | 2.5 |  |
| BCAL0403 | putative outer membrane-bound lytic murein |  |  | -2.6 |
| BCAL0406 | probable enoyl-CoA hydratase PaaG | 8.7 |  |  |
| BCAL0425 | conserved hypothetical protein |  |  | -2.1 |
| BCAL0432 | putative membrane protein | 2.3 |  |  |
| BCAL0433 | spermidine N(1)-acetyltransferase |  |  | -4.5 |
| BCAL0436 | glyoxalase/bleomycin resistance | -2.0 |  |  |
| BCAL0437 | O6-methylguanine-DNA methyltransferase |  |  | 2.3 |
| BCAL0438 | putative DNA-3-methyladenine glycosylase II |  |  | 2.4 |
| BCAL0439 | glutamate--cysteine ligase | -2.6 |  |  |
| BCAL0440 | putative exported protein |  | 4.4 |  |
| BCAL0441 | putative L-lactate permease |  | 2.2 |  |
| BCAL0449 | extracellular solute-binding protein |  |  | -2.4 |
| BCAL0465 | peptide deformylase |  | -2.0 |  |
| BCAL0479 | penicillin-binding protein |  |  | -3.4 |
| BCAL0480 | putative rod shape-determining protein |  |  | -3.3 |
| BCAL0481 | putative rod shape-determining protein |  |  | -2.8 |
| BCAL0488 | putative alcohol dehydrogenase | -2.4 |  |  |
| BCAL0509 | S-adenosylmethionine synthetase | -2.0 |  |  |
| BCAL0510 | conserved hypothetical protein | -2.4 |  | -5.6 |
| BCAL0514 | putative membrane protein | 2.7 |  |  |
| BCAL0520 | putative flagellar hook-length control protein | -4.6 | 2.3 | -2.5 |
| BCAL0521 | flagellar FliJ protein | -2.7 | 3.7 |  |
| BCAL0522 | flagellum-specific ATP synthase FliI | -3.1 | 3.2 |  |
| BCAL0523 | flagellar assembly protein FliH | -3.5 | 3.0 |  |
| BCAL0525 | flagellar M-ring protein FliF |  | 2.1 |  |
| BCAL0526 | flagellar hook-basal body complex protein FliE | -3.3 | 2.2 |  |
| BCAL0535 | sensor kinase protein |  |  | 2.5 |
| BCAL0544 | putative periplasmic dipeptide transport | -2.8 |  |  |
| BCAL0546 | putative dipeptide transport system permease | -3.1 |  | -2.2 |
| BCAL0547 | putative dipeptide ABC transporter ATP-binding | -2.1 |  | -2.3 |
| BCAL0548 | putative dipeptide ABC transporter ATP-binding | -2.1 |  |  |
| BCAL0557 | putative glutathione S-transferase protein |  |  | -2.6 |
| BCAL0558 | tRNA nucleotidyltransferase |  |  | -2.5 |
| BCAL0564 | flagellar basal-body rod protein FlgB (putative | -2.4 | 2.8 |  |
| BCAL0565 | flagellar basal-body rod protein FlgC (putative | -3.4 | 2.5 |  |
| BCAL0566 | basal-body rod modification protein FlgD | -4.9 | 2.7 | -2.6 |
| BCAL0567 | flagellar hook protein 1 FlgE1 | -4.5 | 2.1 | -2.7 |
| BCAL0568 | flagellar basal-body rod protein FlgF (putative | -5.5 | 2.5 | -2.7 |
| BCAL0569 | flagellar basal-body rod protein FlgG (distal | -5.2 | 2.4 | -2.9 |
| BCAL0570 | flagellar L-ring protein precursor (basal body | -2.8 | 2.2 | -2.2 |
| BCAL0571 | flagellar P-ring protein precursor (basal body | -2.3 | 2.3 | -2.3 |
| BCAL0572 | peptidoglycan hydrolase FlgJ (muramidase FlgJ) | -2.6 |  | -2.7 |
| BCAL0573_J_0 | putative transposase |  |  | 2.1 |
| BCAL0577 | flagellar hook-associated protein 3 (HAP3) | -2.5 |  |  |
| BCAL0578 | putative permease | -2.9 |  |  |
| BCAL0583 | putative membrane protein |  | 2.2 |  |
| BCAL0584 | putative outer membrane porin protein |  |  | -2.7 |
| BCAL0584 | putative outer membrane porin protein | -2.3 |  |  |
| BCAL0585 | conserved hypothetical protein |  |  | 3.0 |
| BCAL0591 | putative transposase |  |  | -2.9 |
| BCAL0593 | putative oxidoreductase |  |  | -2.2 |
| BCAL0594 | putative exported protein | -2.3 |  |  |
| BCAL0597 | putrescine ABC transporter ATP-binding protein | -3.2 |  |  |
| BCAL0598 | putrescine ABC transport system, binding | -2.4 |  | -3.2 |
| BCAL0600 | putative glutamine synthetase |  |  | -2.4 |
| BCAL0602 | MerR family regulatory protein |  | 2.1 |  |
| BCAL0606 | putative transport related, membrane protein | -2.1 |  |  |
| BCAL0610 | conserved hypothetical protein |  |  | 2.1 |
| BCAL0644 | dihydrodipicolinate synthetase family protein | -2.5 |  |  |
| BCAL0646 | putative inward rectifier potassium channel |  | 2.0 |  |
| BCAL0653 | putative membrane protein | -2.1 |  |  |
| BCAL0659 | allophanate hydrolase subunit 1 | -2.5 |  |  |
| BCAL0660 | biotin carboxylase | 2.2 |  |  |
| BCAL0664 | biotin synthase |  | 2.5 |  |
| BCAL0676 | putative short chain dehydrogenase |  |  | -4.4 |
| BCAL0677 | thiol:disulfide interchange protein |  |  | -4.4 |
| BCAL0683 | conserved hypothetical protein | 3.1 | -2.6 |  |
| BCAL0693 | Bordetella pertussis Bvg accessory factor family |  |  | 2.2 |
| BCAL0700 | putative lipoprotein | -2.6 |  |  |
| BCAL0704 | D-alanyl-D-alanine carboxypeptidase |  |  | -2.7 |
| BCAL0719 | CAIB/BAIF family protein | 2.6 |  |  |
| BCAL0730 | ammonium transporter family protein | -2.1 |  |  |
| BCAL0731 | glutamate--cysteine ligase |  | -2.2 |  |
| BCAL0744 | Appr-1-p processing enzyme family protein | -2.1 |  |  |
| BCAL0746 | conserved hypothetical protein |  | 2.1 |  |
| BCAL0747 | putative methyltransferase | -2.5 |  |  |
| BCAL0754 | putative cytochrome c oxidase subunit III | -2.6 |  |  |
| BCAL0757 | putative membrane protein |  |  | -2.4 |
| BCAL0758 | putative cytochrome oxidase assembly protein |  |  | -2.3 |
| BCAL0766 | putative branched-chain amino acid transport | -2.0 |  |  |
| BCAL0769 | conserved hypothetical protein | 2.5 |  |  |
| BCAL0778 | putative N-acetylglucosamine-6-phosphate |  |  | -2.2 |
| BCAL0779 | putative phosphosugar-binding protein |  |  | -2.3 |
| BCAL0780 | putative multiphosphoryl transfer protein |  |  | -2.3 |
| BCAL0781 | phosphotransferase system, IIbc component |  | 2.3 |  |
| BCAL0784 | cytochrome d ubiquinol oxidase subunit II | -2.0 |  |  |
| BCAL0785 | cytochrome d ubiquinol oxidase subunit I | -2.5 |  |  |
| BCAL0786 | putative membrane protein | -2.9 |  |  |
| BCAL0792 | putative maleylacetoacetate isomerase | -2.2 |  |  |
| BCAL0797 | putative histidinol-phosphate aminotransferase | -2.3 |  |  |
| BCAL0799 | ribosomal L25p family protein |  | 2.0 |  |
| BCAL0812 | sigma-54 modulation protein | -2.8 |  |  |
| BCAL0815 | OstA-like protein |  |  | -2.4 |
| BCAL0816 | putative exported protein |  |  | -2.6 |
| BCAL0817 | putative 3-deoxy-D-manno-octulosonate |  |  | -2.4 |
| BCAL0822 | NUDIX hydrolase | -2.9 |  |  |
| BCAL0823 | putative formyltetrahydrofolate deformylase |  |  | -2.3 |
| BCAL0829 | putative 3-methyladenine DNA glycosylase | 2.5 |  |  |
| BCAL0831 | putative storage protein | -5.2 |  | -4.0 |
| BCAL0832 | putative poly-beta-hydroxy-butyrate storage | -2.4 |  |  |
| BCAL0833 | putative Acetoacetyl-CoA reductase | -2.6 |  | -3.6 |
| BCAL0834 | putative membrane protein | -2.4 |  |  |
| BCAL0839 | putative polysaccharide deacetylase |  | 2.5 |  |
| BCAL0841 | putative membrane protein |  |  | 2.2 |
| BCAL0851 | putative iron-sulphur cluster containing |  |  | -2.0 |
| BCAL0863 | cysteine peptidase, family C56 | -2.3 |  |  |
| BCAL0865 | RpiR family regulatory protein | -2.8 |  |  |
| BCAL0867 | YjgF family protein | -2.9 | 3.2 | 2.4 |
| BCAL0871 | conserved hypothetical protein |  |  | 2.0 |
| BCAL0882 | putative phospholipase |  | 2.1 |  |
| BCAL0883 | TetR family regulatory protein | -2.9 |  |  |
| BCAL0884 | putative acyl-CoA dehydrogenase oxidoreductase | -2.6 |  |  |
| BCAL0885 | putative 3-hydroxyacyl-CoA dehydrogenase | -2.1 |  |  |
| BCAL0895 | putative peptidyl-prolyl cis-trans isomerase | -2.2 |  |  |
| BCAL0897 | dimethyladenosine transferase | -2.6 |  |  |
| BCAL0898 | putative membrane protein |  | 2.8 |  |
| BCAL0900 | conserved hypothetical protein |  |  | -3.9 |
| BCAL0917 | putative oxidoreductase | -2.4 |  |  |
| BCAL0921 | fatty acid desaturase |  |  | -3.4 |
| BCAL0922 | 2Fe-2S ferredoxin |  |  | -2.6 |
| BCAL0927 | putative exported protein |  |  | 2.1 |
| BCAL0928 | conserved hypothetical protein |  |  | -2.2 |
| BCAL0931 | conserved hypothetical protein |  |  | -2.5 |
| BCAL0932 | conserved hypothetical protein |  |  | -2.9 |
| BCAL0933 | putative ATP-dependent RNA helicase 1 | -2.8 |  | -2.5 |
| BCAL0940 | putative transglycosylase | 3.0 |  |  |
| BCAL0957 | succinyl-CoA ligase alpha-chain |  | 2.1 |  |
| BCAL0959 | putative type IV pilin protein | 2.0 |  |  |
| BCAL0985 | Rieske iron-sulphur protein |  |  | -2.2 |
| BCAL0986 | serine peptidase, family S49 |  | 2.2 |  |
| BCAL0999 | sigma-E factor negative regulatory protein 2 |  |  | -3.4 |
| BCAL1008 | pyridoxal phosphate biosynthetic protein PdxJ 2 | -2.3 |  |  |
| BCAL1030 | Pirin-like protein |  |  | 2.0 |
| BCAL1036 | alpha,alpha-trehalose-phosphate synthase |  | 2.1 | 2.5 |
| BCAL1036 | alpha,alpha-trehalose-phosphate synthase |  |  |  |
| BCAL1040 | glycosyl transferases group 1 protein |  | 2.2 | 2.3 |
| BCAL1050 | putative glycosyltransferase | -2.1 |  |  |
| BCAL1051 | radical SAM superfamily protein | -2.0 |  |  |
| BCAL1052 | conserved hypothetical protein | -2.2 |  |  |
| BCAL1053 | putative membrane protein | -2.2 |  |  |
| BCAL1057 | histidine ABC transporter ATP-binding protein |  | 2.0 |  |
| BCAL1058 | AraC family regulatory protein | -2.5 | 2.3 | -2.7 |
| BCAL1062 | succinylglutamic semialdehyde dehydrogenase | -2.3 |  | -2.1 |
| BCAL1063 | succinylarginine dihydrolase | -2.3 |  |  |
| BCAL1064 | putative succinylglutamate desuccinylase |  | 2.3 |  |
| BCAL1065 | periplasmic solute-binding protein |  | 2.1 |  |
| BCAL1067 | putative transposase |  |  | -4.1 |
| BCAL1068 | conserved hypothetical protein | -2.0 | 2.2 |  |
| BCAL1079 | multidrug resistance protein MdtC |  | 2.0 |  |
| BCAL1090 | ABC transporter ATP-binding protein |  |  | 2.1 |
| BCAL1092 | ABC transporter extracellular solute-binding | -2.2 |  |  |
| BCAL1098 | putative exodeoxyribonuclease V alpha chain |  | 2.2 | 2.4 |
| BCAL1102 | putative lipoprotein |  |  | 3.0 |
| BCAL1103 | putative OsmB-like lipoprotein |  | 2.3 | 5.3 |
| BCAL1112 | putative phosphodiesterase | 8.7 |  | 2.1 |
| BCAL1119 | conserved hypothetical protein | 2.3 |  |  |
| BCAL1124 | conserved hypothetical protein | 2.0 |  |  |
| BCAL1141 | putative transposase |  | 2.2 |  |
| BCAL1145 | putative membrane protein |  | 2.1 |  |
| BCAL1164 | putative transposase |  |  | 2.1 |
| BCAL1167 | putative exported protein |  |  | -2.0 |
| BCAL1172 | conserved hypothetical protein |  | 2.5 |  |
| BCAL1174 | putative transposase | 2.2 |  |  |
| BCAL1179 | LysR family regulatory protein | -2.8 |  |  |
| BCAL1180 | LysR family regulatory protein | -2.2 |  |  |
| BCAL1187 | conserved hypothetical protein | -2.1 |  |  |
| BCAL1189 | putative exported protein |  | 2.0 |  |
| BCAL1202 | putative membrane protein |  | 2.4 |  |
| BCAL1203 | conserved hypothetical protein | 2.1 |  |  |
| BCAL1211 | conserved hypothetical protein |  | 2.2 |  |
| BCAL1212 | 2-oxoisovalerate dehydrogenase alpha subunit | -5.6 | 2.1 |  |
| BCAL1213 | 2-oxoisovalerate dehydrogenase beta subunit | -6.0 | 2.6 |  |
| BCAL1214 | lipoamide acyltransferase component of | -4.2 | 2.8 |  |
| BCAL1215 | dihydrolipoamide dehydrogenase | -3.7 | 2.8 |  |
| BCAL1218 | putative agmatinase | -4.1 |  |  |
| BCAL1220 | putative transport-related, membrane protein | -2.0 |  |  |
| BCAL1236 | putative exported protein | -23.9 | 8.7 | -30.7 |
| BCAL1271 | phosphate transport system permease protein | -2.5 |  |  |
| BCAL1275 | phosphate regulon two-component regulatory | -2.5 |  |  |
| BCAL1279 | putative exported protein | 2.1 |  |  |
| BCAL1280 | putative hydrolase |  |  | 2.3 |
| BCAL1282 | putative membrane protein | 2.4 |  |  |
| BCAL1290 | putative undecaprenol kinase | 2.1 |  |  |
| BCAL1295 | conserved hypothetical protein | -2.6 | 2.5 |  |
| BCAL1302_J_0 | conserved hypothetical protein (pseudogene) |  | 2.5 |  |
| BCAL1302_J_1 | conserved hypothetical protein (pseudogene) |  | 2.2 |  |
| BCAL1305 | conserved hypothetical protein |  | 2.0 |  |
| BCAL1311 |  |  | 2.1 |  |
| BCAL1315 | conserved hypothetical protein |  | 2.0 |  |
| BCAL1316 | conserved hypothetical protein |  | 2.6 |  |
| BCAL1339 | putative FAD monooxygenase |  | 2.8 |  |
| BCAL1345 | putative TonB-dependent siderophore receptor |  | 2.1 |  |
| BCAL1348 | FecCD-family membrane transporter protein | -2.0 |  |  |
| BCAL1354 | conserved hypothetical protein |  |  | -2.2 |
| BCAL1362 | conserved hypothetical protein |  | 2.0 |  |
| BCAL1364 | putative membrane protein |  | 2.2 |  |
| BCAL1368 | putative porin | -2.4 | 2.5 | -2.1 |
| BCAL1369 | putative RNA polymerase sigma factor FecI | 4.6 |  | 3.0 |
| BCAL1370 | iron uptake regulatory protein FecR | 2.7 |  | 2.1 |
| BCAL1375 | flavin reductase family protein | -2.2 |  |  |
| BCAL1377 | citrate transporter |  | 2.9 |  |
| BCAL1387 | putative phosphatase | 2.0 |  |  |
| BCAL1391 | putative cellulose biosynthesis protein | -2.2 |  |  |
| BCAL1395 | putative cellulose synthase catalytic subunit |  | 2.5 |  |
| BCAL1396 | putative membrane protein |  | 2.5 |  |
| BCAL1400 | sodium/hydrogen exchanger family protein | -2.1 |  |  |
| BCAL1409 | phosphoesterase family protein | 2.2 |  |  |
| BCAL1412 | NUDIX hydrolase | -2.0 |  |  |
| BCAL1413A | putative lipoprotein |  |  | 3.5 |
| BCAL1414 | putative hydrolase |  |  | 2.8 |
| BCAL1421 | putative branched amino acid transport system, |  | 2.3 |  |
| BCAL1426 | conserved hypothetical protein | -2.0 | 2.9 |  |
| BCAL1427 | myo-inositol catabolism protein | -2.2 | 2.5 |  |
| BCAL1429 | putative TPP-binding acetolactate synthase |  | 2.4 |  |
| BCAL1431 | putative ribose ABC transport system, substrate- |  | 3.8 |  |
| BCAL1433 | putative sugar transport system permease |  | 3.5 |  |
| BCAL1434 | putative myo-inositol catabolism protein |  | 2.5 |  |
| BCAL1436 | putative dehydratase |  | 3.2 |  |
| BCAL1442 | conserved hypothetical protein |  |  | 2.1 |
| BCAL1446 | putative lipoprotein |  | 2.7 |  |
| BCAL1451 | putative fosmidomycin resistance protein | -2.3 |  |  |
| BCAL1464 | putative membrane protein | 2.0 |  |  |
| BCAL1472 | succinyl-CoA:3-ketoacid-coenzyme A transferase | -2.1 |  |  |
| BCAL1477 | LysR family regulatory protein |  |  | 2.1 |
| BCAL1484 | 50S ribosomal protein L20 |  | 2.1 |  |
| BCAL1496 | putative exported protein | -2.5 |  |  |
| BCAL1497 | putative exported protein |  |  | -2.5 |
| BCAL1499 | putative exported protein |  |  | 2.2 |
| BCAL1504 | RNA pseudouridylate synthase family protein | -2.4 |  |  |
| BCAL1515 | 2-oxoglutarate dehydrogenase E1 component | -2.1 |  |  |
| BCAL1516 | dihydrolipoamide succinyltransferase component | -3.2 | 2.2 |  |
| BCAL1517 | dihydrolipoamide dehydrogenase | -3.1 | 2.7 |  |
| BCAL1518 | AFG1-like ATPase | -2.6 | 2.7 |  |
| BCAL1520 | putative lipoprotein | -2.1 |  |  |
| BCAL1528 | flp type pilus assembly protein | -2.1 |  |  |
| BCAL1530 | flp pilus type assembly protein | -3.2 |  | -2.0 |
| BCAL1531 | flp type pilus assembly protein | -2.2 |  |  |
| BCAL1532 | flp type pilus assembly protein |  |  | -2.2 |
| BCAL1533 | putative lipoprotein | -2.1 | 2.3 |  |
| BCAL1534 | putative exported protein | -3.3 | 2.6 | 2.1 |
| BCAL1548 | putative sugar ABC transport system, | -2.0 | 2.1 |  |
| BCAL1549 | putative sugar ABC transport system, membrane | -2.1 |  |  |
| BCAL1562 | hypothetical phage protein |  | 2.7 |  |
| BCAL1566 | putative phage tail protein | 2.2 |  |  |
| BCAL1568 | putative phage tail length determination |  | 2.2 |  |
| BCAL1569 | hypothetical phage protein | -2.0 | 2.0 |  |
| BCAL1575 | hypothetical phage protein | -3.1 |  |  |
| BCAL1577 | hypothetical phage protein |  |  | -2.4 |
| BCAL1588 | hypothetical phage protein | -2.0 |  |  |
| BCAL1589 | hypothetical phage protein | 8.7 |  |  |
| BCAL1593 | hypothetical phage protein | -2.0 |  |  |
| BCAL1602 | hypothetical phage protein | -2.5 |  | -2.0 |
| BCAL1604 | hypothetical phage protein | -2.2 |  |  |
| BCAL1612 | putative arginine-tRNA-protein transferase | 2.0 |  |  |
| BCAL1617 | putative hydratase | -2.0 |  |  |
| BCAL1622 | molybdenum-pterin binding protein II | 2.3 |  |  |
| BCAL1623 | conserved hypothetical protein | 2.4 |  |  |
| BCAL1624 | conserved hypothetical protein | 3.2 |  |  |
| BCAL1635a | putative exported protein |  | 3.1 |  |
| BCAL1636 | putative exported endonuclease | -2.1 |  |  |
| BCAL1639 | putative oxidoreductase |  | 2.1 |  |
| BCAL1649 | putative membrane protein |  |  | -3.0 |
| BCAL1653 | sulfate transport system permease protein | -2.4 |  |  |
| BCAL1654 | sulfate transport system permease protein | 2.4 |  |  |
| BCAL1657 | putative ribose transport system, | -3.0 |  |  |
| BCAL1658 | putative ribose ABC transporter ATP-binding | -2.0 |  |  |
| BCAL1662 | putative methyl-accepting chemotaxis protein |  | 3.2 |  |
| BCAL1671 | metallo peptidase, subfamily M23B |  |  | -3.3 |
| BCAL1674 | multidrug efflux system AmrA protein |  | 2.4 |  |
| BCAL1675 | multidrug efflux system transporter protein |  | 2.9 |  |
| BCAL1677 | putative type-1 fimbrial protein | -2.3 | 3.1 |  |
| BCAL1687 | putative exported protein |  |  | 3.4 |
| BCAL1688 | putative RNA polymerase sigma factor |  | -2.5 |  |
| BCAL1689 | MbtH-like protein |  | -2.2 |  |
| BCAL1690 | putative dioxygenase |  |  | 2.5 |
| BCAL1692 | putative iron transport-related membrane |  |  | 3.0 |
| BCAL1693 | putative iron transport-related membrane |  |  | 2.1 |
| BCAL1694 | putative iron transport-related exported |  |  | 2.4 |
| BCAL1696 | ornibactin biosynthesis non-ribosomal peptide |  | -2.4 |  |
| BCAL1697 | ornibactin biosynthesis non-ribosomal peptide |  |  | 2.4 |
| BCAL1698 | ornibactin biosynthesis protein |  | -2.8 |  |
| BCAL1699 | putative L-ornithine 5-monooxygenase |  | -2.8 |  |
| BCAL1700 | ornibactin receptor precursor |  |  | 2.2 |
| BCAL1700 | ornibactin receptor precursor |  | -2.2 |  |
| BCAL1701 | ornibactin synthetase F |  |  | 2.2 |
| BCAL1702 | putative ornibactin biosynthesis protein |  |  | 2.5 |
| BCAL1704 | conserved hypothetical protein | -2.1 |  |  |
| BCAL1715 | conserved hypothetical protein | 2.4 |  |  |
| BCAL1719 | putative citrate transporter | -2.2 |  |  |
| BCAL1722 | putative exported chitinase | -2.8 |  |  |
| BCAL1726 | putative oxidoreductase | -2.1 |  |  |
| BCAL1731 | Major Facilitator Superfamily protein | 2.4 |  |  |
| BCAL1732 | MarR family regulatory protein | 2.1 |  |  |
| BCAL1738 | taurine catabolism dioxygenase TauD, TfdA family | -2.1 |  |  |
| BCAL1740 | Major Facilitator Superfamily protein |  |  | 2.1 |
| BCAL1742 | extracellular solute-binding protein |  | -2.3 |  |
| BCAL1745 | binding-protein-dependent transport system inner | 2.2 |  |  |
| BCAL1749 | putative CoA-transferase |  | -2.1 |  |
| BCAL1753 | LysR family regulatory protein |  | 2.0 |  |
| BCAL1768 | LysR family regulatory protein |  |  | 2.0 |
| BCAL1769 | putative L(+)-mandelate dehydrogenase | -2.1 |  |  |
| BCAL1793 | putative metal ion transport protein |  |  | 2.2 |
| BCAL1796 | putative saccharopine dehydrogenase |  | 8.9 | 2.5 |
| BCAL1797 | AnsC family regulatory protein |  | 2.0 |  |
| BCAL1798 | putative exported protein |  | 2.2 | 2.3 |
| BCAL1799 | conserved hypothetical protein |  | 2.0 |  |
| BCAL1800 | conserved hypothetical protein |  | 2.2 |  |
| BCAL1801 | putative membrane protein |  | 2.2 |  |
| BCAL1806 | conserved hypothetical protein |  |  | 2.4 |
| BCAL1809 | putative transport-related, membrane protein | -2.1 |  |  |
| BCAL1814 | MerR family regulatory protein | -2.9 | -2.7 |  |
| BCAL1818 | metallo-beta-lactamase superfamily protein | -2.5 |  |  |
| BCAL1821 | putrescine transport system permease protein | -2.0 |  |  |
| BCAL1823 | putrescine ABC transporter ATP-binding protein | -2.2 | 2.1 |  |
| BCAL1824 | putrescine-binding periplasmic protein | -2.2 |  |  |
| BCAL1829 | putative outer membrane protein | -2.3 |  |  |
| BCAL1830 | putative 2-nitropropane dioxygenase | -2.6 |  |  |
| BCAL1831 | putative betaine aldehyde dehydrogenase | -3.1 |  |  |
| BCAL1832 | conserved hypothetical protein | 2.1 |  |  |
| BCAL1838 | UDP glycosyltransferase |  | 2.0 | -2.2 |
| BCAL1840 | conserved hypothetical protein |  | 2.4 |  |
| BCAL1847 | putative membrane protein |  | 3.1 |  |
| BCAL1852 | putative phosphoserine phosphatase | -2.2 |  |  |
| BCAL1857 | putative membrane protein |  |  | 2.1 |
| BCAL1859 | putative dihydrofolate reductase | -2.7 |  |  |
| BCAL1865 | ribosomal large subunit pseudouridine synthase |  |  | 2.6 |
| BCAL1870 | putative transcription accessory protein | -3.2 |  |  |
| BCAL1907 | putative multidrug resistance protein | -2.5 |  |  |
| BCAL1908 | lipoyl synthase | -2.5 |  |  |
| BCAL1909 | dihydrolipoyllysine-residue acetyltransferase | -2.6 |  |  |
| BCAL1910 | acetoin:2,6-dichlorophenolindophenol | -4.1 |  |  |
| BCAL1918 | conserved hypothetical protein |  |  | 2.2 |
| BCAL1920 | putative DNA-binding protein | -2.1 |  |  |
| BCAL1930 | SMR family transporter protein | -2.8 |  |  |
| BCAL1931 | putative |  |  | -2.3 |
| BCAL1932 | putative undecaprenyl-phosphate |  |  | -2.0 |
| BCAL1933 | L-arabinose formyltransferase |  |  | -2.1 |
| BCAL1944 | putative primosomal replication protein |  |  | -2.0 |
| BCAL1950 | hydroxypyruvate isomerase |  |  | 2.1 |
| BCAL1952 | conserved hypothetical protein | -2.9 | 2.1 |  |
| BCAL1956 | putative lipoprotein | -2.3 |  |  |
| BCAL1961 | putative exported protein |  |  | -3.1 |
| BCAL1962 | putative deoxyribonuclease | -2.4 |  |  |
| BCAL1969 | putative lipase | -2.2 |  |  |
| BCAL1972 | hypothetical protein | 2.1 |  |  |
| BCAL1977 | putative thiolase | -2.0 |  |  |
| BCAL1979 | putative fatty acid degradation protein | -2.4 |  |  |
| BCAL1980 | putative acyl-CoA synthetase | -2.5 |  |  |
| BCAL1985 | putative exported isomerase | -2.3 |  |  |
| BCAL1986 | putative acetyltransferase |  | 2.2 |  |
| BCAL1990 | glucose-6-phosphate isomerase | -2.6 |  |  |
| BCAL1992 | putative acyl-CoA thioesterase precursor |  |  | -3.6 |
| BCAL2008 | putative exported protein |  |  | -3.6 |
| BCAL2016 | 2-C-methyl-D-erythritol 4-phosphate | -2.9 |  |  |
| BCAL2020 | putative exported protein | -2.6 |  |  |
| BCAL2021 | putative penicillin-binding protein |  |  | -3.6 |
| BCAL2031 | putative membrane protein |  |  | 2.4 |
| BCAL2042 | putative transport-related membrane protein |  |  | -2.4 |
| BCAL2044 | muramoyltetrapeptide carboxypeptidase |  |  | -3.2 |
| BCAL2053 | ABC transporter ATP-binding protein | -2.4 |  |  |
| BCAL2058 | acetyltransferase (GNAT) family protein | -2.2 |  |  |
| BCAL2076 | putative RNA methylase protein |  | 2.6 | -2.1 |
| BCAL2077 | ribonuclease HII |  |  | -2.8 |
| BCAL2078 | putative lipid-A-disaccharide synthase |  |  | -4.0 |
| BCAL2079 | acyl-[acyl-carrier-protein]--UDP-N-acetylglucosamine |  |  | -3.6 |
| BCAL2080 | (3R)-hydroxymyristoyl-[acyl carrier protein] |  |  | -2.1 |
| BCAL2081 | UDP-3-O-[3-hydroxymyristoyl] glucosamine | -2.3 |  |  |
| BCAL2085 | 1-deoxy-D-xylulose 5-phosphate reductoisomerase | -2.2 |  |  |
| BCAL2087 | undecaprenyl pyrophosphate synthetase | -2.3 |  | -2.1 |
| BCAL2088 | ribosome recycling factor | -2.2 |  |  |
| BCAL2089 | uridylate kinase |  |  | -2.2 |
| BCAL2090 | elongation factor TS |  |  | -3.3 |
| BCAL2106 | glutathione peroxidase | -2.0 |  |  |
| BCAL2118 | isocitrate lyase |  | -2.6 | 3.1 |
| BCAL2123 | conserved hypothetical protein |  |  | -2.6 |
| BCAL2124 | AraC family regulatory protein |  |  | 2.5 |
| BCAL2129 | ABC transporter ATP-binding protein | -2.1 |  |  |
| BCAL2133 | putative membrane protein |  |  | 2.4 |
| BCAL2136A | putative exported protein |  | 2.5 |  |
| BCAL2141 | cytochrome O ubiquinol oxidase protein | -3.7 |  | -2.1 |
| BCAL2142 | cytochrome o ubiquinol oxidase subunit III | -2.2 |  | -2.2 |
| BCAL2143 | ubiquinol oxidase polypeptide I | -3.4 |  | -2.6 |
| BCAL2147 | tRNA(Ile)-lysidine synthase |  | 2.0 |  |
| BCAL2148 | acetyl-coenzyme A carboxylase carboxyl | -3.6 | 2.6 |  |
| BCAL2149 | HhH-GPD superfamily base excision DNA repair |  | 3.2 |  |
| BCAL2150 | cysteinyl-tRNA synthetase |  | 3.3 |  |
| BCAL2153 | peptidyl-prolyl cis-trans isomerase B | -2.5 |  |  |
| BCAL2179 | enolase |  |  | -2.2 |
| BCAL2184 | putative TatD related DNase | -2.0 |  |  |
| BCAL2185 | lipoprotein releasing system ATP-binding | -2.3 |  |  |
| BCAL2187 | putative exported protein | 2.1 |  |  |
| BCAL2188 | putative single-stranded-DNA-specific |  | 3.1 |  |
| BCAL2191 | putative membrane protein |  |  | 2.8 |
| BCAL2192 | conserved hypothetical protein | 2.1 |  |  |
| BCAL2195 | co-chaperone protein HscB homolog |  | -2.1 |  |
| BCAL2196 | HesB family protein | 2.4 | -2.3 |  |
| BCAL2197 | putative iron-sulfur cluster scaffold protein | 2.3 | -2.2 |  |
| BCAL2198 | cysteine desulfurase | 2.0 | -2.8 |  |
| BCAL2199 | putative transcriptional regulator protein | 2.9 | -2.5 |  |
| BCAL2207 | putative dihydrolipoamide dehydrogenase | -2.3 |  |  |
| BCAL2214 | putative DNA polymerase IV | 2.0 |  |  |
| BCAL2219 | hypothetical protein (pseudogene) | -2.1 | -2.2 |  |
| BCAL2223 | putative nitrogen regulation protein NR(II) | -2.0 |  |  |
| BCAL2238 | putative glutamine synthetase |  |  | -2.0 |
| BCAL2238 | putative glutamine synthetase | -2.4 |  |  |
| BCAL2244 | urocanate hydratase | -2.1 |  |  |
| BCAL2245 | putative histidine utilization repressor | -2.3 |  |  |
| BCAL2248 | 4'-phosphopantetheinyl transferase superfamily |  | 2.2 |  |
| BCAL2249 | serine peptidase, family S33 | -2.1 |  |  |
| BCAL2260 | bifunctional enzyme CysN/CysC [includes: sulfate | 2.3 |  |  |
| BCAL2268 | putative stress protein |  | 3.6 |  |
| BCAL2270 | conserved hypothetical protein |  | 2.7 |  |
| BCAL2272 | conserved hypothetical protein | 2.1 |  |  |
| BCAL2279 | conserved hypothetical protein (pseudogene) |  | -3.3 |  |
| BCAL2290 | putative bacterioferritin ferredoxin protein |  | -2.1 |  |
| BCAL2297 | conserved hypothetical protein | 2.6 | -3.3 |  |
| BCAL2317 | family M14 unassigned peptidase |  | 2.8 |  |
| BCAL2318 | conserved hypothetical protein |  | 2.2 |  |
| BCAL2323 | putative glutathione S-transferase | -2.0 |  |  |
| BCAL2329 | NUDIX hydrolase | -2.4 | 2.8 |  |
| BCAL2330 | putative membrane protein | -2.3 | 3.3 |  |
| BCAL2331 | NADH dehydrogenase I chain N |  | 3.5 |  |
| BCAL2332 | NADH dehydrogenase I chain M | -2.3 | 3.1 |  |
| BCAL2333 | NADH-ubiquinone oxidoreductase I chain L | -2.7 | 2.9 |  |
| BCAL2334 | NADH-ubiquinone oxidoreductase I chain K | -2.3 | 3.0 |  |
| BCAL2335 | NADH dehydrogenase I chain J | -2.1 | 2.8 |  |
| BCAL2336 | putative NADH dehydrogenase I chain I |  | 3.7 |  |
| BCAL2337 | NADH dehydrogenase I chain H | -2.1 | 3.2 |  |
| BCAL2338 | putative NADH dehydrogenase I chain G | -2.1 | 2.8 |  |
| BCAL2339 | NADH dehydrogenase I chain F |  | 2.3 |  |
| BCAL2340 | putative NADH dehydrogenase I chain E |  | 2.4 |  |
| BCAL2341 | NADH dehydrogenase I chain D |  | 2.2 |  |
| BCAL2348 | polyribonucleotide nucleotidyltransferase | -2.5 |  |  |
| BCAL2350 | putative amino acid transporter exported |  | 2.4 |  |
| BCAL2351 | putative lipoprotein |  | 2.0 |  |
| BCAL2352 | putative carbonic anhydrase | -4.3 | 6.3 |  |
| BCAL2353 | putative sulfate transporter | -3.4 | 4.8 |  |
| BCAL2354 | 2-isopropylmalate synthase |  |  | -2.1 |
| BCAL2355 | putative phosphatidyltransferase | -2.2 |  |  |
| BCAL2357 | ketol-acid reductoisomerase | -3.6 |  |  |
| BCAL2358 | acetolactate synthase isozyme III small subunit | -3.2 |  |  |
| BCAL2370 | putative membrane protein | -2.0 |  |  |
| BCAL2376 | quaternary ammonium compound-resistance protein | 2.2 |  |  |
| BCAL2380 | two-component regulatory system, sensor kinase |  | 2.1 |  |
| BCAL2384 | quinone oxidoreductase | -2.8 |  |  |
| BCAL2400 | putative membrane protein |  | 2.0 |  |
| BCAL2401 | putative exported protein |  |  | 2.5 |
| BCAL2403 | putative LPS core biosynthesis protein |  | 2.1 |  |
| BCAL2408 | lipid A export ATP-binding/permease protein | -2.0 |  |  |
| BCAL2408 | lipid A export ATP-binding/permease protein |  | 2.0 |  |
| BCAL2428 | putative cytochrome C precursor-related protein | -2.3 |  |  |
| BCAL2437 | conserved hypothetical protein | -2.9 |  |  |
| BCAL2438 | putative membrane protein | 2.1 |  |  |
| BCAL2446 | putative aminotransferase | -2.1 |  |  |
| BCAL2447 | endoribonuclease L-PSP family protein | -2.6 |  |  |
| BCAL2448 | putative phenazine biosynthesis-like protein | -2.2 |  |  |
| BCAL2450 | putative metal transport integral membrane |  | -2.0 |  |
| BCAL2451 | putative membrane protein |  |  | 2.2 |
| BCAL2454 | topoisomerase IV subunit A |  | 2.0 |  |
| BCAL2457 | putative exported protein | -2.7 | 2.2 |  |
| BCAL2458 | rubredoxin |  | 2.0 |  |
| BCAL2461 | putative membrane protein | 2.2 |  |  |
| BCAL2466 | ecotin precursor |  | 2.3 |  |
| BCAL2467 | putative lipoprotein |  | 2.5 | 6.1 |
| BCAL2468 | putative membrane protein |  |  | 5.3 |
| BCAL2468 | putative membrane protein |  | 2.6 |  |
| BCAL2472 | alpha,alpha-trehalose-phosphate synthase | -3.0 | 3.1 | 2.1 |
| BCAL2473 | putative lipoprotein | -2.3 |  | 2.1 |
| BCAL2479 | putative IstB-like ATP binding protein | 2.3 |  |  |
| BCAL2490 | LysR family regulatory protein | 2.5 |  |  |
| BCAL2491 | putative exported protein | 2.1 |  |  |
| BCAL2497_J_0_1 |  |  |  | 2.1 |
| BCAL2502 | conserved hypothetical protein | 2.1 |  |  |
| BCAL2515 | conserved hypothetical protein | -2.8 |  |  |
| BCAL2521 | conserved hypothetical protein (fragment) | 2.1 |  |  |
| BCAL2533 | putative methionine aminopeptidase (pseudogene) | 2.2 |  |  |
| BCAL2540 | LysR family regulatory protein | 2.2 |  |  |
| BCAL2542 | putative 3-isopropylmalate dehydratase large | 2.0 |  |  |
| BCAL2547 |  | 4.4 |  | 2.3 |
| BCAL2554 | conserved hypothetical protein | 2.1 |  |  |
| BCAL2555 | conserved hypothetical protein | 2.1 |  |  |
| BCAL2565 | hypothetical protein |  | 2.1 | 4.0 |
| BCAL2567 | conserved hypothetical protein |  | 2.1 | 3.4 |
| BCAL2570 | AraC family regulatory protein |  | 2.1 |  |
| BCAL2573 | ABC transporter ATP-binding protein |  |  | -2.4 |
| BCAL2574_J_1 | conserved hypothetical protein (pseudogene) | 2.1 |  |  |
| BCAL2594 |  | 2.1 |  |  |
| BCAL2596 | conserved hypothetical protein |  | -2.1 |  |
| BCAL2597 | hypothetical protein | 2.2 |  |  |
| BCAL2605 | two-component regulatory system, sensor kinase |  |  | 2.2 |
| BCAL2606 | two-component regulatory system, response | 2.2 |  |  |
| BCAL2607 | putative exported protein | 2.4 | 2.4 | 6.2 |
| BCAL2608 | Pirin-like protein | 2.9 |  |  |
| BCAL2610 | histidine transport system permease protein |  |  | -2.7 |
| BCAL2612 | histidine ABC transporter ATP-binding protein |  |  | -2.2 |
| BCAL2615 | putative exported outer membrane porin protein |  |  | -3.0 |
| BCAL2618 | conserved hypothetical protein | -2.5 |  |  |
| BCAL2631 | phosphoenolpyruvate carboxylase |  | -2.1 |  |
| BCAL2635 | putative exported protein |  |  | 2.0 |
| BCAL2636 | putative fimbriae chaperone |  |  | 2.3 |
| BCAL2641 | putative ornithine decarboxylase |  |  | -3.5 |
| BCAL2642 | deoxycytidine triphosphate deaminase |  |  | -2.9 |
| BCAL2648 | putative outer membrane protein |  |  | -2.2 |
| BCAL2675 | putative DNA polymerase III chi subunit | -2.0 | 2.3 |  |
| BCAL2685 | putative sulfite reductase |  | 2.3 |  |
| BCAL2690 | short chain dehydrogenase | -2.9 |  |  |
| BCAL2695 | conserved hypothetical protein |  | 2.2 |  |
| BCAL2698 | putative membrane protein | 2.6 |  |  |
| BCAL2702 | putative acetyltransferase | -2.1 |  |  |
| BCAL2703 | conserved hypothetical protein |  |  | -5.2 |
| BCAL2705 | ABC transporter ATP-binding protein | -2.5 | 2.1 |  |
| BCAL2707 | putative transport system permease protein |  | 2.1 |  |
| BCAL2714 | 50S ribosomal protein L28 |  |  | -2.4 |
| BCAL2715 | 50S ribosomal protein L33 |  |  | -2.7 |
| BCAL2717 | putative nicotinate-nucleotide |  | 2.2 |  |
| BCAL2724 | isoleucyl-tRNA synthetase | -3.4 |  |  |
| BCAL2725 | signal peptidase II | -2.2 |  |  |
| BCAL2734 | conserved hypothetical protein |  | -2.7 |  |
| BCAL2735 | isocitrate dehydrogenase [NADP] | -2.4 |  |  |
| BCAL2736 | isocitrate dehydrogenase | -2.8 |  |  |
| BCAL2737 | putative pseudouridine synthase | -2.1 |  |  |
| BCAL2741 | putative exported protein | 2.6 |  |  |
| BCAL2748 | Major Facilitator Superfamily protein | 2.0 |  |  |
| BCAL2757 | superoxide dismutase SodB | -2.3 |  |  |
| BCAL2758 | putative exodeoxyribonuclease VII large subunit | -2.7 |  |  |
| BCAL2761 | putative 3-deoxy-manno-octulosonate | -3.6 |  |  |
| BCAL2776 | putative hydrolase |  |  | -2.7 |
| BCAL2777 | putative N-acetylmuramoyl-L-alanine amidase |  |  | -2.5 |
| BCAL2780 | putative thioredoxin protein |  | -2.3 |  |
| BCAL2786 | putative selenium-binding protein | -2.2 |  |  |
| BCAL2792 | putative tryptophan 2,3-dioxygenase | -2.2 |  |  |
| BCAL2795 | aldehyde dehydrogenase family protein | 2.1 |  |  |
| BCAL2806 | putative ABC transporter permease protein |  |  | -2.1 |
| BCAL2814 | ABC transporter ATP-binding protein | -2.3 |  |  |
| BCAL2816 | S-formylglutathione hydrolase | -2.8 |  |  |
| BCAL2820 | efflux system outer membrane protein | -2.1 |  |  |
| BCAL2827 | conserved hypothetical protein | 2.3 |  |  |
| BCAL2828 | putative exported protein |  |  | 2.5 |
| BCAL2829 | serine peptidase, subfamily S1B |  |  | 2.2 |
| BCAL2832 | D-alanyl-D-alanine |  | 2.2 |  |
| BCAL2840 | putative pyruvate kinase II protein |  |  | 2.1 |
| BCAL2844 | putative branched-chain amino acid | -2.2 |  |  |
| BCAL2859 | sigma-54 interacting response regulator protein |  | 2.1 | 2.3 |
| BCAL2859 | sigma-54 interacting response regulator protein |  |  |  |
| BCAL2860 | beta-hexosaminidase 1 | -2.0 |  |  |
| BCAL2870 | sigma-E factor regulatory protein RseB precursor |  |  | -3.5 |
| BCAL2925 | 50S ribosomal protein L19 | -2.2 | 2.6 |  |
| BCAL2931 | radical SAM superfamily protein | 2.0 |  |  |
| BCAL2933 | D-amino acid dehydrogenase small subunit | -4.9 |  | -2.6 |
| BCAL2937 | ABC transporter membrane permease | -2.1 |  |  |
| BCAL2944 | ADP-l-glycero-D-manno-heptose-6-epimerase | -2.8 | 2.7 |  |
| BCAL2945 | D-beta-D-heptose 7-phosphate kinase |  | 2.5 |  |
| BCAL2946 | putative UDP-glucose dehydrogenase | -2.0 |  |  |
| BCAL2951 | cytidylate kinase | -2.5 |  |  |
| BCAL2952 | 3-phosphoshikimate 1-carboxyvinyltransferase |  |  | -2.1 |
| BCAL2955 | phosphoserine aminotransferase | -2.0 |  |  |
| BCAL2964 | putative phage-related DNA-binding protein | 2.1 |  |  |
| BCAL2973 | putative exported protein |  | 2.7 |  |
| BCAL2975 | possible regulatory protein |  |  | 2.3 |
| BCAL2976 | NAD-dependent formate dehydrogenase gamma | -2.9 |  |  |
| BCAL2978 | NAD-dependent formate dehydrogenase alpha | -2.3 |  |  |
| BCAL2981 | conserved hypothetical protein |  | 2.5 |  |
| BCAL2984 | cysteine peptidase, family C26 |  |  | 2.0 |
| BCAL2992 | putative lipoprotein |  |  | -4.0 |
| BCAL3004 | putative chorismate mutase | -2.1 |  |  |
| BCAL3006 | cold shock-like protein CspA | -5.6 | 2.8 | -4.9 |
| BCAL3025 | cell division topological specificity factor |  |  | -2.4 |
| BCAL3026 | septum site-determining protein |  |  | -2.8 |
| BCAL3033 | probable outer-membrane lipoproteins carrier |  |  | -2.2 |
| BCAL3038 | ABC transporter ATP-binding component |  |  | -4.6 |
| BCAL3039 | ABC transporter, membrane permease |  |  | -4.0 |
| BCAL3040 | ABC transporter, membrane permease |  |  | -5.7 |
| BCAL3041 | maltose-binding protein |  |  | -6.2 |
| BCAL3055 | probable N utilization substance protein B |  | 2.1 |  |
| BCAL3056 | putative aminotransferase |  | 2.9 |  |
| BCAL3059A | putative exported protein | 2.1 |  | 3.1 |
| BCAL3064 | putative NAD-dependent deacetylase |  | 2.9 |  |
| BCAL3068 | methylmalonyl-CoA decarboxylase | -2.2 |  |  |
| BCAL3094 | oxygen-independent coproporphyrinogen III |  |  | 2.1 |
| BCAL3100 | putative branched-chain amino acid transport |  |  | -2.0 |
| BCAL3103 | UreD-family accessory protein | 2.3 |  |  |
| BCAL3114 | putative O-antigen exporter |  |  | -2.1 |
| BCAL3118 | UDP-N-acetylglucosamine-1-P transferase |  | 2.5 |  |
| BCAL3123 | acetyltransferase | -2.2 |  |  |
| BCAL3124 | glycosyltransferase | -2.5 | 3.1 |  |
| BCAL3125 | glycosyltransferase (pseudogene) |  | 2.6 |  |
| BCAL3129 | nucleotide sugar aminotransferase | -2.1 | 3.2 |  |
| BCAL3138 | dihydroorotase-like protein | -2.1 |  |  |
| BCAL3146 | 60 kDa chaperonin 1 | -4.1 |  | -2.4 |
| BCAL3147 | 10 kDa chaperonin 1 | -4.1 |  | -2.4 |
| BCAL3148 | conserved hypothetical protein | -2.1 |  |  |
| BCAL3152 | putative RNA polymerase sigma factor | 2.6 |  |  |
| BCAL3153 | putative lipoprotein |  |  | 2.3 |
| BCAL3154 | putative glycine-rich surface protein | 2.2 |  |  |
| BCAL3156 | putative periplasmic | 2.3 |  |  |
| BCAL3163 | putative nucleotidyltransferase | 2.4 |  |  |
| BCAL3176 | AraC family regulatory protein |  |  | 2.2 |
| BCAL3178 | LysR family regulatory protein | -2.0 |  |  |
| BCAL3184 | homogentisate 1,2-dioxygenase | -2.1 |  |  |
| BCAL3190 | IclR family regulatory protein |  | 2.3 | 2.6 |
| BCAL3192 | putative oxidoreductase | -2.4 |  |  |
| BCAL3194 | putative membrane protein |  |  | -2.6 |
| BCAL3195 | conserved hypothetical protein |  |  | -2.7 |
| BCAL3200 | putative TolQ transport transmembrane protein |  |  | -2.2 |
| BCAL3201 | putative TolR-related protein |  |  | -2.5 |
| BCAL3202 | possible TolA-related transport transmembrane |  | 2.2 |  |
| BCAL3203 | putative periplasmic TolB protein |  |  | -2.6 |
| BCAL3204 | putative OmpA family lipoprotein |  |  | -2.9 |
| BCAL3205 | putative exported protein |  |  | -2.3 |
| BCAL3205C | hypothetical protein | -2.0 |  |  |
| BCAL3217 | putative acetyltransferase protein |  | 3.0 |  |
| BCAL3218 | putative acyl-CoA transferase |  | 2.1 |  |
| BCAL3223_J_1 | putative capsule polysaccharide | -2.1 |  |  |
| BCAL3228 | hypothetical protein |  |  | -2.3 |
| BCAL3229 | conserved hypothetical protein |  |  | -3.0 |
| BCAL3229A | hypothetical protein |  |  | 2.3 |
| BCAL3236 | putative transposase | 2.2 |  |  |
| BCAL3239 | glucosyltransferase |  | 2.0 |  |
| BCAL3240 | putative capsular polysaccharide transporter | -2.0 |  |  |
| BCAL3247 | mechanosensitive ion channel protein | -2.1 |  |  |
| BCAL3248_J_0 | putative transposase (pseudogene) | -2.4 |  |  |
| BCAL3257 | putative tRNA delta(2)-isopentenylpyrophosphate |  | 2.5 |  |
| BCAL3258 | tetracycline repressor protein | 2.2 |  |  |
| BCAL3268 | chorismate binding enzyme |  | 2.1 |  |
| BCAL3282 | putative phospho-2-dehydro-3-deoxyheptonate | -2.7 |  | -2.4 |
| BCAL3283 | conserved hypothetical protein | -2.9 |  |  |
| BCAL3292 | putative pyrroline-5-carboxylate reductase | -2.4 |  |  |
| BCAL3296 | putative 4-hydroxybenzoate transmembrane |  |  | -2.3 |
| BCAL3297 | putative ferritin DPS-family DNA binding | -2.8 |  | -3.8 |
| BCAL3298 | conserved hypothetical protein | -2.1 |  | -2.8 |
| BCAL3299 | peroxidase/catalase KatB | -2.3 |  | -4.0 |
| BCAL3307 | putative protein-export membrane protein | -2.1 |  |  |
| BCAL3310 | putative exported protein |  | 3.7 | -3.4 |
| BCAL3311 | putative exported protein |  | 2.2 | -4.0 |
| BCAL3312 | putative cytochrome b-561 membrane protein |  | 4.2 | -4.5 |
| BCAL3315 | mammalian cell entry related membrane protein |  | 2.3 |  |
| BCAL3317 | putative membrane protein | -2.3 |  |  |
| BCAL3336 | bifunctional purine biosynthesis protein | -2.3 |  |  |
| BCAL3338 | holliday junction DNA helicase | -2.0 |  |  |
| BCAL3354_J_0 | glutamate/aspartate ABC transporter ATP-binding | -2.9 |  |  |
| BCAL3354_J_1 | glutamate/aspartate ABC transporter ATP-binding | -2.3 |  |  |
| BCAL3356 | glutamate/aspartate transport system permease | -2.2 |  |  |
| BCAL3357 | glutamate/aspartate transport system permease | -2.3 |  |  |
| BCAL3358 | periplasmic glutamate/aspartate-binding protein | -2.4 |  |  |
| BCAL3364 | putative gluconokinase |  |  | -2.9 |
| BCAL3367 | phosphogluconate dehydratase |  |  | -2.2 |
| BCAL3368 | putative regulatory protein | -4.5 | -4.0 | -3.9 |
| BCAL3385 | putative L-idonate 5-dehydrogenase | 2.3 |  |  |
| BCAL3398 | putative competence-damaged related protein | -2.3 |  |  |
| BCAL3404 | L-arabinose ABC transporter ATP-binding protein | -4.2 |  | -2.0 |
| BCAL3405 | putative L-arabinose transport system, exported | -2.2 |  |  |
| BCAL3406 | putative dehydrogenase |  | 3.1 |  |
| BCAL3409 | IclR family regulatory protein |  | 2.6 |  |
| BCAL3411 | serine peptidase, family S10 | -2.7 |  | -2.3 |
| BCAL3412 | putative peptidoglycan biosynthesis-related |  | 2.2 |  |
| BCAL3413 | shikimate 5-dehydrogenase | -2.6 |  |  |
| BCAL3415 | conserved hypothetical protein |  | 2.0 |  |
| BCAL3419 | 3-dehydroquinate dehydratase | -2.0 |  |  |
| BCAL3424 | thiol peroxidase | -2.8 | 2.1 |  |
| BCAL3425 | putative sugar kinase | -2.1 |  |  |
| BCAL3427 | histone H1-like protein |  | 2.0 |  |
| BCAL3429 | putative ribonucleoside reductase | -2.0 |  |  |
| BCAL3439 | glutamate 5-kinase |  |  | 2.0 |
| BCAL3446 | type IV pilus assembly protein PilC |  |  | -2.1 |
| BCAL3452 | arginine biosynthesis bifunctional protein ArgJ | -2.3 |  |  |
| BCAL3456 | putative thioredoxin reductase | -2.1 |  |  |
| BCAL3458 | cell division protein FtsA |  | 2.2 | 2.5 |
| BCAL3460 | D-alanine--D-alanine ligase B |  |  | 2.9 |
| BCAL3461 | UDP-N-acetylmuramate--alanine ligase |  | 2.7 | 3.3 |
| BCAL3463 | cell division protein FtsW |  |  | 2.4 |
| BCAL3464 | UDP-N-acetylmuramoylalanine--D-glutamate ligase |  | 2.8 | 2.8 |
| BCAL3465 | phospho-N-acetylmuramoyl-pentapeptide-transferase |  |  | 3.0 |
| BCAL3468 | peptidoglycan synthetase FtsI |  |  | 2.2 |
| BCAL3471 | protein mraZ |  |  | 2.9 |
| BCAL3483 | hypothetical protein |  | 2.8 |  |
| BCAL3487 | carboxymuconolactone decarboxylase family |  | 2.2 |  |
| BCAL3490 | putative exported protein |  | 2.7 | 4.3 |
| BCAL3492 | putative exported protein |  | 4.1 | 8.3 |
| BCAL3501 | flagellar biosynthetic protein FliR |  | 2.5 |  |
| BCAL3505 | flagellar motor switch protein FliN | -2.5 | 2.6 |  |
| BCAL3506 | flagellar motor switch protein FliM |  | 2.4 |  |
| BCAL3507 | flagellar FliL protein |  | 2.7 |  |
| BCAL3511 | multidrug resistance transporter protein | -2.2 |  |  |
| BCAL3515 | general secretory pathway protein N |  | 2.1 |  |
| BCAL3522 | general secretory pathway protein H | -2.4 |  |  |
| BCAL3525 | general secretory pathway protein F |  | 2.2 |  |
| BCAL3530 | DNA-binding protein HU-alpha | -2.2 |  |  |
| BCAL3530 | DNA-binding protein HU-alpha |  | 3.2 |  |
| BCAL3531 | FAD dependent oxidoreductase |  | 2.8 |  |
| BCALr0080 | Perfect repeat flanking prophage |  | 2.7 | 3.7 |
| BCALr0164 | Perfect repeat flanking genomic island | 2.2 | 2.1 | 5.6 |
| BCALr1290 | Perfect repeat flanking genomic island | 2.1 | 2.2 | 4.7 |
| BCALr3075 | Perfect repeat flanking genomic island |  |  | 3.0 |
| BCAM0010 | 2-amino-3-ketobutyrate coenzyme A ligase |  | 2.2 |  |
| BCAM0011 | threonine 3-dehydrogenase |  | 2.1 |  |
| BCAM0013 | putative acetyltransferase - GNAT family |  | 2.1 |  |
| BCAM0014 | TetR family regulatory protein | 2.1 |  |  |
| BCAM0030 | conserved hypothetical protein | -3.8 |  | -2.1 |
| BCAM0031 | conserved hypothetical protein | -3.1 |  |  |
| BCAM0040 | AraC family regulatory protein | -2.8 |  |  |
| BCAM0041 | putative lipoprotein |  |  | -3.2 |
| BCAM0042 | putative aldo/keto reductase |  |  | -6.9 |
| BCAM0049 | CRP family regulatory protein |  | -2.4 |  |
| BCAM0059 | 3-oxoadipate CoA-transferase subunit B |  | 2.4 |  |
| BCAM0061 | putative 3-oxoadipate enol-lactonase I |  | 2.1 |  |
| BCAM0064 | conserved hypothetical protein |  |  | 2.0 |
| BCAM0065 | putative transporter - LysE family |  | -2.8 |  |
| BCAM0066 | putative lipoprotein | 2.3 |  |  |
| BCAM0080 | putative AMP-binding enzyme | -2.2 |  |  |
| BCAM0082 | putative polysaccharide deacetylase | 2.8 |  |  |
| BCAM0083 | hypothetical protein | 2.3 |  |  |
| BCAM0091 | putative membrane protein | -3.7 |  | -4.0 |
| BCAM0126 | putative AMP-binding enzyme |  | 2.0 |  |
| BCAM0132 | putative AMP-binding enzyme | 2.5 |  |  |
| BCAM0135 | putative quinone oxidoreductase | 2.7 |  |  |
| BCAM0138 | phosphoglycerate mutase family protein | -2.2 |  |  |
| BCAM0142 | putative acyl-CoA dehydrogenase family protein | -2.8 |  |  |
| BCAM0147 | hypothetical protein | 2.0 |  |  |
| BCAM0151 | conserved hypothetical protein (fragment) | 2.3 |  |  |
| BCAM0153 | 2-keto-3-deoxygluconate permease |  | -2.7 |  |
| BCAM0154 | 4-deoxy-L-threo-5-hexosulose-uronate | 2.4 | -2.3 |  |
| BCAM0160 | putative exported protein |  |  | -3.7 |
| BCAM0165 | conserved hypothetical protein |  | -2.3 |  |
| BCAM0166 | NADH dehydrogenase |  | -4.5 | 3.3 |
| BCAM0174 | conserved hypothetical protein | -2.1 |  |  |
| BCAM0184 | lectin |  | 2.0 | -2.1 |
| BCAM0186 | lectin | -7.5 | 3.1 | -3.2 |
| BCAM0187 | putative 2-isopropylmalate synthase | -3.2 |  | -3.5 |
| BCAM0189 | AraC family regulatory protein | -2.1 |  |  |
| BCAM0190 | putative aminotransferase - class III | -2.3 |  | -2.5 |
| BCAM0191 | putative non-ribosomal peptide synthetase | -2.3 |  |  |
| BCAM0193 | conserved hypothetical protein |  | 2.7 |  |
| BCAM0199 | outer membrane efflux protein | 2.1 |  |  |
| BCAM0201 | Major Facilitator Superfamily protein | 2.5 |  |  |
| BCAM0223 | putative haemagglutinin-related autotransporter | 2.9 |  |  |
| BCAM0233 | ArsR family regulatory protein |  | -2.0 |  |
| BCAM0238 | putative ion transporter |  | 2.0 |  |
| BCAM0239 | conserved hypothetical protein |  | 5.1 |  |
| BCAM0239a | N-acylhomoserine lactone synthase CciI |  | 52.1 |  |
| BCAM0240 | N-acylhomoserine lactone dependent regulatory CciR |  | 4.0 | -23.5 |
| BCAM0241 | putative 3-oxoacyl-[acyl-carrier-protein] |  | 3.3 |  |
| BCAM0253 | putative 3-oxoacyl-[acyl-carrier-protein] | -2.0 |  |  |
| BCAM0261 | putative branched-chain amino acid transporter | 2.2 |  |  |
| BCAM0263 | ABC transporter ATP-binding protein | -2.1 |  |  |
| BCAM0273 | conserved hypothetical protein |  |  | 2.2 |
| BCAM0277 | conserved hypothetical protein | -2.2 |  |  |
| BCAM0278 | putative heat shock protein | -3.0 | 3.0 | 2.3 |
| BCAM0280 | putative phospholipid-binding protein | -3.0 |  | 2.7 |
| BCAM0280A | conserved hypothetical protein |  |  | 2.3 |
| BCAM0284 | putative cytochrome c |  |  | 2.3 |
| BCAM0285 | conserved hypothetical protein |  |  | 2.5 |
| BCAM0290 | putative universal stress protein |  |  | 2.7 |
| BCAM0291 | putative universal stress protein |  | 2.2 |  |
| BCAM0292 | putative universal stress protein | -3.4 |  |  |
| BCAM0293 | putative acetate kinase | -2.4 | 2.2 |  |
| BCAM0294 | putative universal stress protein | -2.8 |  |  |
| BCAM0295 | conserved hypothetical protein | -2.2 |  |  |
| BCAM0297 | putative polymerase |  | 2.6 |  |
| BCAM0299 | putative zinc-binding alcoholdehydrogenase | -2.3 | 3.6 |  |
| BCAM0301 | putative membrane protein | -4.3 | 5.8 |  |
| BCAM0306 | putative membrane protein | -2.1 |  |  |
| BCAM0307 | conserved hypothetical protein | -2.4 |  | 2.4 |
| BCAM0308 | conserved hypothetical protein |  |  | 2.0 |
| BCAM0311 | putative 6-phosphofructokinase |  |  | 2.0 |
| BCAM0317 | putative membrane protein | -2.4 |  | 2.3 |
| BCAM0323 | two-component regulatory system, sensor kinase | 2.3 |  |  |
| BCAM0339 | conserved hypothetical protein |  | 2.2 |  |
| BCAM0376 | conserved hypothetical protein |  |  | 2.1 |
| BCAM0380 | putative exported protein | -2.2 |  |  |
| BCAM0385 | 1-aminocyclopropane-1-carboxylate deaminase | 2.1 |  |  |
| BCAM0387 | pyruvate dehydrogenase [cytochrome] | -2.3 |  | -7.2 |
| BCAM0397 | conserved hypothetical protein | -2.4 | 2.2 |  |
| BCAM0398 | conserved hypothetical protein |  |  | -2.0 |
| BCAM0416 | putative agmatinase | 2.0 |  |  |
| BCAM0442 | two-component regulatory system, sensor kinase |  | 3.8 | -2.4 |
| BCAM0457 | hypothetical protein |  | -2.1 |  |
| BCAM0459 | cysteine desulfurase | -4.0 | -4.9 | -3.3 |
| BCAM0461 | putative ABC transporter system permease | -3.0 |  |  |
| BCAM0467 | conserved hypothetical protein |  | -2.0 |  |
| BCAM0476a | hypothetical protein | -2.6 |  |  |
| BCAM0478 | glucosamine--fructose-6-phosphate | 2.3 |  |  |
| BCAM0483 | ADA-like AraC family regulatory protein |  |  | 2.1 |
| BCAM0489 | MerR family regulatory protein | 2.0 |  |  |
| BCAM0493 | putative membrane protein | -3.3 |  |  |
| BCAM0502 | conserved hypothetical protein |  | 3.5 | 3.0 |
| BCAM0507 | CsbD-like protein | 2.7 |  | 2.0 |
| BCAM0508 | putative periplasmic binding protein |  | 4.7 |  |
| BCAM0509 | putative FAD dependent oxidoreductase | -2.4 | 4.5 | 2.0 |
| BCAM0510 | putative membrane protein |  | 2.1 |  |
| BCAM0521 | putative IstB-like ATP binding protein | 2.2 |  |  |
| BCAM0525 | putative aminotransferase protein |  | 4.6 | 2.4 |
| BCAM0525A | hypothetical protein |  | 2.9 |  |
| BCAM0526 | pyridoxamine 5'-phosphate oxidase family |  | 3.0 |  |
| BCAM0528 | putative oxidoreductase/short-chain |  | 2.6 | 2.0 |
| BCAM0529A | putative membrane protein |  | 2.2 |  |
| BCAM0531 | 3-hydroxy-3-methylglutaryl-coenzyme a reductase | 2.4 |  |  |
| BCAM0534 | putative hydrolase |  | 2.4 |  |
| BCAM0548 | 60 kDa chaperonin 2 | -3.0 |  |  |
| BCAM0567 | Major Facilitator Superfamily protein | -2.1 |  |  |
| BCAM0569 | putative arylsulfatase |  | 2.1 |  |
| BCAM0574 | putative membrane protein | -2.3 |  |  |
| BCAM0576 | conserved hypothetical protein | -2.5 | 2.1 |  |
| BCAM0577 | Major Facilitator Superfamily protein | -2.5 | 2.1 | -2.1 |
| BCAM0578 | putative hydantoinase/oxoprolinase family | -3.0 | 3.5 | -2.0 |
| BCAM0582A | hypothetical protein | 2.1 |  |  |
| BCAM0602 | conserved hypothetical protein |  | 2.1 |  |
| BCAM0604 | Ornithine cyclodeaminase. |  | 2.7 |  |
| BCAM0605 | AnsC family regulatory protein | -2.0 |  |  |
| BCAM0609 | LysR family regulatory protein | 2.9 |  |  |
| BCAM0631 | AraC family regulatory protein | -2.0 |  |  |
| BCAM0633 | conserved hypothetical protein | -5.2 | 3.5 | -3.4 |
| BCAM0634 | hypothetical protein | -4.9 | 7.7 | -3.8 |
| BCAM0636 | alpha/beta hydrolase family protein | 2.2 |  |  |
| BCAM0640 | putative short-chain dehydrogenase | 2.2 |  |  |
| BCAM0641 | putative amidase | -2.2 |  |  |
| BCAM0642 | conserved hypothetical protein | -2.1 |  |  |
| BCAM0643 | conserved hypothetical protein | -2.1 |  |  |
| BCAM0661 | CoA-transferase family III protein | -2.5 | -2.6 |  |
| BCAM0666 | putative bacterial extracellular solute-binding | 2.0 |  |  |
| BCAM0668 | putative oxidoreductase | 2.0 |  |  |
| BCAM0670 | putative ABC transporter system permease | -3.1 |  |  |
| BCAM0673 | IclR family regulatory protein |  |  | 2.1 |
| BCAM0690 | putative outer membrane protein - OmpA family |  | 2.1 |  |
| BCAM0693 | conserved hypothetical protein |  | -2.5 |  |
| BCAM0699 | putative exported dehydrogenase | -2.6 |  |  |
| BCAM0705 | putative membrane protein |  | -2.9 |  |
| BCAM0713 | putative cobalt-zinc-cadmium resistance efflux |  | 2.7 |  |
| BCAM0715 | two-component regulatory system, sensor kinase |  | 2.1 |  |
| BCAM0716 | putative exported protein |  | 3.4 |  |
| BCAM0719 | putative exported protein | -2.1 |  |  |
| BCAM0721 | O-acetylhomoserine (thiol)-lyase | -2.7 |  |  |
| BCAM0729 | conserved hypothetical protein |  |  | 2.3 |
| BCAM0733 | putative oxidoreductase/monooxygenase | -2.0 |  |  |
| BCAM0740 | conserved hypothetical protein |  | -2.6 |  |
| BCAM0749 | lysine-specific permease | -2.4 |  |  |
| BCAM0752 | putative hydrolase |  |  | 2.1 |
| BCAM0753 | putative membrane protein | 2.9 |  |  |
| BCAM0761 | histidine transport system permease |  | 2.9 |  |
| BCAM0769 | putative sugar ABC transporter | -2.2 |  |  |
| BCAM0774 | poly[D-(-)-3-hydroxybutyrate] depolymerase |  | 2.3 | -2.1 |
| BCAM0776 | putative cNMP-binding domain protein |  | 2.6 |  |
| BCAM0780 | putative helicase | 2.3 |  | 2.3 |
| BCAM0783 | Major Facilitator Superfamily protein |  | 3.2 |  |
| BCAM0798 | AraC family regulatory protein | -2.0 |  |  |
| BCAM0803 | muconolactone delta-isomerase |  | 3.7 |  |
| BCAM0804 | catechol 1,2-dioxygenase 1 |  | 3.7 |  |
| BCAM0805 | muconate cycloisomerase I 1 |  | 2.9 | 2.0 |
| BCAM0810 | putative aromatic oxygenase |  | 3.1 |  |
| BCAM0811 | putative aromatic oxygenase |  | 3.1 |  |
| BCAM0831 | ABC transporter ATP-binding membrane protein |  |  | -2.0 |
| BCAM0835 | AraC family regulatory protein | 2.4 |  |  |
| BCAM0837 | putative membrane protein |  | 2.4 |  |
| BCAM0846 | conserved hypothetical protein | -2.9 |  |  |
| BCAM0847 | conserved hypothetical protein | -2.0 |  |  |
| BCAM0848 | glyoxalase/bleomycin resistance | -2.2 |  |  |
| BCAM0853 | conserved hypothetical protein | -2.7 |  |  |
| BCAM0860 | glycosyltransferase |  | 4.8 | 3.3 |
| BCAM0861 | putative glycosyltransferase |  | 2.3 |  |
| BCAM0862 | putative polymerase | 2.3 |  |  |
| BCAM0872 | GtrA family membrane protein | -2.1 |  |  |
| BCAM0885 | putative membrane protein | -2.3 |  |  |
| BCAM0896 | putative organic hydroperoxide resistance |  | 2.5 |  |
| BCAM0906 | putative dienelactone hydrolase family protein | -2.5 |  |  |
| BCAM0911 | 1-deoxy-D-xylulose 5-phosphate synthase |  | 2.4 |  |
| BCAM0924 | two-component regulatory system, response | 2.8 |  |  |
| BCAM0937 | putative monooxygenase, luciferase-like |  | 2.1 |  |
| BCAM0941 | 6-phosphogluconate dehydrogenase, | -2.3 |  |  |
| BCAM0942 | putative exported protein |  |  | -2.2 |
| BCAM0943 | conserved hypothetical protein |  |  | 3.4 |
| BCAM0945 | putative membrane protein |  |  | 3.5 |
| BCAM0949 | lipase | -3.1 |  |  |
| BCAM0950 | lipase chaperone | -2.5 |  |  |
| BCAM0953 | extracellular solute-binding protein | -2.0 |  |  |
| BCAM0957 | putative pepstatin-insensitive carboxyl | -2.2 | 2.6 | -3.3 |
| BCAM0961 | aconitate hydratase | -2.2 | 2.7 |  |
| BCAM0963 | putative exported protein | -2.3 | 2.0 |  |
| BCAM0965 | malate dehydrogenase | -2.7 | 2.6 |  |
| BCAM0971 | conserved hypothetical protein |  | 2.2 |  |
| BCAM0972 | citrate synthase | -2.8 |  |  |
| BCAM0972 | citrate synthase |  | 3.3 |  |
| BCAM0975 | conserved hypothetical protein | -2.8 | 2.6 |  |
| BCAM0978 | lysine-specific permease |  | 2.1 |  |
| BCAM0983 | 3-isopropylmalate dehydratase large subunit |  | -2.0 |  |
| BCAM0984 | 3-isopropylmalate dehydratase small subunit | -2.3 |  |  |
| BCAM0986 | aspartate-semialdehyde dehydrogenase |  |  | -2.3 |
| BCAM0989 | tRNA pseudouridine synthase A |  | 2.5 |  |
| BCAM0992 | putative DNA methylase |  |  | -2.2 |
| BCAM0993 | tryptophan synthase alpha chain | -2.5 |  |  |
| BCAM0995 | putative folC bifunctional protein [includes: | -2.5 |  |  |
| BCAM1003 | putative epimerase |  |  | -4.0 |
| BCAM1010 | putative UTP-glucose-1-phosphate |  | 3.3 | 3.1 |
| BCAM1012 | putative histone-like protein |  | 2.1 |  |
| BCAM1014 | putative 3-demethylubiquinone-9 | -2.7 |  |  |
| BCAM1015 | putative porin | -2.3 |  |  |
| BCAM1016 | putative ribonuclease |  |  | -3.0 |
| BCAM1030 | hypothetical phage protein | -2.5 |  |  |
| BCAM1051 | putative phage death-on-curing protein | 2.2 |  |  |
| BCAM1052 | hypothetical phage protein | 2.5 |  |  |
| BCAM1065 | putative phage head protein |  |  | -2.3 |
| BCAM1066 | hypothetical phage protein |  |  | -2.1 |
| BCAM1068 | putative exported phage protein |  |  | -2.2 |
| BCAM1070 | hypothetical phage protein |  |  | -2.0 |
| BCAM1071 | hypothetical phage protein |  |  | -2.1 |
| BCAM1072 | hypothetical phage protein |  |  | -2.4 |
| BCAM1078 | putative phage glucosaminidase |  |  | -2.8 |
| BCAM1079 | hypothetical phage protein |  |  | -2.1 |
| BCAM1082 | hypothetical phage protein |  |  | -2.1 |
| BCAM1083 | putative transmembrane phage protein | 2.0 |  |  |
| BCAM1084 | hypothetical phage protein |  |  | -3.1 |
| BCAM1086 | hypothetical phage protein |  |  | -2.7 |
| BCAM1087 | putative exported phage protein |  |  | -2.3 |
| BCAM1095 | putative phage lipoprotein |  |  | -3.3 |
| BCAM1102 | Major Facilitator Superfamily protein |  |  | 3.0 |
| BCAM1111 | ornithine decarboxylase | -2.3 | 2.0 |  |
| BCAM1112 | biodegradative arginine decarboxylase | -2.1 | 2.3 |  |
| BCAM1113 | putrescine transport protein | -2.3 | 2.1 |  |
| BCAM1127 | putative membrane protein |  | -2.2 |  |
| BCAM1128 | putative glycosyl transferase family protein |  | -2.0 |  |
| BCAM1132 | putative multidrug resistance transporter | 2.2 |  |  |
| BCAM1138 | Major Facilitator Superfamily protein |  | -2.4 |  |
| BCAM1139 | MarR family regulatory protein | -2.5 |  |  |
| BCAM1146 | putative flavoprotein monooxygenase | 2.4 |  |  |
| BCAM1150 | 3-hydroxyisobutyrate dehydrogenase | 2.2 |  |  |
| BCAM1151 | methylmalonate-semialdehyde dehydrogenase | -2.7 | 2.0 |  |
| BCAM1164 | conserved hypothetical protein |  |  | 2.4 |
| BCAM1166 | putative membrane protein |  | 2.9 |  |
| BCAM1167 | conserved hypothetical protein | -2.6 |  |  |
| BCAM1171 | Major Facilitator Superfamily protein |  | -2.5 |  |
| BCAM1172 | putative FAD dependent oxidoreuctase |  |  | -2.9 |
| BCAM1181 | putative phosphate acetyltransferase | -2.1 |  | -2.3 |
| BCAM1196 | putative methyl-accepting chemotaxis protein | -3.0 |  |  |
| BCAM1209 | glutamine ABC transporter, permease protein | 2.3 |  | -3.1 |
| BCAM1222 | putative cytochrome |  |  | -2.4 |
| BCAM1237 | conserved hypothetical protein |  | 2.1 |  |
| BCAM1239 | conserved hypothetical protein |  |  | 2.4 |
| BCAM1242A | putative exported protein | -2.6 |  |  |
| BCAM1244 | putative phosphonopyruvate decarboxylase | -2.1 | 2.5 |  |
| BCAM1245 | putative phosphoenolpyruvate phosphomutase/sugar | -2.0 | 2.1 |  |
| BCAM1246 | putative nucleotidyltransferase | -2.4 | 2.0 |  |
| BCAM1250 | probable acetyl-CoA hydrolase/transferase | -3.0 | 2.2 |  |
| BCAM1254 | MarR family regulatory protein |  |  | 2.1 |
| BCAM1256 | putative membrane protein |  | -2.3 |  |
| BCAM1257 | MerR family regulatory protein | 2.5 |  |  |
| BCAM1259 | RNA polymerase sigma factor | 2.2 |  |  |
| BCAM1260 | putative lipoprotein | 2.6 |  | 2.1 |
| BCAM1261 | putative membrane protein |  |  | 2.3 |
| BCAM1276 | ArsR family regulatory protein |  |  | 2.2 |
| BCAM1285 | putative membrane protein | 2.1 |  |  |
| BCAM1289 | Major Facilitator Superfamily protein |  |  | 2.2 |
| BCAM1290 | RpiR-family transcriptional regulator |  | 3.0 |  |
| BCAM1291 | L-asparaginase |  | 3.1 |  |
| BCAM1293 | ABC transporter, substrate-binding protein |  | 3.3 |  |
| BCAM1294 | ABC transporter, permease protein | -2.3 | 3.0 |  |
| BCAM1297 | metallo peptidase, family M55 |  | 4.0 |  |
| BCAM1305 | putative transcriptional regulator |  | -2.2 |  |
| BCAM1306 | putative amino acid permease | 2.2 |  |  |
| BCAM1309 | gamma-glutamyltransferase precursor 2 | -2.2 |  |  |
| BCAM1313 | putative amidase accessory protein | -2.2 |  |  |
| BCAM1314 | putative transporter protein AmiS | -2.4 |  |  |
| BCAM1315 | aliphatic amidase (acylamide amidohydrolase) | -3.0 | 2.1 |  |
| BCAM1316 | transport system outer membrane protein | -2.6 |  |  |
| BCAM1330 | putative polysaccharide export protein | 2.2 |  |  |
| BCAM1332 | putative membrane protein | 2.2 |  |  |
| BCAM1354 | putative membrane protein | -2.4 |  |  |
| BCAM1355 | putative phosphotransferase | 2.6 |  | 2.6 |
| BCAM1372 | 2,4-dihydroxyhept-2-ene-1,7-dioic acid aldolase | -3.9 |  | -2.5 |
| BCAM1377 | ABC transporter ATP-binding protein |  |  | -2.5 |
| BCAM1378 | ABC transporter permease protein | -2.4 |  | -2.7 |
| BCAM1398 | putative porin |  | -5.5 |  |
| BCAM1405 | levansucrase | -2.9 |  |  |
| BCAM1407 | DJ-1/PfpI family protein |  |  | -2.7 |
| BCAM1408 | xenobiotic reductase |  |  | -3.4 |
| BCAM1412 | conserved hypothetical protein | -3.5 |  | -6.8 |
| BCAM1413a | conserved hypothetical protein | -8.3 | 2.1 | -6.6 |
| BCAM1416 | LysE-family transporter |  | 3.7 |  |
| BCAM1417 | two-component regulatory system, sensor kinase | -2.2 | 8.1 |  |
| BCAM1418 | two-component regulatory system, response |  | 11.3 |  |
| BCAM1419 | efflux system outer membrane protein |  | 5.9 |  |
| BCAM1420 | efflux system transport protein |  | 11.0 |  |
| BCAM1421 | RND family efflux system transporter protein | -4.8 | 10.3 | -7.4 |
| BCAM1422 | putative cyclic nucleotide binding protein | -2.3 | 2.3 |  |
| BCAM1427 | LysE family transporter | -2.1 | -3.6 |  |
| BCAM1429 | IclR family regulatory protein |  | 2.3 |  |
| BCAM1447 | conserved hypothetical protein |  | -2.2 | -2.3 |
| BCAM1449A | putative amino acid permease | 2.1 |  |  |
| BCAM1453 | LysR family regulatory protein |  | 2.4 |  |
| BCAM1459 | short-chain fatty acid transporter | -2.1 | 2.4 |  |
| BCAM1460 | Major Facilitator Superfamily protein | -2.1 |  |  |
| BCAM1461 | conserved hypothetical protein | -3.1 |  | -2.4 |
| BCAM1462 | putative short chain dehydrogenase | -3.3 |  | -2.3 |
| BCAM1487 | putative ABC transporter, substrate-binding |  | -2.0 |  |
| BCAM1491 | putative exported protein |  | -2.0 | -2.2 |
| BCAM1497 | putative membrane protein |  |  | 2.0 |
| BCAM1498_J_1 | amino acid permease (pseudogene) | -2.5 |  |  |
| BCAM1500 | putative universal stress protein | -2.1 |  |  |
| BCAM1502 | conserved hypothetical protein | -2.8 |  |  |
| BCAM1503 | putative methyl-accepting chemotaxis protein |  | 2.1 |  |
| BCAM1504 | putative sigma-54 interacting transcriptional |  |  | -2.6 |
| BCAM1526 | LuxR superfamily regulatory protein | 2.1 |  |  |
| BCAM1543a | putative DNA-binding protein | 2.1 |  |  |
| BCAM1547 | dioxygenase superfamily protein | -2.7 |  |  |
| BCAM1570 | alcohol dehydrogenase | -2.7 |  | 2.1 |
| BCAM1570 | alcohol dehydrogenase | -2.5 |  |  |
| BCAM1583 | short-chain dehydrogenase | -2.3 |  |  |
| BCAM1588 | putative lyase | -2.5 | -2.1 |  |
| BCAM1591 | putative pyridoxal-phosphate dependent enzyme | -2.5 |  |  |
| BCAM1594 | conserved hypothetical protein |  |  | 2.6 |
| BCAM1622 | BNR/Asp-box repeat protein | -2.2 | -2.5 |  |
| BCAM1652 | putative lipoprotein |  |  | -4.0 |
| BCAM1660 | putative membrane protein | -2.2 |  |  |
| BCAM1661 | RNA polymerase sigma factor |  |  | -3.2 |
| BCAM1662 | putative exported protein | 2.6 |  |  |
| BCAM1669 | putative exported protein | 2.2 | -2.3 |  |
| BCAM1672 | mannonate dehydratase |  |  | -2.2 |
| BCAM1673 | Major Facilitator Superfamily protein | -2.0 |  | -3.4 |
| BCAM1680 | putative exported protein | 2.2 |  |  |
| BCAM1682 | putative short-chain dehydrogenase/reductase | -2.2 |  |  |
| BCAM1686 | putative nitrate transporter | -2.2 |  |  |
| BCAM1698 |  | 2.2 |  |  |
| BCAM1700 | putative acetyltransferase |  | -2.6 |  |
| BCAM1704 | 2,3-butanediol dehydrogenase | -2.8 |  |  |
| BCAM1713 | putative ionic antiporter |  |  | -2.1 |
| BCAM1724 | MarR family regulatory protein | -2.2 |  |  |
| BCAM1726 | putative exported protein |  |  | -3.2 |
| BCAM1729 | metallo peptidase, subfamily M20D | -2.1 |  |  |
| BCAM1733 | putative membrane protein |  |  | 2.4 |
| BCAM1734 | putative cytochrome C |  | 2.2 | 2.5 |
| BCAM1742 | putative exported protein | -4.4 | 2.1 |  |
| BCAM1743 | periplasmic solute-binding protein | -2.8 |  |  |
| BCAM1744 | serine peptidase, family S9 | -2.5 | 2.3 |  |
| BCAM1745 | putative magnesium-transporting ATPase |  | 3.2 | -2.5 |
| BCAM1746 | putative DNA-binding protein |  | -2.4 | 2.6 |
| BCAM1753A | putative sulfate transporter |  | 3.6 |  |
| BCAM1754 | putative mechanosensitive ion channel |  |  | -7.4 |
| BCAM1761 | putative lipoprotein | -2.1 |  |  |
| BCAM1770 | putative ABC transporter, substrate-binding | -2.3 |  |  |
| BCAM1771 | putative ABC transporter, permease protein | -2.3 |  |  |
| BCAM1772 | putative ABC transporter, permease protein | -2.5 | 2.3 |  |
| BCAM1773 | ABC transporter ATP-binding protein | -2.5 | 2.1 |  |
| BCAM1775 | putative transglycosylase associated protein |  |  | -2.7 |
| BCAM1776 | putative transposase |  |  | -3.4 |
| BCAM1777A | putative exported protein |  |  | -2.2 |
| BCAM1780 | peptidoglycan-binding lysm:peptidase m23b |  | 2.7 | 2.0 |
| BCAM1800 | conserved hypothetical protein |  |  | 2.0 |
| BCAM1804 | methyl-accepting chemotaxis protein |  | 2.1 |  |
| BCAM1833 | aconitate hydratase | -2.7 |  |  |
| BCAM1840 | putative acetyltransferase |  | 2.3 |  |
| BCAM1843 | putative dioxygenase |  | 2.2 |  |
| BCAM1846 | putative thiamine pyrophosphate binding protein | -2.3 |  |  |
| BCAM1856 | IclR family regulatory protein |  | 2.0 | 3.1 |
| BCAM1858 | conserved hypothetical protein |  |  | 2.4 |
| BCAM1869 | conserved hypothetical protein | -5.9 |  |  |
| BCAM1870 | N-acylhomoserine lactone synthase CepI | -67.0 |  | -6.9 |
| BCAM1871 | conserved hypothetical protein | -37.6 |  | -20.2 |
| BCAM1884 | putative DNA-binding phage protein |  |  | 2.1 |
| BCAM1893 | hypothetical phage protein | 3.0 |  |  |
| BCAM1895 | hypothetical phage protein | 2.0 |  |  |
| BCAM1924 |  |  |  | 2.0 |
| BCAM1927 | putative exported protein |  |  | -3.8 |
| BCAM1928 | putative transcription elongation factor |  |  | -2.9 |
| BCAM1931 | putative porin | -2.6 |  | -2.2 |
| BCAM1933 | putative cyclase | -2.2 |  |  |
| BCAM1940 | putative permease | 3.4 |  |  |
| BCAM1947 | putative quinoxaline efflux system transport | 2.3 |  |  |
| BCAM1950 | LysR family regulatory protein | 2.3 |  |  |
| BCAM1951 | TetR family regulatory protein (pseudogene) | -2.1 |  |  |
| BCAM1954 | sodium:dicarboxylate symporter | -3.7 | 2.5 |  |
| BCAM1957 | ABC transporter ATP-binding protein | 2.5 |  |  |
| BCAM1964 | putative exported protein |  | -2.9 | 2.8 |
| BCAM1966 | ArsR family regulatory protein |  | -2.0 |  |
| BCAM1974 | putative porin | 2.1 |  |  |
| BCAM1977 | putative amino acid permease | -3.2 |  | -2.2 |
| BCAM1981 | LuxR superfamily regulatory protein | -3.7 |  | -2.6 |
| BCAM1984 | LysR family regulatory protein | -2.1 |  |  |
| BCAM1985 | conserved hypothetical protein |  | 2.2 |  |
| BCAM1986 | Major Facilitator Superfamily protein |  | 2.2 |  |
| BCAM1995 | putative endoribonuclease |  | -2.2 |  |
| BCAM2000 | conserved hypothetical protein | -2.5 |  |  |
| BCAM2001 | short chain dehydrogenase | 2.0 |  |  |
| BCAM2005A | putative entericidin |  |  | 2.5 |
| BCAM2006 | putative aspartate carbomyltransferase | -2.8 |  |  |
| BCAM2009 | 2OG-Fe(II) oxygenase superfamily protein | 2.0 |  | 2.3 |
| BCAM2025 | sigma-54 interacting regulatory protein | 2.3 |  |  |
| BCAM2027 | putative exported protein |  | -2.0 | -2.7 |
| BCAM2027a | putative exported protein | -2.3 | -2.8 | -2.7 |
| BCAM2032 | putative NAD dependent epimerase/dehydratase |  | 2.2 |  |
| BCAM2039 | putative transcriptional regulator | 2.6 |  |  |
| BCAM2053 | putative type III secretion system protein | -3.0 | 2.2 | -2.8 |
| BCAM2055 | type III secretion system protein |  | -2.6 |  |
| BCAM2060 | putative divalent cation transporter |  | 2.7 |  |
| BCAM2062 | conserved hypothetical protein |  | 2.3 |  |
| BCAM2063 | putative carbohydrate-selective porin |  | 3.6 |  |
| BCAM2067 | putative undecaprenyl pyrophosphate synthetase | -4.6 |  | -3.8 |
| BCAM2071 | putative acetyltransferase | -2.1 |  |  |
| BCAM2073 | putative exported protein | -3.4 |  | 2.9 |
| BCAM2081 | conserved hypothetical protein | -2.1 |  |  |
| BCAM2082 | putative S-adenosylmethionine decarboxylase | 2.8 | -2.1 | -2.5 |
| BCAM2083 | putative membrane protein |  | 2.7 |  |
| BCAM2084 | conserved hypothetical protein | -2.3 |  |  |
| BCAM2085 | putative membrane protein | -2.3 | 2.0 |  |
| BCAM2086 | putative spermidine synthase | -2.0 |  |  |
| BCAM2089 | putative exported protein | -2.3 | 2.9 |  |
| BCAM2091 | putative acetyltransferase | 2.3 |  |  |
| BCAM2094 | putative gamma-glutamylputrescine synthetase |  | 2.3 |  |
| BCAM2095 | putative HTH transcriptional regulator | -2.8 | 2.3 |  |
| BCAM2104 |  | -2.5 |  |  |
| BCAM2106 | non-heme chloroperoxidase | -2.5 | 2.2 |  |
| BCAM2112 | NAD dependent epimerase/dehydratase family |  | 2.1 |  |
| BCAM2113 | Major Facilitator Superfamily protein | 2.2 |  |  |
| BCAM2114 | putative hydroxylase | -2.5 |  |  |
| BCAM2115 | putative 4-hydroxyphenylacetate 3-monooxygenase, | -2.4 |  |  |
| BCAM2118 | LysR family regulatory protein |  | 2.3 |  |
| BCAM2127 | putative dienelactone hydrolase family protein | -3.2 |  |  |
| BCAM2128 | putative short chain dehydrogenase | -2.2 |  |  |
| BCAM2129 | 2-amino-3-carboxymuconate 6-semialdehyde |  | 2.1 |  |
| BCAM2130 | 3-hydroxyanthranilate 3,4-dioxygenase |  | 2.3 |  |
| BCAM2139 | conserved hypothetical protein |  | 2.3 |  |
| BCAM2145 | putative GABA permease |  | 3.4 | 2.0 |
| BCAM2159 | putative exported protein | -2.2 |  |  |
| BCAM2161 | two-component regulatory system, sensor kinase |  |  | 2.7 |
| BCAM2162 | MarR family regulatory protein | 2.2 |  |  |
| BCAM2163 | putative monooxygenase |  | -2.0 |  |
| BCAM2167 | conserved hypothetical protein |  |  | 2.2 |
| BCAM2189 | Major Facilitator Superfamily protein | -2.0 |  |  |
| BCAM2190 | LysR family regulatory protein | -2.3 |  |  |
| BCAM2191 | enoyl-CoA hydratase/isomerase family |  | 2.1 |  |
| BCAM2192 | enoyl-CoA hydratase/isomerase family protein | -3.4 | 2.2 |  |
| BCAM2193 | putative 3-hydroxyisobutyrate dehydrogenase | -4.4 | 2.3 |  |
| BCAM2194 | methylmalonate-semialdehyde dehydrogenase | -4.3 | 2.5 |  |
| BCAM2195 | putative AMP-binding enzyme | -2.9 |  |  |
| BCAM2196 | putative acyl-CoA dehydrogenase | -2.3 |  |  |
| BCAM2199 | putative membrane protein | 3.4 |  |  |
| BCAM2201 | conserved hypothetical protein | -2.1 |  |  |
| BCAM2207 | conserved hypothetical protein | 2.9 |  |  |
| BCAM2209 | conserved hypothetical protein |  |  | 2.0 |
| BCAM2210 | putative membrane protein | 2.7 |  |  |
| BCAM2215 | putative copper resistance protein C precursor | -2.2 |  |  |
| BCAM2216 | putative exported protein | -2.7 | -2.3 |  |
| BCAM2224 | putative pyochelin receptor protein FptA |  | -4.1 | -2.1 |
| BCAM2231 | transcriptional regulator PchR | 2.4 |  |  |
| BCAM2237 | putative 2,2-dialkylglycine decarboxylase | -2.9 | -3.8 | -2.2 |
| BCAM2240 | AraC family regulatory protein | -2.0 | -2.0 |  |
| BCAM2245 | conserved hypothetical protein | 2.1 |  |  |
| BCAM2247 | putative amino acid ABC transporter ATP-binding | -2.7 | 2.2 |  |
| BCAM2248 | putative amino acid ABC transporter ATP-binding | -2.8 | 2.2 |  |
| BCAM2250 | putative amino acid transport system permease | -2.3 |  |  |
| BCAM2251 | putative amino acid solute binding component of | -2.5 |  |  |
| BCAM2264 | LysR family regulatory protein | 2.1 |  |  |
| BCAM2266 | putative amidohydrolase | -6.4 | -3.4 | -4.5 |
| BCAM2280 | putative solute-binding component of ABC | -2.1 |  |  |
| BCAM2282 | ABC transporter ATP-binding protein | -3.1 |  |  |
| BCAM2283 | putative short chain dehydrogenase | -2.7 |  |  |
| BCAM2285 | putative fumarylacetoacetate (FAA) hydrolase | -2.3 |  |  |
| BCAM2286 | putative short chain dehydrogenase |  | -2.2 |  |
| BCAM2287 | conserved hypothetical protein |  | 3.3 |  |
| BCAM2288 | putative short-chain dehydrogenase | -2.1 |  |  |
| BCAM2289 | conserved hypothetical protein |  | -2.2 | -2.2 |
| BCAM2296 | LysR family regulatory protein |  | 2.1 |  |
| BCAM2301 | putative methylamine dehydrogenase | 2.2 |  |  |
| BCAM2307 | zinc metalloprotease ZmpB | -2.3 | 2.1 | -2.4 |
| BCAM2311 | putative outer membrane porin protein |  |  | -2.2 |
| BCAM2315 | putative formyltetrahydrofolate deformylase | -2.0 | 2.2 |  |
| BCAM2316 | putative amino acid permease |  | 2.4 |  |
| BCAM2317 | putative exported protein | 2.0 |  |  |
| BCAM2318 | putative ferredoxin oxidoreductase protein | 2.8 | 2.2 |  |
| BCAM2319 | iron-sulphur Rieske protein |  | 2.0 |  |
| BCAM2325 | putative dipeptidase |  | 2.5 |  |
| BCAM2328 | putative exported protein with coagulation | 2.1 | 2.7 | 3.8 |
| BCAM2333 | putative glutathione-independent formaldehyde |  | 2.1 | -2.2 |
| BCAM2342 | putative betaine aldehyde dehydrogenase | -2.3 |  |  |
| BCAM2343 | putative choline dehydrogenase | -2.0 |  |  |
| BCAM2345 | hypothetical protein | 2.1 |  |  |
| BCAM2347 | putative lipoprotein | 2.0 |  |  |
| BCAM2350 | ABC transporter ATP-binding protein | -3.2 |  |  |
| BCAM2351 | putative transmembrane component of ABC | -3.0 | 2.5 |  |
| BCAM2352 | Major Facilitator Superfamily protein | 3.1 |  |  |
| BCAM2358 | putative esterase | 2.2 |  |  |
| BCAM2372 | putative acetyl-coenzyme A synthetase |  | 2.0 |  |
| BCAM2374 | putative methyl-accepting chemotaxis protein | -3.1 |  | -2.1 |
| BCAM2377 | putative exported protein |  | 2.3 |  |
| BCAM2379 | NUDIX hydrolase |  | 2.1 |  |
| BCAM2380 | putative D-isomer specific 2-hydroxyacid | 2.1 |  | 2.1 |
| BCAM2380 | putative D-isomer specific 2-hydroxyacid |  | -2.0 |  |
| BCAM2384 | putative ABC transporter system permease |  | 2.0 |  |
| BCAM2385 | rifampin ADP-ribosyl transferase | -2.7 |  |  |
| BCAM2388 | putative sarcosine oxidase gamma subunit |  | 2.1 |  |
| BCAM2389 | putative sarcosine oxidase alpha subunit |  | 2.0 |  |
| BCAM2400 | NAD dependent epimerase/dehydratase family |  | 2.8 |  |
| BCAM2404 | LysR family regulatory protein | -2.0 |  |  |
| BCAM2414 | conserved hypothetical protein | -2.3 |  |  |
| BCAM2429 | putative phospholipase C |  | 2.8 |  |
| BCAM2429a | putative lipoprotein |  | 2.2 |  |
| BCAM2430 | putative biotin carboxylase |  | 3.7 |  |
| BCAM2431 | putative enoyl coenzyme A hydratase-like |  | 4.6 |  |
| BCAM2432 | putative biotin-dependent carboxyl transferase | -2.1 | 4.2 |  |
| BCAM2433 | putative acyl-CoA dehydrogenase | -2.7 | 3.5 |  |
| BCAM2437 | vanillate O-demethylase oxidoreductase | -3.1 |  | -2.0 |
| BCAM2438 | putative lipoprotein |  | -2.1 |  |
| BCAM2465 | putative transport-related, membrane protein | -2.3 |  |  |
| BCAM2468 | putative aldehyde dehydrogenase family protein |  | 3.1 | 2.3 |
| BCAM2469 | putative amino-acid permease |  | 3.3 |  |
| BCAM2475 | putative membrane protein | -2.2 |  |  |
| BCAM2478 | serine-carboxyl peptidase |  |  | -3.0 |
| BCAM2485 | two-component regulatory system, sensor kinase | -2.0 |  |  |
| BCAM2490 | conserved hypothetical protein |  | -2.4 |  |
| BCAM2492 | conserved hypothetical protein | 2.2 |  |  |
| BCAM2496 | binding-protein-dependent transport system | -3.9 |  | -2.1 |
| BCAM2502 | 3-dehydroquinate dehydratase | -2.1 |  |  |
| BCAM2536 | putative alpha-beta hydrolase |  | 2.1 |  |
| BCAM2545 | Major Facilitator Superfamily protein |  |  | -2.2 |
| BCAM2546 | putative MlrC family protein | -2.2 |  |  |
| BCAM2554 | LysR family regulatory protein | -2.1 |  |  |
| BCAM2562 | putative succinate-semialdehyde dehydrogenase | -3.4 |  |  |
| BCAM2564 | putative aerotaxis receptor | -2.1 |  |  |
| BCAM2566 | putative GCN5-related N-acetyltransferase | -3.1 |  | -2.1 |
| BCAM2568 | putative beta-ketoadipyl CoA thiolase |  | 2.4 |  |
| BCAM2576 | putative ThiJ/PfpI family protein | 2.2 |  |  |
| BCAM2578 | putative short chain dehydrogenase protein | 2.2 |  |  |
| BCAM2581 | Major Facilitator Superfamily protein | -2.5 |  |  |
| BCAM2589 | IclR family regulatory protein |  | -2.2 |  |
| BCAM2590 | Major Facilitator Superfamily protein | -2.2 |  |  |
| BCAM2618 | putative periplasmic | -2.6 |  |  |
| BCAM2621_J_0 | putative porin-related protein (pseudogene) |  |  | 2.2 |
| BCAM2625 | conserved hypothetical protein | 3.1 |  |  |
| BCAM2626 | putative heme receptor protein | 3.4 | -2.2 | 2.7 |
| BCAM2627 | putative hemin ABC transport system protein | 3.1 |  | 2.9 |
| BCAM2632 | putative penicillin-binding protein | 2.1 |  |  |
| BCAM2639 | putative membrane protein |  | 2.3 | 2.7 |
| BCAM2652 | TetR family regulatory protein |  |  | 2.3 |
| BCAM2653 | putative exported protein | -2.3 |  |  |
| BCAM2656 | putative Hsp90 family protein |  |  | 2.2 |
| BCAM2659 | putative tartrate transporter | -2.5 |  |  |
| BCAM2670 | conserved hypothetical protein | 2.1 |  |  |
| BCAM2674 | putative cytochrome oxidase subunit I |  | -2.2 |  |
| BCAM2675 | putative cytochrome oxidase subunit II |  |  | 2.1 |
| BCAM2676 | putative membrane protein |  |  | 2.2 |
| BCAM2677 | putative membrane protein | 2.1 | -2.3 |  |
| BCAM2683 | putative cation-transporting ATPase membrane |  |  | 2.6 |
| BCAM2687 | MgtC family membrane protein |  |  | -2.4 |
| BCAM2689 | putative methyl-accepting chemotaxis protein |  | 2.0 |  |
| BCAM2700 | putative membrane protein | -5.8 |  | -2.1 |
| BCAM2701 | aconitate hydratase 1 | -3.7 |  | -3.5 |
| BCAM2702 | 2-methylcitrate synthase | -14.4 |  | -3.6 |
| BCAM2703 | probable methylisocitrate lyase | -8.3 |  | -3.0 |
| BCAM2707 | putative FAA-hydrolase family protein |  |  | 2.3 |
| BCAM2709 | conserved hypothetical protein | -2.5 |  |  |
| BCAM2729 | MerR family regulatory protein |  | 3.2 |  |
| BCAM2730 | putative tripeptide permease |  | 3.4 |  |
| BCAM2731 | putative membrane protein |  | 2.4 |  |
| BCAM2732 | putative membrane protein | 8.3 |  |  |
| BCAM2733 | putative acylphosphatase protein | -2.2 | 2.2 |  |
| BCAM2742 | conserved hypothetical protein | -2.2 |  |  |
| BCAM2744 | haemolysin-III related protein |  | 2.7 |  |
| BCAM2746 | carbon starvation protein A | -4.5 |  | -2.1 |
| BCAM2749 | carboxymuconolactone decarboxylase family | -2.6 |  |  |
| BCAM2754 | putative ketoreductase |  | 2.1 |  |
| BCAM2761 | giant cable pilus | -2.3 |  |  |
| BCAM2772 | putative class II aldolase | 2.1 |  |  |
| BCAM2774 | GntR family regulatory protein |  |  | -3.8 |
| BCAM2784 | putative aminotransferase |  |  | 2.1 |
| BCAM2786 | putative amino acid dehydratase/lyase |  |  | -3.0 |
| BCAM2827 | putative exported protein |  |  | 2.2 |
| BCAM2828 | putative membrane protein | -2.3 |  |  |
| BCAM2833 | squalene/phytoene synthase |  | 2.0 |  |
| BCAM2834 | conserved hypothetical protein | 2.1 |  |  |
| BCAM2840 | putative NADPH-dependent FMN reductase | -2.9 |  |  |
| BCAMr1023 | Perfect repeat flanking prophage | 2.7 | 2.8 | 6.3 |
| BCAS0031 | putative aminotransferase | -2.1 |  |  |
| BCAS0058 | putative oxidoreductase | -2.5 | 2.1 |  |
| BCAS0067 | AraC family regulatory protein | -2.1 |  |  |
| BCAS0069 | two-component regulatory system, response | 2.3 |  |  |
| BCAS0073 | putative hydrolase | 2.2 |  |  |
| BCAS0079 | non-heme chloroperoxidase |  | 2.0 |  |
| BCAS0080 | putative pyridine nucleotide-disulphide | 2.2 |  |  |
| BCAS0081 | ABC transporter ATP-binding membrane protein |  | 2.6 |  |
| BCAS0082 | serine peptidase, family S33 |  | 2.2 |  |
| BCAS0083 | TetR family regulatory protein |  |  | 2.2 |
| BCAS0086 | putative lipase | -2.1 |  |  |
| BCAS0104 | A-type flagellar hook-associated protein 2 |  | 2.6 | -2.2 |
| BCAS0109 | succinylglutamate desuccinylase/aspartoacylase | -2.3 |  |  |
| BCAS0110 | periplasmic solute-binding protein |  | -2.0 |  |
| BCAS0126 | MarR family regulatory | -2.7 |  |  |
| BCAS0132 | putative arginase family protein |  | 2.7 |  |
| BCAS0137 | putative aminotransferase | -2.2 |  | -2.9 |
| BCAS0138 | putative aldehyde dehydrogenase |  |  | -2.3 |
| BCAS0158 | putative exported protein |  | -2.0 |  |
| BCAS0174 | conserved hypothetical protein | -2.2 |  |  |
| BCAS0187 | putative amino acid efflux protein - LysE | -2.7 |  |  |
| BCAS0188a |  | -2.5 |  |  |
| BCAS0196 | putative polygalacturonase | 2.2 |  |  |
| BCAS0200 |  | -3.9 |  |  |
| BCAS0200 |  |  | 2.7 |  |
| BCAS0202 | putative membrane protein | -4.8 | 2.6 |  |
| BCAS0203 | ABC transporter protein | -6.1 | 2.0 | -2.5 |
| BCAS0204 | ABC transporter ATP-binding protein | -7.4 | 3.7 |  |
| BCAS0205 | TauD/TfdA taurine catabolism dioxygenase family | -4.1 |  | -2.6 |
| BCAS0206 | putative methyltransferase family protein | -7.2 | 2.5 | -2.4 |
| BCAS0207 | conserved hypothetical protein | -5.6 | 2.5 | -2.1 |
| BCAS0208 | putative acyl-CoA dehydrogenase | -7.0 | 2.8 | -2.4 |
| BCAS0209 | conserved hypothetical protein | -8.0 | 2.5 | -2.9 |
| BCAS0210 | putative AMP-binding enzyme | -6.7 | 2.0 | -2.8 |
| BCAS0211 | putative pyridoxal-dependent decarboxylase | -6.8 | 2.5 | -2.7 |
| BCAS0212 | conserved hypothetical protein | -6.1 | 2.4 | -2.7 |
| BCAS0213 | conserved hypothetical protein | -5.9 | 2.5 | -2.4 |
| BCAS0214 | conserved hypothetical protein | -6.5 | 2.5 | -2.6 |
| BCAS0215 | putative exported protein | -7.4 | 2.3 | -3.4 |
| BCAS0216 | putative acyl carrier protein | -8.3 | 2.1 | -3.7 |
| BCAS0217 | conserved hypothetical protein | -6.5 | 2.1 | -3.9 |
| BCAS0218 | hypothetical protein | -4.7 | 2.2 | -2.5 |
| BCAS0219 | putative exported protein | -4.1 | 2.4 | -2.2 |
| BCAS0220 | putative permease | -3.0 | 2.8 |  |
| BCAS0221 | ABC transporter ATP-binding protein | -3.6 | 2.9 |  |
| BCAS0223 | putative fatty acid desaturase | -3.7 | 2.1 | -2.2 |
| BCAS0224 | conserved hypothetical protein | -4.7 |  | -2.6 |
| BCAS0225 | LysR family regulatory protein | -3.6 |  | -2.3 |
| BCAS0226 | putative hydrolase | -2.2 | 2.2 |  |
| BCAS0234 | hybrid two component system kinase-response | -2.2 |  |  |
| BCAS0235 | two-component regulatory system, response | 2.4 | -2.9 |  |
| BCAS0237 | putative outer membrane protein | 2.2 |  |  |
| BCAS0251 | putative lipoprotein |  | 2.2 | 3.0 |
| BCAS0258 | GntR family regulatory protein | -9.0 |  | -7.0 |
| BCAS0259 | putative sodium:dicarboxylate symporter family | -4.3 |  | -6.2 |
| BCAS0260 | hypothetical protein | -3.4 |  | -4.6 |
| BCAS0262 | putative acetyltransferase | -2.9 |  |  |
| BCAS0267a | putative calcineurin-like phosphoesterase family | -2.1 |  |  |
| BCAS0281 | putative 2-hydroxy-3-oxopropionate reductase |  | 2.2 |  |
| BCAS0282 | hypothetical protein | -5.1 |  | -2.1 |
| BCAS0292 | conserved hypothetical protein | -137.3 |  | -278.2 |
| BCAS0293 | nematocidal protein AidA | -88.7 |  | -214.9 |
| BCAS0295 | glycosyltransferase | -2.0 |  |  |
| BCAS0317 | dienelactone hydrolase family protein | -2.0 |  |  |
| BCAS0318 | AraC family regulatory protein | -2.4 |  |  |
| BCAS0321 | hypothetical glycine-rich autotransporter | -2.5 |  |  |
| BCAS0355 | xylose isomerase |  |  | -2.9 |
| BCAS0363 | glyoxalase/bleomycin resistance | 2.1 |  |  |
| BCAS0383 | putative aminotransferase |  | -4.6 |  |
| BCAS0386 | putative haloacid dehalogenase-like hydrolase | -4.5 |  | -3.1 |
| BCAS0386 | putative haloacid dehalogenase-like hydrolase |  | -3.3 |  |
| BCAS0387 |  |  | -2.4 | -2.4 |
| BCAS0388 | putative periplasmic solute-binding protein |  | -3.0 |  |
| BCAS0396 | putative dehydrogenase |  | -2.2 | -2.4 |
| BCAS0397 | metallo peptidase, subfamily M20D |  |  | -2.3 |
| BCAS0399 | citrate-proton symporter |  | -2.1 |  |
| BCAS0401 | aerobic C4-dicarboxylate transport protein | 2.4 |  |  |
| BCAS0407 | hypothetical protein | -3.5 |  |  |
| BCAS0409 | zinc metalloprotease ZmpA | -5.3 | 4.0 | -4.5 |
| BCAS0431 | conserved hypothetical protein | 2.0 |  |  |
| BCAS0435 | putative short-chain dehydrogenase | 2.1 |  |  |
| BCAS0455 | putative CopG family protein |  | -2.1 |  |
| BCAS0460 | putative porin protein | -2.3 |  |  |
| BCAS0473 | efflux system transport protein | 2.2 |  |  |
| BCAS0497 | catechol 1,2-dioxygenase 2 |  | 2.6 |  |
| BCAS0503 | LysR family regulatory protein | 2.1 |  |  |
| BCAS0510 | hypothetical phage protein | -2.0 |  |  |
| BCAS0522 | hypothetical phage protein |  | -2.1 |  |
| BCAS0525 | putative phage Mu G protein | 2.0 |  |  |
| BCAS0545 | hypothetical phage protein | 2.2 |  |  |
| BCAS0553 | hypothetical phage protein | 2.0 |  |  |
| BCAS0556 | AraC family regulatory protein | 2.0 |  |  |
| BCAS0572 | putative exported protein |  | 2.6 |  |
| BCAS0573 | putative exported protein |  | 2.7 |  |
| BCAS0580 | conserved hypothetical protein |  | -2.0 |  |
| BCAS0581 | putative transcriptional regulatory protein |  | -2.3 |  |
| BCAS0586 | two-component regulatory system, response |  | 2.9 |  |
| BCAS0601 | conserved hypothetical protein |  | 2.0 |  |
| BCAS0604 | putative membrane protein |  |  | 2.2 |
| BCAS0605 | NmrA-like family protein |  |  | 3.1 |
| BCAS0608 | beta-ketothiolase | -3.2 |  |  |
| BCAS0625 | putative lipoprotein | -2.7 |  |  |
| BCAS0627 | putative lipoprotein |  |  | 2.5 |
| BCAS0632 | hybrid two component system kinase-response | -2.1 |  |  |
| BCAS0636 | conserved hypothetical protein | -2.4 |  |  |
| BCAS0637 | 60 kDa chaperonin 3 | -5.1 |  |  |
| BCAS0638 | 10 kDa chaperonin 3 | -3.2 | 2.1 |  |
| BCAS0640 | conserved hypothetical protein |  |  | -2.5 |
| BCAS0645_J_0 | putative short chain dehydrogenase (pseudogene) |  | 3.7 |  |
| BCAS0655 |  | 2.1 |  |  |
| BCAS0661B | conserved hypothetical protein | 2.2 |  |  |
| BCAS0664 | conserved hypothetical protein |  | 2.3 |  |
| BCAS0665 | putative membrane protein |  | 2.4 |  |
| BCAS0666 | putative ankyrin-repeat exported protein | -2.0 | 2.0 |  |
| BCAS0673 | conserved hypothetical protein |  | -2.4 |  |
| BCAS0675 | conserved hypothetical protein |  | -2.2 |  |
| BCAS0676 | hypothetical protein |  | -3.2 | -2.1 |
| BCAS0687 | putative membrane protein | 2.1 |  |  |
| BCAS0694 | putative carboxymuconolactone decarboxylase |  | 2.9 |  |
| BCAS0698 | putative alcohol dehydrogenase | -2.5 |  | -3.1 |
| BCAS0702 | putative substrate-binding transporter protein |  | 2.1 |  |
| BCAS0703 | putative short-chain dehydrogenase | -2.6 | 2.3 |  |
| BCAS0705 | putative pyridine nucleotide-disulphide | -4.4 |  |  |
| BCAS0706 | Major Facilitator Superfamily protein |  | 2.0 |  |
| BCAS0712 | AnsC family regulatory protein | -2.0 |  |  |
| BCAS0715 | LysR family regulatory protein |  | 2.0 |  |
| BCAS0724 | peptide methionine sulfoxide reductase family | -2.1 |  |  |
| BCAS0728 | cytidine deaminase |  | 2.8 |  |
| BCAS0737 | putative acetyl-CoA acetyltransferase | -2.5 | 2.7 |  |
| BCAS0738 | putative short-chain dehydrogenase family | -2.4 | 2.8 |  |
| BCAS0739 | putative acetyl-CoA synthetase | -2.5 | 2.1 |  |
| BCAS0746 | putative metal dependent phosphohydrolase | -2.1 |  |  |
| BCAS0750 | putative exported protein |  | 2.3 | 4.9 |
| BCAS0751 | putative gamma-glutamyltransferase precursor | -2.3 |  |  |
| BCAS0756 | putative phage integrase family protein | 2.3 |  |  |
| BCAS0759 | putative membrane protein | 2.1 |  |  |
| BCAS0762 | putative exported protein |  | -5.8 | -4.8 |
| BCAS0764 | multidrug efflux system outer membrane protein | 2.0 |  |  |
| pBCA010 | putative membrane protein |  | 2.4 | -2.2 |
| pBCA016 | conserved hypothetical protein | -4.0 |  | -2.8 |
| pBCA017 | hypothetical protein | 2.2 |  | -3.1 |
| pBCA018 | hypothetical protein | -3.6 |  |  |
| pBCA019 | putative membrane protein | 2.9 |  |  |
| pBCA022 | conserved hypothetical protein |  |  | -2.0 |
| pBCA024 | hypothetical protein |  |  | -2.6 |
| pBCA027 | putative conjugative transfer protein |  |  | -2.8 |
| pBCA029 | putative membrane protein |  |  | -2.0 |
| pBCA031 | putative TraU conjugative transfer protein | 2.1 |  |  |
| pBCA038 | hypothetical protein |  | 2.3 |  |
| pBCA044 | putative TraB conjugative transfer protein | 2.0 |  |  |
| pBCA055 | putative membrane protein | -12.0 | 2.4 | -9.1 |
| pBCA057 | putative conjugative transfer protein | -2.1 |  | -2.1 |
| pBCA061 | hypothetical protein | 2.1 |  |  |
| pBCA064 | putative membrane protein |  |  | -2.5 |
| pBCA087 | NUDIX hydrolase family protein |  |  | -7.2 |

^a^Function derived from *B. cenocepacia* J2315 (1).

^b^Change (fold) in *cepR*, *cciR* or *cepRcciIR* mutants compared to K56-2 determined by microarray analysis.

1**. Holden, M. T., H. M. Seth-Smith, L. C. Crossman, M. Sebaihia, S. D. Bentley, A. M. Cerdeno-Tarraga, N. R. Thomson, N. Bason, M. A. Quail, S. Sharp, I. Cherevach, C. Churcher, I. Goodhead, H. Hauser, N. Holroyd, K. Mungall, P. Scott, D. Walker, B. White, H. Rose, P. Iversen, D. Mil-Homens, E. P. Rocha, A. M. Fialho, A. Baldwin, C. Dowson, B. G. Barrell, J. R. Govan, P. Vandamme, C. A. Hart, E. Mahenthiralingam, and J. Parkhil**l. 2009. The genome o*f Burkholderia cenocepac*ia J2315, an epidemic pathogen of cystic fibrosis patients. J Bacterio**l 19**1:261-77.
