# Supplementary material for: Reciprocal regulation by the CepIR and CciIR quorum sensing systems in Burkholderia cenocepacia
Source: BMC Genomics. 2009 Sep 17;10:441. doi: 10.1186/1471-2164-10-441 (PMC2753556; doi:10.1186/1471-2164-10-441)
Supplement: Additional file 2 — Oligonucleotide primers used in this study. Complete list of oligonucleotide primers used in this study. [file 1471-2164-10-441-S2.DOC]

Additional File 2. Oligonucleotide primers used in this study.

| Primer | Sequence (5’-3’) | Reference |
| --- | --- | --- |
| F1-M1868-UP-EcoRI | GCCTGAATTCTTGTCGCGCAGTTCGGCC^a^ | This study |
| R1-M1868-UP-ClaI | GCTTATCGATTGGTAGTGCGCCATCCAG^b^ | This study |
| F2-M1868-DW-ClaI | GCTTATCGATAAGACCGCGTGCGAAATC^b^ | This study |
| R2-M1868-DW-XbaI | GCACTCTAGACGCACGCTGCTGTCGAACGC^c^ | This study |
| sigAqRTfor1 | AGGAAACCAACCGTCAGATG | This study |
| sigAqRTrev1 | GCGACGGTATTCGAACTTGT | This study |
| EBS-E62 (zmpA(RT)-f) | CGCGGCAGACATCGACTAC | (1) |
| RBS-E62 (zmpA(RT)-r) | AGATGCCGTTGCGGTTGT | (1) |
| BCAL1814qRTfor1 | ATGCGGCTGAAAGTGGGAGA | This study |
| BCAL1814qRTrev1 | ATTTCGGCGAGCGACAGTCC | This study |
| BCAM0189RTfor1 | ccgggcaactattgtttacg | (2) |
| BCAM0189RTrev1 | ccgagatagagcgagcagag | (2) |
| BCAM0191RTfor2 | aacatcatggcgttcgacta | (2) |
| BCAM0191RTrev1 | gctgtagtactgcggactcg | (2) |
| BCAM0199RTfor1 | accttgccgagctgaactac | (2) |
| BCAM0199RTrev1 | gtcgagcgagacctgctg | (2) |
| BCAS0293RTfor1 aidA | CTACAAGTTCATCGGCAACG | This study |
| BCAS0293RTrev1 aidA | GAACGGATCCCACGAGAAG | This study |
| BCAL0114RTfor1 fliC | GCGTGTCGATGATTCAAACGGCAT | This study |
| BCAL0114RTrev1 fliC | TCACTTCCTGGATCTGCTGCGAAA | This study |
| BCAS0225qRTfor shvR | agtctgccttcgctggatt | D. T. Nguyen |
| BCAS0225qRTrev shvR | cacgcgtgaagcttaaatgg | D. T. Nguyen |
| BCAS0220RTfor2 | GAACCAGTTCTCGGTGTTCG | This study |
| BCAS0220RTrev2 | GATCCAGTTGCTCATCGACA | This study |
| BCAS0204RTfor1 | GCAATTGCAGAAGGTCGAGT | This study |
| BCAS0204RTrev1 | CGACGATCTCGGATACACG | This study |
| BCAM1418RTfor4 | TACCTGACCAAGCCGTTCTC | This study |
| BCAM1418RTrev3 | GAGATCAGCGTCGATTCCTG | This study |
| BCAM1420RTfor1 | ATGGAAGAGAAACATCACAGCGCC | (2) |
| BCAM1420RTrev1 | ATGCCGTGTTCGTCTACCTGGAT | (2) |
| AfcAPromfor1 | ggctcgaggggttgcaattcttctgtgag^d^ | This study |
| AfcAPromrev1 | ggggatccatcagacggtgaatggtgga^e^ | This study |

Restriction endonuclease site is incorporated into primers as underlined on sequence: EcoRI^a^, ClaI^b^, XbaI^c^, XhoI^d^ or BamHI^e^.

1. **Gingues, S., C. Kooi, M. B. Visser, B. Subsin, and P. A. Sokol.** 2005. Distribution and expression of the ZmpA metalloprotease in the *Burkholderia cepacia* complex. J Bacteriol **187:**8247-8255.

2. **Malott, R. J., E. P. O'Grady, J. Toller, S. Inhulsen, L. Eberl, and P. A. Sokol.** 2009. A *Burkholderia cenocepacia* orphan LuxR homolog is involved in quorum-sensing regulation. J Bacteriol **191:**2447-60.
